# Late Pleistocene-Holocene paleobiogeography of the genus *Apodemus* in Central Europe

**DOI:** 10.1371/journal.pone.0173668

**Published:** 2017-03-10

**Authors:** Markéta Knitlová, Ivan Horáček

**Affiliations:** Department of Zoology, Charles University, Viničná 7, Praha 2, Czech Republic; National Cheng Kung University, TAIWAN

## Abstract

Wood mice of the genus *Apodemus* are an essential component of small mammal communities throughout Europe. Molecular data suggest the postglacial colonization of current ranges from south European glacial refugia, different in particular species. Yet, details on the course of colonization and Holocene history of particular species are not available, partly because of a lack of reliable criteria for species identification in the fossil record. Using a sample of extant species, we analyzed variation patterns and between-species overlaps for a large set of metric and non-metric dental variables and established the criteria enabling the reliable species identification of fragmentary fossil material. The corresponding biometrical analyses were undertaken with fossil material of the genus (2528 items, 747 MNI) from 22 continuous sedimentary series in the Czech Republic and Slovakia, from LGM to Recent. In Central Europe, the genus is invariantly absent in LGM assemblages but regularly appears during the Late Vistulian. All the earliest records belong to *A*. *flavicollis*, the species clearly predominating in the fossil record until the Late Holocene. *A*. *uralensis* accompanied it in all regions until the late Boreal when disappeared from the fossil record (except for Pannonia). A few items identified as *A*. *sylvaticus* had already appeared in the early Holocene assemblages, first in the western part of the region, yet the regular appearance of the species is mostly in the post-Neolithic age. *A*. *agrarius* appeared sparsely from the Boreal with a maximum frequency during the post-Neolithic period. The results conform well to the picture suggested by molecular phylogeography but demonstrate considerable differences among particular species in dynamic of the range colonization. Further details concerning Holocene paleobiogeography of individual species in the medium latitude Europe are discussed.

## Introduction

Wood mice of the genus *Apodemus* rank among the most common rodents of the Palaeartic region. Often, they are the eudominant component of local communities of small ground mammals throughout the whole of Europe [1]. The genus, phenotypically established within the early radiation of murid rodents during the Vallesian [2–3], comprises of 20 extant species subdivided into 3–4 subgenera: *Apodemus*, *Sylvaemus*, *Argenteus* and (*Gurkha*) [4–5]. Three of these diverged in Eastern Asia (*Apodemus*, *Argenteus* and *Gurkha* [6–9]). All the West Palearctic representatives except for *A*. *agrarius* (subgenus *Apodemus*) belong to the subgenus *Sylvaemus*, the clade regularly represented in the European fossil record since the Turolian. Presumably, it was already established there in the Late Miocene (MN13 [2, 10, 11]), shortly after the westward expansion of the murids in MN10. In contrast, all extant species of the subgenus *Sylvaemus* (including the European representatives, *A*. *alpicola*, *A*. *flavicollis*, *A*. *mystacinus*, *A*.*epimelas*, *A*. *sylvaticus* and *A*. *uralensis)* are, according to molecular data [6, 12–15], mutually closely related and separated by shallow divergences only (less than 10% in mtDNA), which suggests their radiation from a common ancestor during the Pliocene and Early Pleistocene age supposedly under the effects of the climatic rearrangements of that time [16].

The appearance of the genus is reported from a vast majority of the Quaternary fossil sites throughout Europe: Kowalski [17] listed it in 460 assemblages from 24 countries. In Central Europe, the genus *Apodemus* is obviously absent in glacial assemblages (at least those of the Middle to Late Pleistocene age), while it presents a euconstant element of interglacial assemblages, a fact which even led to it being declared an index fossil of the interglacial stages, including the Holocene [18].

In most instances, the fossil *Apodemus* was coidentified with the medium-sized form, *A*. *sylvaticus* (227 records in Kowalski [17]), while the other species were identified in the fossil record only very rarely (Kowalski [17]: 31 in *A*. *flavicollis*, 7 *A*. *agrarius*, none of *A*. *uralensis* and *A*. *alpicola*).

Yet, just in these connections, it should be stressed that within the subgenus *Sylvaemus*, which is quite uniform in phenotypic respects, identification at a species level traditionally presents a significant problem. Though individual species differ in mean body size, each of them shows a broad range of within-species variation and extensive between-species overlaps in the states of any morphometric traits (comp. e.g. [19–24]). Just recently, with regards to the discriminatory bias of morphometric characters, differences in the distress call were proposed as the most reliable criterion of species identification [25]. Obviously, the species identification is even more complicated in the case of fragmentary fossil materials, which are often restricted to isolated molar teeth [26, 27]. It is no wonder that the species identification of fossil *Apodemus* was frequently regarded as provisional (cf.) and/or not provided at all (119 records in Kowalski [17]).

At the same time, any information from the fossil record on the past dynamics of species ranges and differences among particular species in these respects are urgently needed, at least as an independent contextual variable supplementing the robust molecular data on that subject. In contrast to other interglacial elements (such as *Clethrionomys glareolus*, *Sorex araneus*, *Microtus agrestis*, *Microtus subterraneus*) for which survival in the glacial refugia in Central Europe was demonstrated both by a reliable fossil record and molecular data, *Apodemus* spp. represent a rare case for which the paradigmatic scenario of range history (i.e. no survival in Central Europe during glacials and the recolonization of present-day ranges via post-glacial expansion from the southern refugia) has not been disproved. Moreover, molecular studies suggest different glacial refugia and different trajectories of post-glacial range expansion for *A*. *flavicollis* (with its Vistulian range restricted to the Balkans) and for *A*. *sylvaticus* (the Iberian Peninsula and southwestern France) [28–31].

The present contribution is intended to respond to these tasks. With the aid of a detailed morphometric study, (i) we established a procedure enabling reliable species identification and (ii) applied it to a large set of fossil material of the genus obtained from continuous sedimentary series of the Late Pleistocene and Holocene age from various regions of the Czech Republic and Slovakia. (iii) The paleobiogeographic inferences resulting from our comparisons are in good accord with the predictions of molecular phylogeography, but suggest considerable between-species differences in the timing of the range expansion and the role of particular species in actual communities.

## Material and methods

The present paper is essentially based on detailed phenotypic analyses of dental material. Both the fossil and Recent samples surveyed in this study originate from the Czech Republic (CZ) and Slovakia (SK) and are deposited in the collections of the Department of Zoology, Charles University, Prague. The materials were collected within the period 1975–2013, mostly from excavations undertaked by the authors under all then valid rules and permissions. No further permits were required for the described study.

The comparative sample of extant populations, for which species identification employed a combination of standard external, cranial, and karyological criteria (comp. e.g. [32, 33]), included 225 individuals: 23 *A*. *agrarius* (Liberec, N Bohemia, CZ); 75 *A*. *flavicollis* (34 Novohradské Mts., S Bohemia, CZ; 10 Teplice, NE Bohemia, CZ; 18 Lesser Fatra Mts., SK; 13 Vihorlat Mts., SK); 63 *A*. *sylvaticus* (49 Prague, CZ; 14 Novohradské Mts., S Bohemia, CZ); and 64 *A*. *uralensis* (23 Znojmo, S Moravia, CZ; 13 Hodonín, S Moravia, CZ; 6 Břeclav, S Moravia, CZ; 22 Košice, SK). The study also included 23 specimens of *A*. *uralensis cimrmani* Vohralík, 2002, a relic form isolated in NW Bohemia [34], (Letov, N Bohemia, CZ, coll. Vohralík V).

The fossil material surveyed in this study comes exclusively from well-stratified continuous sedimentary series (mostly in cave entrances and scree deposits under rocky walls and mainly covering the period from the Late Vistulian to the Recent) excavated for biostratigraphical purposes. The techniques of site excavation, field sampling, and extraction, and quantitative analyses of fossil material followed standards proposed by Ložek [35, 36] and Horáček and Ložek [18]. This also concerns the stratigraphical assessment of individual community samples (obtained from particular layers of the respective sedimentary series), which is expressed here in terms of the Late Vistulian-Holocene mollusc and vertebrate biozones A-F [18], roughly corresponding to the stages of standard subdivison [37], viz. A–Late Vistulian (after the LGM), B–Preboreal, C–Boreal, D–Atlantic, E–Epiatlantic, F–Late Holocene.

**Fig 1:**
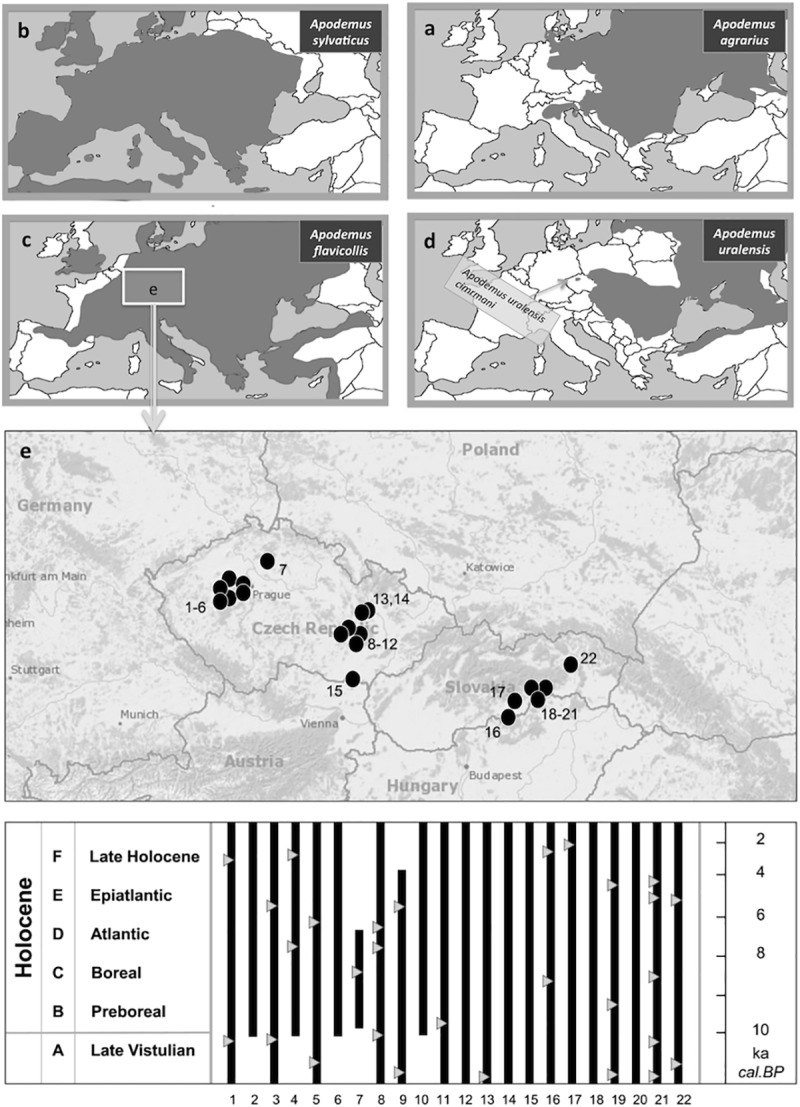
**The current European ranges of *Apodemus agrarius* (a), *A*. *sylvaticus* (b), *A*. *flavicollis* (c), and *A*. *uralensis* (d); geographic position of the source sedimentary sequences of the studied fossil material (e), and their stratigraphical span (with available 14C data–triangles).** Source data for (a)-(d) taken from [86–89].

For the purposes of this study, we re-examined 310 fossil community samples from 52 sites regarding the presence of the genus *Apodemus* (for a list of the sites, see [18, 38, 39]). The remains of the genus (mostly isolated teeth and jaw fragments) from 115 community samples of the 22 most representative sedimentary series with respect to the appearance of the genus and time coverage were analyzed in detail. A list of the respective sites is in Table 1; further details (including 14C data, which, in general, confirm the biostratigraphic dating–comp. [40] for details) are in S1 Fig and Fig 1.

**Table 1:** Source sections of the material examined in this study.

No	Locality abbr.	Name (administrative localisation, region)	N latitude	E longitude	Number of layers	14C data	MNI small mammals	MNI *Apodemus*	*Apodemus spp*.			
									M1	M2	m1	m2
CZECH REPUBLIC—BOHEMIA												
1	AKSA	Aksamitova brána (Tmaň, Bohemian Karst)	49°54'10.909''N	14°6'6.495''E	8	2	169	31	19	8	27	13
2	BACI	Bacín (Vinařice, Bohemian Karst)	49°53'49.567''N	14°6'12.280''E	18		550	64	35	18	59	31
3	MART	Martina (Tetín, Bohemian Karst)	49°56'33.400''N	14°6'34.352''E	16	3	821	71	112	56	135	85
4	SKAC	Skalka nad Čihovou (Karlštejn, Bohemian Karst)	49°54'26.378''N	14°10'45.138''E	9	2	268	44	62	32	58	42
5	SKAM	Skalice (Měňany, Bohemian Karst)	49°54'10.909''N	14°6'6.495''E	9	3	256	25	8	3	9	8
6	ZELE	Železná (Mramor, Bohemian Karst)	49°53'56.247''N	14°7'10.631''E	7		105	21	5	2	9	3
7	PCER	Pod Černou louží (Dřevčice, Česká Lípa)	50°35'6.314''N	14°30'22.817''E	7	3	85	21	2	1	13	8
CZECH REPUBLIC—MORAVIA												
8	BARO	Barová (Habrůvka, Moravian Karst)	49°18'30.672''N	16°41'36.658''E	17	5	616	22	12	3	12	4
9	HOLS	Holštejnská (Holštejn, Moravian Karst)	49°24'11.069''N	16°46'29.902''E	8	2	514	6	5	4	18	7
10	NEMC	Němcova (Suchdol, Moravian Karst)	49°23'7.794''N	16°43'15.793''E	8		150	16	10	7	20	5
11	SRNC	Srnčí (Ostrov, Moravian Karst)	49°22'14.329''N	16°44'15.271''E	13	1	719	17	9	7	13	9
12	ZAZD	Zazděná (Vavřinec, Moravian Karst)	49°22'21.079''N	16°43'31.594''E	14		687	17	2	3	4	2
13	ZKAZ	Zkamenělý zámek (Javoříčko, Olomouc distr.)	49°38'27.970''N	16°54'13.588''E	12	1	700	14	0	0	2	1
14	PRUC	Průchodnice (Ludmírov, Olomouc distr.)	49°40'3.682''N	16°53'17.395''E	9		128	14	4	1	9	6
15	MARK	Martinka (Horní Věstonice, Břeclav distr.)	48°51'58.832''N	16°38'9.264''E	9	2	72	11	2	0	2	0
SLOVAKIA												
16	MEDV	Mara Medvedka cave (Divín, Lučenec)	48°27'47.617''N	19°31'39.841''E	9	2	191	22	21	11	20	14
17	PESK	Peskö (Bretka, Rimavská Sobota distr.)	48°29'56.130''N	20°20'20.958''E	17	2	906	27	18	9	31	17
18	CESK	Červená Skala (Silická Jablonica, Slovak Karst)	48°32'34.293''N	20°28'46.429''E	6		116	22	45	27	46	34
19	HAMO	Hámorská (Plešivec, Slovak Karst)	48°33'41.611''N	20°24'25.799''E	13	3	273	65	32	11	74	57
20	CEMN	Červeného mnícha (Jovice, Slovak Karst)	48°37'24.673''N	20°31'49.655''E	7		180	44	49	31	55	29
21	MAST	Maštalná (Brzotín, Slovak Karst)	48°35'57.843''N	20°27'45.641''E	17	6	1831	161	248	163	326	93
22	RUZI	Velká Ružínská jask. (Ružín, NE Slovakia)	48°51'34.474''N	21°6'37.903''E	10	2	123	12	7	2	7	5
**TOTAL**					**243**	**39**	**9460**	**747**	**707**	**399**	**949**	**473**

The fossil material under study thus comprised of 2528 molars (707 M1, 399 M2, 949 m1, 473 m2) representing at least 747 individuals (MNI). All items were photographed with the aid of an Olympus SZX12 stereomicroscope, and further measured using tpsDig image analysis software (by Rohlf FJ) to an accuracy of 0.01 mm.

**Fig 2:**
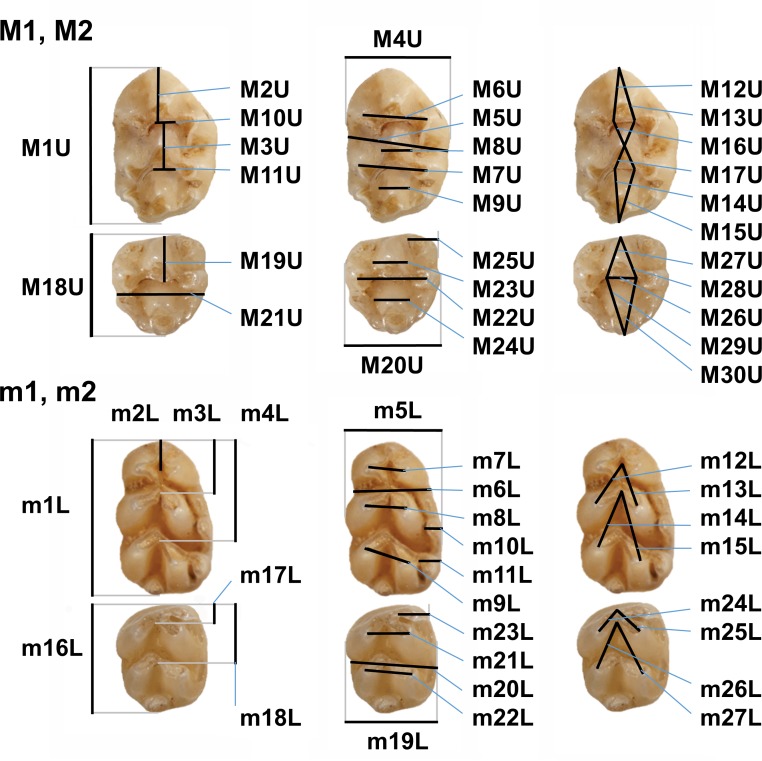
Definition of the metric characters applied in this study.

In total, 57 dental dimensions were measured (see Fig 2), supplemented with 4 proportional ratios (M2U/M1U, M14U/M15U, m3L/m1L, m6L/m5/L). The supplementary variables expressing the size of molar sufaces (SURM1, SURM2, SURm1, SURm2) were computed by multiplying molar length by molar width.

Further, the degree of tooth wear and the states of 24 non-metrical characters were scored using predefined scales (0–9) subdividing the span of the observed variation (see S1 Fig for details). All measurements were taken by the same person (M.K.). Maxillary molars are marked in upper case (M1-M3) and mandibular molars in lower case (m1-m3). Cusp nomenclature follows Horáček et al. [11].

Statistical analyses were performed in Microsoft Excel and Statistica Software 13.

The procedure of species identification included (i) analyses of the variation of each particular character and the patterns of between-species overlaps in the comparative sample of Recent populations (S2 Table) and (ii) the determination of the discriminatory capacity of particular variables (S3 and S4 Tables) and the discrimination criteria suggested by the discrimination analysis (S4 Table). Since no procedure succeeds in removing the effects of between-species overlap, we establish an alternative approach to species identification: individuals falling in zones of overlap and those exhibiting the character states restricted to particular extant species were treated as different parataxa. Further, we established the criteria most robustly distinguishing particular parataxa and applied them to the identification of fossil material.

## Results

### (1) Between-species overlaps and the identification procedure

The basic statistics of the morphometric variables in the Recent samples of particular species and a survey of the between-species overlaps are presented in S2 Table. Within the studied species of the subgenus *Sylvaemus* (*A*. *flavicollis*, *A*. *sylvaticus*, *A*. *uralensis*), the extent of between-species overlap varied for particular metrical variables from 10.2% (M1U) to 98.5% (m17L) of the total variation span, and from 77% to100% for the non-metrical characters. The mean character overlap was 51.88% for the metric characters (43.81 for M1, 50.75 for M2, 55.38 for m1, 56.42 for m2) and 98.1% for the non-metric characters. Only 9 metrical variables exhibited a less than 30% overlap.

In terms of the percentage of individuals falling in the zones of between-species overlaps, the smallest values appeared with the following variables: M1U (16.3% individuals), m1L (27.3%), SURM1 (18.8%), and SURM2 (29.1%). Between *A*. *flavicollis* and *A*. *sylvaticus* individuals, the smallest overlaps were found for M1U (9.4%), M5U (28.2%), M18U (26.7%), M21U (24.4%), M29U (23.3%), M30U (19.4%), m1L (11.3%), m16L (22.0%), m19L (27.8%), m20L (24.4%), SURM1 (13.5%), SURm1 (9.8%), SURm2 (15.0%), and between *A*. *sylvaticus* and *A*. *uralensis* individuals, for M1U (15.1%), M4U (18.2%) M5U (29.0%), M20U (26.5%), m1L (28.6%), SURM1 (15.4%), SURM2 (24.8%).

**Fig 3:**
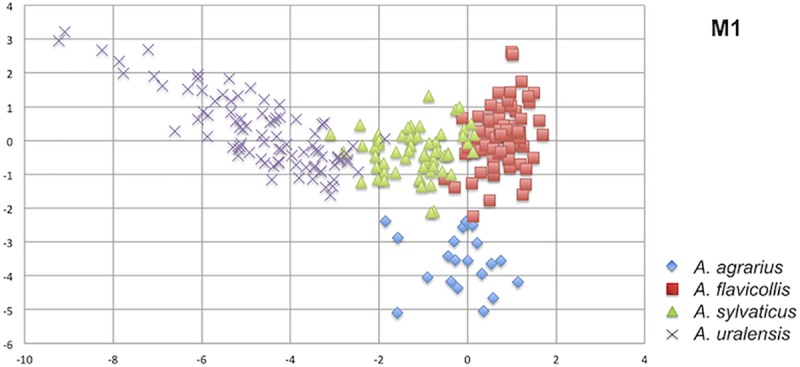
Plot of discrimination scores (R1/R2) of Recent *Apodemus* spp. based on metric variables of M1 (M1U –M17U). Note the distinct position of *A*. *agrarius* and the considerable overlap among particular species of the subgenus *Sylvaemus*.

**Fig 4:**
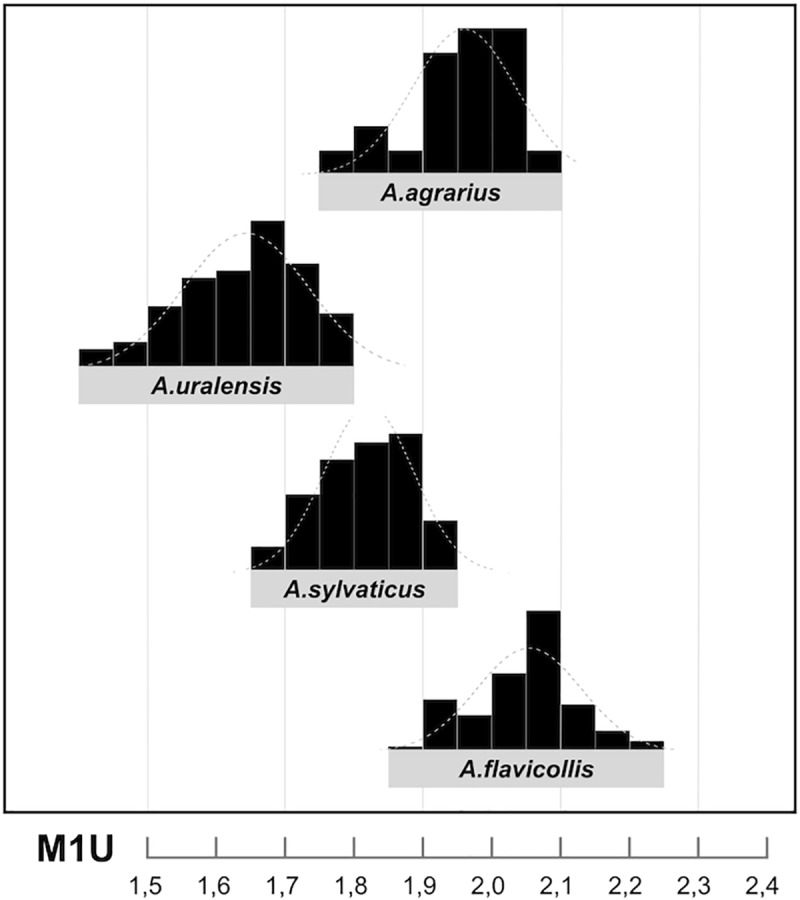
Frequency diagram of metric variation (M1 length = M1U) in the Recent sample of particular *Apodemus* spp. from the Czech Republic.

Discrimination analyses of the Recent samples combining a diverse set of variables demonstrated that: (i) *A*. *agrarius* differs robustly from the subgenus *Sylvaemus* on the basis of non-metric variables, though the discrimination based on metric characters was less obvious; (ii) the functions exhibiting the most robust discriminatory power for particular species of the subgenus *Sylvaemus* (S4 Table) are essentially due to the variables showing the least degree of between-species overlap and the most pronounced effects in ANOVA analyses categorized by species and parataxon variables (S3 Table)–viz. the length and width dimensions of individual molars (M1U, M4U, M18U, M20U, m1L, m5L, m16L, m19L). In contrast, the non-metric variables exhibited a negligible discrimination capacity only. (iii) Even the most robust functions retained a considerable proportion of undetermined individuals (Fig 3); we did not find a function capable of distinguishing more than 90% of individuals.

(iv) The discrimination capacity of multivariate functions does not essentially exceed that of the basic metrical dimensions—the length of individual molars (M1U, M18U, m1L, m16L) and their surface areas (SURM1, SURM2, SURm1, SURm2). (v) On the basis of frequency analyses (Fig 4), we established ranges of species-specific values and the between-species overlaps for each variable and used them as diagnostic criteria for the respective parataxa (Table 2), the classes applied further in the determination of fossil material. It is worth mentioning that the states of these variables do not appear to be significantly influenced by tooth wear (S4 Table).

**Table 2:** The most robust discrimination characters and their variation ranges separating the zones of between-species overlaps (parataxa 2 and 4) based on the Recent sample (n = 225 skulls, 889 teeth).

	*A*.*uralensis* parataxon 1	*A*.*ural*.*/A*.*sylv*. parataxon 2	*A*.*sylvaticus* parataxon 3	*A*.*sylv*.*/A*.*flav*. parataxon 4	*A*.*flavicollis* parataxon 5
M1U (M1 length)	1,42–1,68	1,69–1,77	1,78–1,89	1,90–1,95	1,96–2,22
M18U (M2 length)	0,92–1,04	1,05–1,17	1,18–1,19	1,20–1,32	1,33–1,50
m1L (m1 length)	1,39–1,51	1,52–1,64	1,65–1,74	1,75–1,81	1,82–2,05
m16L (m2 length)	0,96–1,01	1,02–1,16	1,17–1,18	1,19–1,28	1,29–1,43
M1U x M4U (M1 area)	1,43–1,93	1,94–2,11	2,12–2,33	2,34–2,52	2,53–3,31
M18U x M20U (M2 area)	0,93–1,16	1,17–1,35	1,36–1,38	1,39–1,65	1,66–2,03
m1L x m5L (m1 area)	1,31–1,46	1,47–1,74	1,75–1,95	1,96–2,08	2,09–2,63
m16L x M19L (m2 area)	0,90–0,93	0,94–1,21	1,22–1,25	1,26–1,39	1,40–1,79

The procedure applied in species identification of the fossil record was thus as follows. (1) The separation of *A*. *agrarius* (parataxon 8) from *Sylvaemus* based on distinct phenotypic differences: a slender M1 with a pronounced elongated cusp t2; M2 with completely reduced cusp t3; slender m1, m2 with indistinct labial cingulum.

(2) The categorization of all items belonging to the subgenus *Sylvaemus* under parataxa 1–6 based on the abovementioned morphometric characters (1- *A*. *uralensis*, 2 –*A*. *uralensis/A*. *sylvaticus*, 3 –*A*. *sylvaticus*, 4 –*A*. *sylvaticus/A*. *flavicollis*, 5 –*A*. *flavicollis*, 6 –*A*. *flavicollis* exceeding the upper limit of the variability of the Recent population). For each item, three different determination techniques were applied independently: SPA—preliminary identification “by eye” based on overall phenotype appearance; SPB–categorization based on molar length variables; and SPC–categorization based on area variables.

The results of the particular determination strategies SPA, SPB, SPC were compared by means of correlation analysis with the following results: r = 0,92 SPA:SPB; r = 0,93 SPA:SPC, r = 0,93 SPB:SPC for M1; r = 0,74 SPA:SPB; r = 0,71 SPA:SPC, r = 0,91 SPB:SPC for M2; r = 0,93 SPA:SPB; r = 0,92 SPA:SPC, r = 0,92 SPB:SPC for m1; r = 0,90 SPA:SPB; r = 0,93 SPA:SPC, r = 0,90 SPB:SPC for m2.

### (2) *Apodemus* in the Late Vistulian-Holocene fossil record

**Fig 5:**
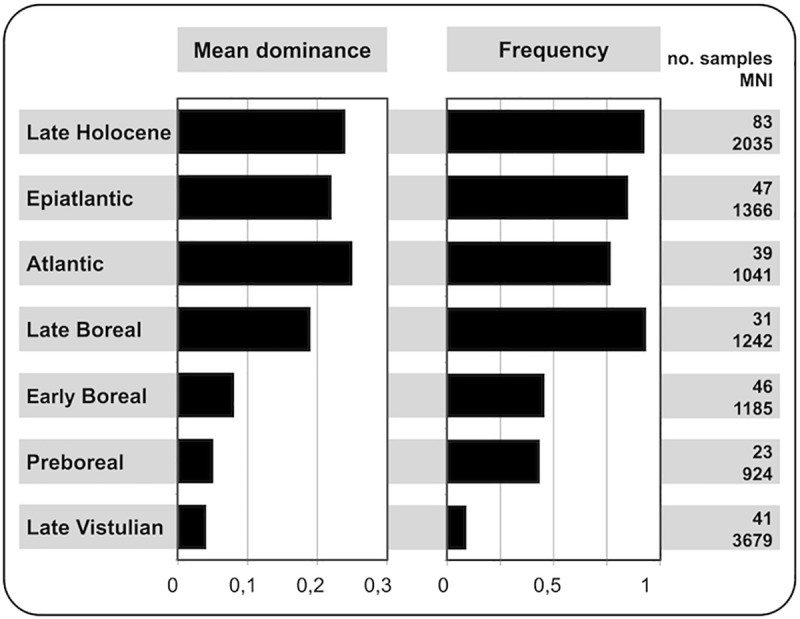
Mean dominance (based on MNI) and frequency of *Apodemus* spp. in the community samples representing particular stages of Late Pleistocene / Holocene stratigraphy–based on the sedimentary series from the Czech Republic and Slovakia (see [18] and Table 1 for a detailed list of them).

The genus *Apodemus* is invariantly absent in the glacial assemblages available from the Czech Republic and Slovakia (see also [18]). Then, it appears in the fossil record during the Late Vistulian, first in the Pannonian part of Slovakia. Until the Boreal, it is quite a rare (with a mean dominance <0.05), while since the late Boreal it has become a dominant community component (mean dominance >0.2) throughout the whole region (Fig 5).

**Fig 6:**
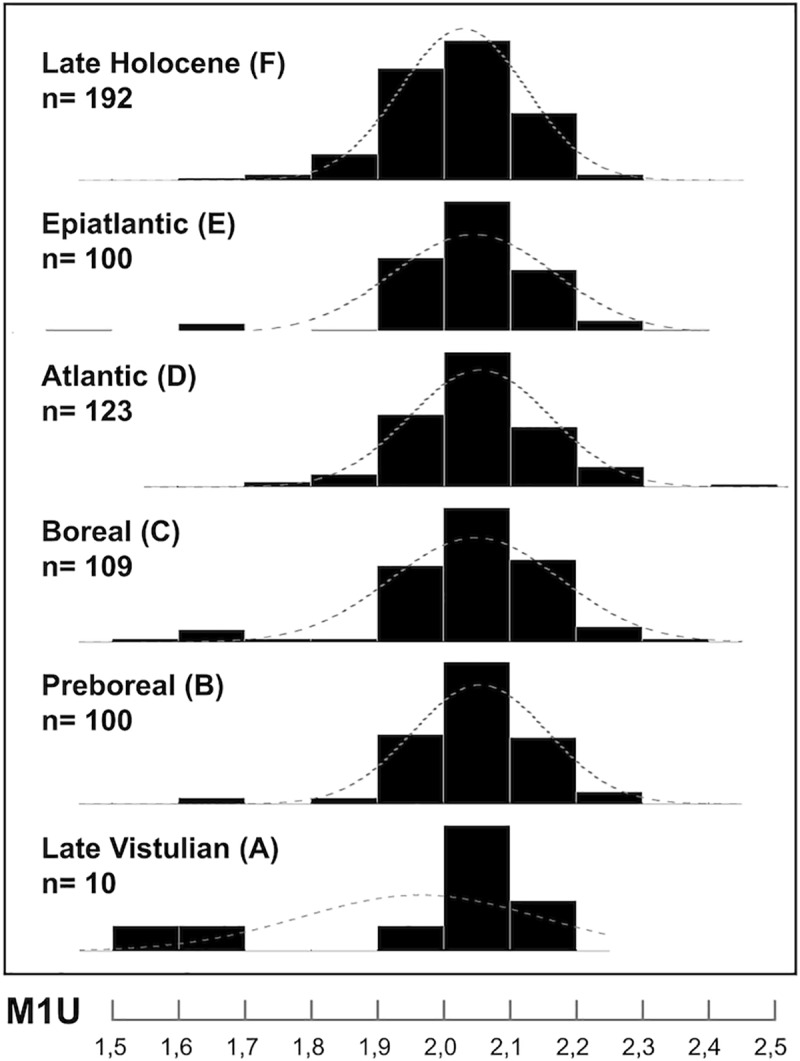
Frequency diagram of metric variation (M1 length = M1U) in samples of *Apodemus (Sylvaemus)* spp. representing particular stages of Late Pleistocene / Holocene stratigraphy.

**Fig 7:**
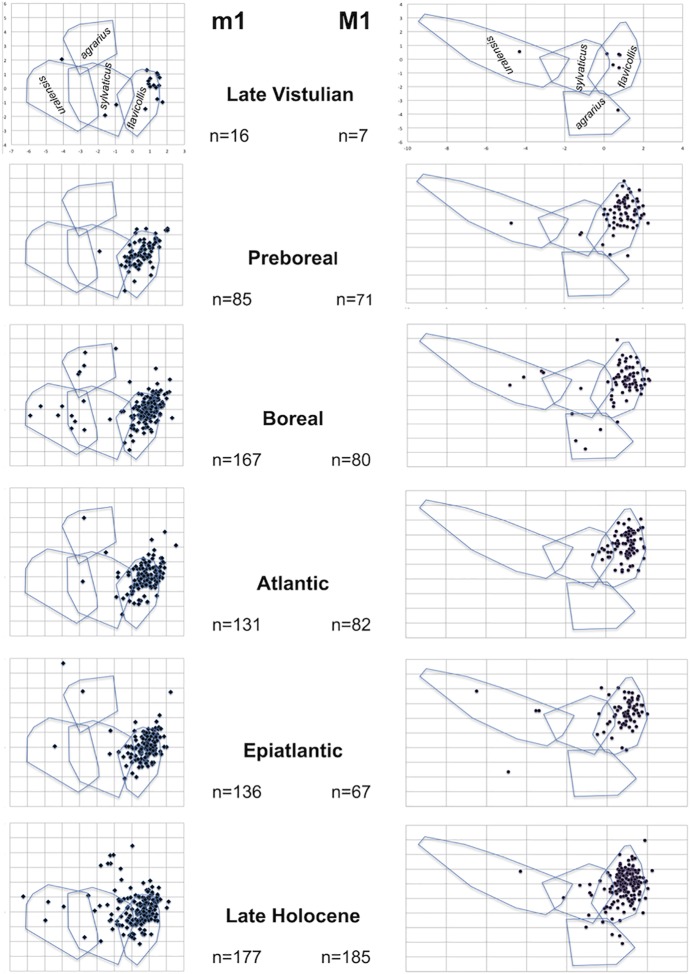
Plot of discriminant scores (R1/R2) of individual M1 (right) and m1 (left) teeth of *Apodemus* spp. from particular stages of Late Pleistocene / Holocene stratigraphy superimposed onto a plot of variation ranges in the respective variables for the Recent *Apodemus* sample (based on the discrimination analysis of metric variables of M1 and both metric and non-metric variable of m1). Note the extensive predominance of *A*. *flavicollis* phenotypes.

In the total of 2397 fossil molars examined, 131 teeth were not identified due to damage or a high degree of tooth wear. On the basis of morphometric criteria (SPB, SPC), the vast majority of the Late Pleistocene / Holocene record was assigned to parataxa 4,5,6, *A*. *flavicollis s*.*l*., while the other species were clearly less abundant (see also the results of the frequency analysis of M1U values in Fig 6). The same picture also arose from the application of discriminatory functions computed on the basis of the Recent samples (Fig 7).

A detailed survey of the determination results for particular community samples is in S5 Table. It is summarized in Table 3 in regards to the particular stratigraphic horizons and geographic regions under study.

**Table 3:** Parataxa of *Apodemus* (1–5, 8) in the Late Pleistocene/Holocene fossil record from particular biostratigraphic horizons (A-F) in the Czech Republic and Slovakia (n-number of molars, LOC-number of community samples). 1 –*A*.*uralensis*, 3 –*A-sylvaticus*, 5 –*A flavicollis*, 8 –*A*.*agrarius*.

region		CZECH REPUBLIC						SLOVAKIA					
parataxon:		1	2	3	4	5	8	1	2	3	4	5	8
**F**	**n**	0	1	23	7	94	0	6	1	32	63	484	33
**(Late Holocene)**	**LOC**	0	1	6	3	10	0	3	1	9	10	16	8
**E**	**n**	4	2	11	17	89	3	3	2	4	18	259	6
**(Epiatlantic)**	**LOC**	2	1	3	4	7	2	1	2	3	8	14	3
**D**	**n**	0	0	6	13	175	0	2	0	5	10	158	3
**(Atlantic)**	**LOC**	0	0	4	6	14	0	2	0	3	3	8	1
**C**	**n**	10	3	0	12	200	0	9	3	3	13	217	16
**(Boreal)**	**LOC**	5	1	0	6	19	0	5	3	2	4	9	2
**B**	**n**	4	0	3	24	241	0	0	0	0	1	53	0
**(Preboreal)**	**LOC**	2	0	1	3	5	0	0	0	0	1	3	0
**A**	**n**	3	4	0	4	38	0	0	0	0	1	1	0
**(Late Vistulian)**	**LOC**	1	2	0	1	4	0	0	0	0	1	1	0

For particular parataxa it can be summarized as follows:

**Parataxon 1**—*A*. *uralensis* s.str.: 41 molars (15 M1, 5 M2, 18 m1, 3 m2) in 21 community samples (Aksamitova brána 170–250; Bacín I/3, I130-140, I230; Červená skala 6; Červeného mnícha 4b, 5; Martina 11´, 12´; Martinka R2; Maštalná 0, 1; Peskö 2, 3, 4, 4a, 4b, 6; Skalice 4; Skalka 4; Srnčí 3); first appereance datum (FAD) in the Czech Republic (CZ): Preboreal–Martina 12´, FAD Slovakia (SK): Late Boreal–Peskö 4b and Červená skala 6 (biozone C2).

**Parataxon 2**—*A*. *uralensis/A*. *sylvaticus*: 16 molars (6 m1, 10 m2) in 10 community samples (Červená skala 5,6; Červeného mnícha 2, Hámorská 5; Martina 13´; Medvedka 6; Skalice 4; Skalka 1, 3; Peskö 6)

**Parataxon 3**—*A*. *sylvaticus* s.str: 87 molars (24 M1, 8 M2, 50 m1, 5 m2) in 31 community samples (Aksamitova brána 50–70; Bacín I/A2; Barová A1; Červená skala II/3, 3; Červeného mnícha 5; Hámorská 1, 2, 3; Holštějnská 1; Martina 140–170, portal E; Maštalná 0, 1, 2, 4/II, 6, 7, 8, 9; Medvedka 2, 3; Němcova 3; Peskö 3; Průchodnice I5, 3; Skalka 1, 2, 3, 4, 5); FAD CZ: Preboreal/Boreal—Martina 11´, FAD SK: Late Boreal—Maštalná 9 (biozone C2).

**Parataxon 4** –*A*. *sylvaticus/A*. *flavicollis* (parataxon 4)—183 molars (52 M1, 41 M2, 65 m1, 25 m2) in 50 community samples (Aksamitova brána 50–70, 170–250; Bacín I/3, I3 130–140; Barová A1, 5; Červená skala II/3, II/4, 4, 6; Červeného mnícha 4b, 5; Hámorská 1, 2, 3, 4, 5, 12; Holštejnská 1; Martina 8, 9, 10´, 11´, 12 ´; Martinka R6; Maštalná 0, 1, 2, 3, 4/I, 4/II, 5, 8, 9; Medvedka 2, 6; Němcova 3; Peskö 2, 3, 5; Průchodnice I5; Skalka 2, 3, 4, 5; Srnčí 2, 3; Velká Ružínská 7, Železná 6).

**Parataxon 5+6**—*A*. *flavicollis* s.str.: 2009 molars (560 M1, 323 M2, 730 m1, 396 m2) in 111 community samples (missing only in 4 community samples: Hámorská 12; Martinka R2, R6; Peskö 8); The vast majority of the Late/Pleistocene Holocene record belongs to *A*. *flavicollis*, while the other species are clearly less abundant; FAD CZ: Late Vistulian/Preboreal (biozone A): Barová 11, Zkaměnělý zámek D8b, Martina 12´ and 13´, FAD SK: Late Vistulian/Preboreal—Maštalná 11 (biozone A).

**Parataxon 8**—*A*. *agrarius* s.str: 61 molars (15 M1, 6 M2, 25 m1, 15 m2) in 16 community samples (Červená skala II/3, II/4, II/5, 3, 4, 5, 6; Červeného mnícha 2; Hámorská 2; Holštejnská 1; Maštalná 0, 1, 2, 3; Medvedka 1; Skalka nad Čihovou 3); FAD CZ: Epiatlantic—Holštejnská 1 (biozone E); FAD SK: Late Boreal—Červená skala u Silice 5, 6 (biozone C2).

**Fig 8:**
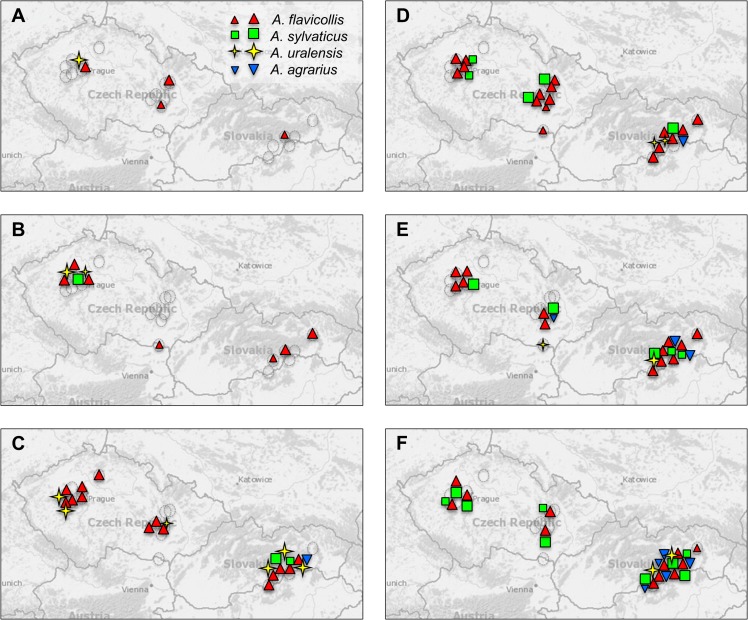
Graphical representation of postglacial recolonization by the genus *Apodemus* in Central Europe (A-Late Vistulian, B-Preboreal, C-Boreal, D-Atlantic, F-Late Holocene).

In regard to the appearance data on parataxa 1, 3, 5 and 8, i.e. the groups whose species identities can be considered as certain, the above-surveyed results show the following picture (comp. Fig 8). (i) All the Recent mid-European species appeared in the Holocene record. (ii) The genus is invariantly absent from the mid-European LGM record. (iii) The earliest records belong to *A*. *flavicollis*, the species clearly predominating in all fossil assemblages until the Late Holocene (Fig 7). (iv) *A*. *uralensis* also appeared regularly in the Late Vistulian and early Holocene record. In the early Holocene, it was obviously a regular element of mammalian communities far beyond its Recent distribution (e.g. Bohemian Karst). The middle and late Holocene records are restricted to SE Slovakia and S Moravia, i.e. the areas to which the present occurrence of the species in the region is restricted. (v) Against expectation, *A*. *sylvaticus* is represented by just a few items in the fossil record until the Late Holocene, though it had already appeared in Preboreal assemblages, first in the western part of the region, (vi) *A*. *agrarius* first appeared during the Boreal in the Pannonian region, with increased frequency during the post-Neolitic period. (vii) The Early Holocene ranges of *A*. *uralensis* and the post-Neolithic distribution of *A*. *agrarius* extended far beyond the margins of their Recent distribution in Central Europe.

## Discussion

With a combination of alternative determination techniques, we succeeded in disclosing species identity for the vast majority of the fossil material under study. Only a much smaller proportion of the material (8.3%), even smaller than expected, fell in the zones of between-species overlap (parataxa 2, 4), thus remaining virtually undetermined. This low percentage was also due to the absolute predominance (83.64%) of just one species, *Apodemus flavicollis*, in the fossil record–perhaps the most surprising output of the study. The absolute predominance of this species markedly contrasts with the picture suggested by most literary sources (comp. a survey by Kowalski [17]), according to which the vast majority of the Holocene and Late Pleistocene material of the genus belongs to *A*. *sylvaticus*. This holds entirely for rich materials from the Late Pleistocene–Holocene series from the Carpathian Basin in Hungary [41–43], surveyed recently by Pazonyi [44]. *A*. *sylvaticus* is reported to have occurred there continuously from 9 ky BP as a dominant element of Holocene communities (notwithstanding several records dated to the Late Vistulian, 26–10 ky BP), while no records of *A*. *flavicollis* or *A*. *uralensis* are reported. In regard to experience with the corresponding material from Czech and Slovak sites (including those neighbouring the Hungarian sites under discussion) formerly identified as *Apodemus (Sylvaemus)* sp. (comp. [18]), we expect that no exact determination procedure was undertaken, in fact, and hesitate to take the proposed identification in account. For corresponding reasons also the arguments validating a hypothesis on a glacial refugium of *A*.*sylvaticus* in the Carpathians [45] are considered as only weakly supported.

Storch [46–51], who in contrast to common approach paid particular care to the species identification of *Apodemus* from southern Germany, reports a picture which perfectly corresponds to the above surveyed results of the present study. The genus is absent during the LGM (Brillenhöhle), first appearing with *A*. *flavicollis* as a rare but regular element during the late Vistulian (Bölling–Alleröd, such as in Felsställe near Ehingen in Schwäbischen Alb Mts.) and in the earliest Holocene (Preboreal to Boreal), e.g. at several mesolithic sites such as Inzigkofen and the Fohlenhaus Cave in the upper Donau valley [48, 49]. *A*. *sylvaticus* appeared later and, until the Atlantic, was rather rare. A detailed survey of data from numerous archeological sites in Germany and Denmark provided by Fahlke [52] presents roughly the same picture. For the time window 15–12.5 ky BP, it reports two records from the Rhineland region and three from southern Bavaria (all *A*. *flavicollis*); for the time window 12–10.8 ky BP (Bölling-Alleröd), 4 sites in the western part of Germany and 6 in southern Germany; and for the time window 10–8 ky BP (Preboreal-Boreal), 2 in central Germany, 9 in southern Germany, 1 in Denmark and 1 in northern Germany. In the latter site (Piesede near Malchin), providing numerous assemblages from the Late Vistulian to the middle Holocene age, *Apodemus flavicollis* represents the third most common species, while *A*. *sylvaticus* is rather rare [26, 27].

In good agreement with the above surveyed data, our results also suggest that the postglacial expansion of the genus in Central Europe began with *A*. *flavicollis*, while *A*. *sylvaticus*, despite its early first appearances, became a common species in the late Holocene only. The difference in the expansion histories of these species seems to reflect the minute differences between them in habitat preference and the spatial organization of their populations. Neontological data from regions of syntopic occurrence (comp. e.g. [22, 53, 54]) show that *A*. *sylvaticus* prefers ecotone habitats with dense herbaceous vegetation and a low canopy, while *A*. *flavicollis* prefers to colonize mature deciduous, mixed, or coniferous forest with a high and dense canopy, and a spatially variegated ground surface with fallen trees, rocks etc. It can be expected that soon after the abrupt climatic amelioration terminating the LGM, the woodland habitat attained qualities more suitable for *A*. *flavicollis* than for *A*. *sylvaticus*, while those habitats promoting the spread of the latter species appeared only after Neolithic deforestation. In addition, the two species differ in the patterns of their population dynamics [55]: with increasing density, *A*. *flavicollis* tends to increase its spatial aggregation and abundance in the most suitable areas, while *A*. *sylvaticus* prefers randomly to disperse in order to reduce local densities. In other words, *A*. *flavicollis* seems to be much better disposed than *A*. *sylvaticus* to form a high abundance expansion front forcing a rapid range expansion. Its spread from its refugial range in the Balkans probably began synchronously with the first climatic amelioration terminating the LGM. The intensity of expansion was perhaps further accelerated by the Late Vistulian thermal anomaly in SE Europe, convincingly demonstrated by the palynological record [56]. The course of the range expansion in *A*. *flavicollis* must have been in general quite rapid, similarly as in *Pipistrellus pipistrellus*, whose early appearance in the mid-European fossil record represents another conspicuous feature of Late Pleistocene / Holocene faunal development [57].

Regarding the predominance of *A*. *flavicollis* in the Vistualian and Holocene fossil record of Central Europe, a question arises: which was therefore the actual history of *A*. *sylvaticus* during the present glacial cycle, and which is its true fossil record from that period?

*A*. *sylvaticus* (obviously properly identified) appears as a recedent element in the layers I-K in Sesselfelsgrotte, NE Bayern, a very rich series with reliable stratigraphy providing a detailed record of vertebrate faunal development throughout MIS2-4, 70–25 ky BP [58]. The layers I-K can be correlated to a warming event associated with the beginning of the MIS3 stage (56–50 ky). The numerous MIS3 fossil records of *A*. *sylvaticus* from southwestern France suggest its widespread, virtually continuous occurrence throughout the whole of the Vistulian glacial, with abundance peaks at 50, 35 and 25 ky BP [59], though the species might temporarily have disappeared during cold breaks (such as MIS 4 and 2), at least in some regions [60].

In contrast, in correspondingly rich continuous MIS3 sequences in the Western Carpathians in Slovakia (the Dzerava skala cave [61]) or in the Czestochowa upland in Poland (Komarowa cave, Krucza skala rock-shelter [62]), *Apodemus* is completely absent. The records of *Apodemus (“sylvaticus/flavicollis”)* in the LGM horizons of these sites (the Dzerava skala cave, the Polish sites Deszcowa Cave and Bisnik [61–63]) should be considered doubtful. In all these cases, the LGM layers are situated close to surface, and particularly for *Apodemus (Sylvaemus)* spp., postsedimentary penetration into surface sediments cannot be excluded. It is well known that wood mice use to colonize cave entrances quite frequently, particularly in winter, both for roosting and foraging (comp. e.g. [64]). Neglecting the records from Vistulian surface horizons, the first appearances of *Apodemus (“sylvaticus/flavicollis”)* at the respective Polish sites were between 11–12 ky BP [62], similarly to those demonstrated for *A*. *flavicollis* in our localities.

Numerous remains of “*Apodemus sylvaticus”* have been reported from a number of Late Vistulian assemblages from the Crimea (e.g. the aluvial deposits Sjuren 1, particularly the horizon with the Aurignacian artifacts, Sjuren 1/III; or in a deep layer of the Alimovskiy Naves sedimentary series, where later, during the Early Holocene, *A*. *flavicollis* also appeared in subsequently increasing abundances [65–67]). Simultaneous occurrences of *A*. *sylvaticus* and *A*. *flavicollis* have been reported from the Preboreal of Sjuren 2/II and several other sites, including the Late Paleolithic horizons in the Duruitor and Buzduzhen 1 localities in Moldavia [68–70].

Despite not examining the original materials, we hypothesize that the species identified as *A*. *sylvaticus* in the Ukrainian, Crimean and Moldovian Late Pleistocene record is, per analogiam to the mid-European record, in fact *A*. *uralensis*, a species somehow not taken into account by paleontologists. Only Popov [71], who carefully examined fossil records of *Apodemus* in Vistulian assemblages of the Temnata Cave, Balkan Mts., Bulgaria, identified *A*. *flavicollis* and *A*. *microps* (= *uralensis)* at that horizon. The hypothesis of the appearance of *A*. *uralensis* as the sole *Apodemus* species in MIS3 assemblages of Eastern Europe is further supported by the current distribution and habitat demands of that species–it is a typical steppe element. It is a common species in a broad variety of open ground habitats, capable of attaining high abundances in patches of sparse vegetation along temporal streams or marshlands, even under the extreme climatic conditions of the semi-desert habitats of Central Asia. It obviously survived the LGM stage at least in Eastern Europe (e.g. “*A*. *sylvaticus*” in Mezhirich site with 14C datum of 19 ky BP [67]). Yet, we will not further speculate which was the actual earlier history of *A*. *uralensis*, the taxon which is seemingly absent in the fossil record. We will only recall that in most of its current range it occurs in sympatry, and often even syntopically with another species with corresponding habitat requirements, namely *A*. *agrarius*, an inhabitant of open grounds with dense herbal vegetation and local patches of marshy floodplains [72, 73]. In Central Europe, *A*. *agrarius* reaches maximum densities in patches of riparian lowland forest, belts of willow stands, and shrub formations along marshy habitats, also preferred by *A*. *uralensis*. In the areas of syntopic occurrence, it shows a pronounced capacity to predominate over *A*. *uralensis* [73].

Robust molecular evidence on *A*. *agrarius* [8, 74, 75] revealing genetic homogeneity among Chinese, Russian and W Palearctic populations (<1% distance at cyt b) suggests that the W Palearctic population of *A*. *agrarius* originated from a rapid westward transcontinental expansion close to the Pleistocene / Holocene boundary. This prediction is in a good agreement with the fossil record both in Eastern and Central Europe. The species is obviously absent from the Late Pleistocene record in Ukraine and Crimea [67]; the first appearance in that region is from an Early Holocene assemblage from Devichiy skaly, Podolie region, W-Ukraine [76]. The first appearance in southern Slovakia comes from the late Boreal (ca 7000 BP), which roughly corresponds also to the FADs reported from Slovenia, viz. 9600–7800 BP (Podjamca cave) and 7800–6000 BP (Mala Triglavca cave) [77]. Böhme and Reichstein [78], discussing the origin of *A*. *agrarius* on the Danish islands of Lolland and Falster, assume that their colonization had to take place prior to a marine transgression at 7200 BP, isolating these islands. Nevertheless, there is still another fossil record of this species which deserves special attention. Aguilar et al. [79] report dental material corresponding closely to *A*. *agrarius* from a glacial assemblage in SW France (Bouzies-Q, Quercy) dated to 19.417–19.044 BP. This record, by far the most western extralimital offshoot of the species range, is quite exceptional, also in that it extensively predates all the western Palearctic fossil records of the species. If, despite certain differences, it in fact represents the extant species, then this would prove its westward expansion into Europe prior to the LGM. Such a possibility need not to be too surprising, of course. The driving factors which promoted the westward spread of Eastern Palearctic elements such as *A*. *agrarius* or *Micromys minutus* (comp. [11]) were undoubtedly closely related to the enormous energetic capacity of the mammoth steppe [80], the biome whose extension over most of the Holoarctic region culminated just during MIS3. Alternatively, with regard to certain morphometric differences [79], we should discuss the question of whether, eventually, the form recorded in the Quercy region does not represent a local endemic form of *Sylvaemus*, which during the Late Vistulian evolved the dental morphology convergent to *A*. *agrarius* (i.e. mandibular molars with an absence of cingular cusps, M2 without t3, and reduced t9 etc.). In this connection, we should mention another Middle to Late Pleistocene taxon which complicates our search for a clear view on the history of the genus in Central Europe: *Apodemus maastrichtiensis* van Kolfschoten, 1985. It was described from Saalian (MIS 7?) deposits from Maastricht-Belvedere and further reported from three sites of Eeemian age (MIS 5), two of MIS 9 (Kährlich, Schöningen 13) and from the Saalian site Wageningen-Fransche Kamp, which provided a larger sample enabling further biometrical analysis [58, 81]. *A*. *maastrichtiensis* corresponds in size to *A*. *uralensis*, from which it differs (according to van Kolfschoten [82]) by a reduced t3 cusp of M2 in most specimens and a smaller t9 of M1 and M2. It differs from other species of *Sylvaemus* also by the narrow and elongated t7 of M1 and by the steepness of the slopes of the cusps in its lower molars. The slopes of the cusps of m1 and m2 are more or less vertical and the angle formed by the chevrons is large. The anterior part of ml is isolated in most of the specimens and the antero-labial cusp of m2 is small. We examined the state of these characters in our material of *A*. *uralensis*, both recent and fossil (comp. states of non-metric variables F10,13,18,23 in S2 Table) and found partial agreement in several fossil specimens but not one which would respond to the diagnosis completely. At the moment, we feel unable to answer the question of possible relations between the Middle Pleistocene *A*. *maastrichtiensis* and the Late Pleistocene-Recent *A*. *uralensis*, nor to argue that the enlarged body size and further simplifications of the dental pattern characterizing *A*. *mastrichtiensis* could produce a phenotype convergent to *A*. *agrarius*, or to hypothesize on the extinction of that clade during the LGM or along the Pleistocene / Holocene transition. Nevertheless, such possibilities are undoubtedly worthy of further research interest. Last but not least, it reminds us that the history of the genus *Apodemus* in Europe is far from being completely understood and that further studies are urgently needed. Obviously, no fossil record is reported for the remaining extant species of the genus which exist in Central Europe: *A*. *alpicola*, a species restricted to high altitudes of the central and southwestern Alps, which demands rocky scree habitats with rich herbaceous vegetation and spatially variegated woodland cover with *Larix decidua* [22, 54, 83, 84]. Yet, we expect it never colonized the region covered by this report. It obviously represents a paleoendemite element of minute dispersal capacity, closely adapted to the specific conditions characterizing its Recent range, which most probably were locally available in the Alps throughout glacial times similarly as it was obviously the case of the High Tatra Mts. [85]. The remaing clade of European *Apodemus (A*.*mystacinus–epimelas)* which members can be easily distinguished by their larger size, obviously not appeared in Central Europe since the Early Pleistocene [1,17,18,43].

## Conclusions

Despite the various doubts and uncertainties, the above surveyed data enable us to draw–at least partially–a tentative biogeographic scenario conforming both to the outputs of molecular phylogeography and the fossil record (reconsidered as discussed above). It suggests that:

*Apodemus sylvaticus* survived in the western part of Central Europe (probably not in the Bohemian Massiff) until the Late MIS3; during the LGM, its range was probably restricted to Iberia and SW France. With its spread after the Late Vistulian climatic amelioration, it extended its range in Central Europe already in the Early Holocene, yet the species remained rare and supposedly not continuously distributed until the post-Neolithic period, most probably due to competition from *A*. *flavicollis*.

*Apodemus flavicollis* survived the LGM in the Balkans; its Late Pleistocene range expansion was very rapid. During the Late Pleistocene interstadials, it colonized a considerable part of its present range, including southern Germany, the Bohemian Massif, the Carpathians, Ukraine and Crimea. During the Preboreal, it increased its abundance continuously and since the beginning of the Boreal it has become the eudominant element in most regions.

*Apodemus uralensis* was most probably a constant inhabitant of the semi-open habitats of the mammoth steppe communities in eastern Europe, at least during MIS 3, though it never became a common species. It might even have survived the LGM in isolated local refugia in SE regions of Central Europe. In any case, together with *A*. *flavicollis*, it regularly colonized Central Europe already prior to the beginning of the Holocene. Then, it also colonized the central regions of the Bohemian Masiff quite far from its current range (except for a relic population of *A*. *uralensis cimrmani* in NW Bohemia). The restriction of its abundance and range is apparent by the end of the Boreal, probably due to the expansion of woodland habitats.

*Apodemus agrarius* colonized the western Palearctic (perhaps even including Central Europe) via the extensive westward expansion from its eastern Palearctic range probably during MIS 3. Nevertheless, its range was considerably restricted during the LGM. Then, its current European range seems to have been colonized by the late Boreal and particularly after post-Neolithic deforestation.

Regarding its contribution to the mammalian communities of Central Europe, it should be stressed that the genus had become a eudominant component of such communities already from the beginning of the Boreal (ca 9.3 ky BP) and that–against expectation—this role was, until the late Holocene, occupied by just a single species, namely *Apodemus flavicollis*.

## Supporting information

S1 Fig List of non-metric variables, span of their variation and scoring categories. (PDF)

S1 Table Additional information on source deposits of fossil samples. Including ^14^C data and primary references.
(PDF)

S2 Table List of basic statistics in the Recent samples of *Apodemus* spp. and extents of between-species overlaps in particular variables. (PDF)

S3 Table Testing effects of tooth wear and determination strategy upon state of particular metric and non-metric variables (one-way ANOVA). (PDF)

S4 Table Details of discrimination functions. (PDF)

S5 Table A detailed list of material and representation of particular parataxa in individual fossil samples. (PDF)
