# Supplementary material for: Late Pleistocene-Holocene paleobiogeography of the genus Apodemus in Central Europe
Source: PLoS One. 2017 Mar 10;12(3):e0173668. doi: 10.1371/journal.pone.0173668 (PMC5345881; doi:10.1371/journal.pone.0173668)

Supplementary file II: List of non-metric variables, span of their variation and scoring categories

A: degree of tooth wear

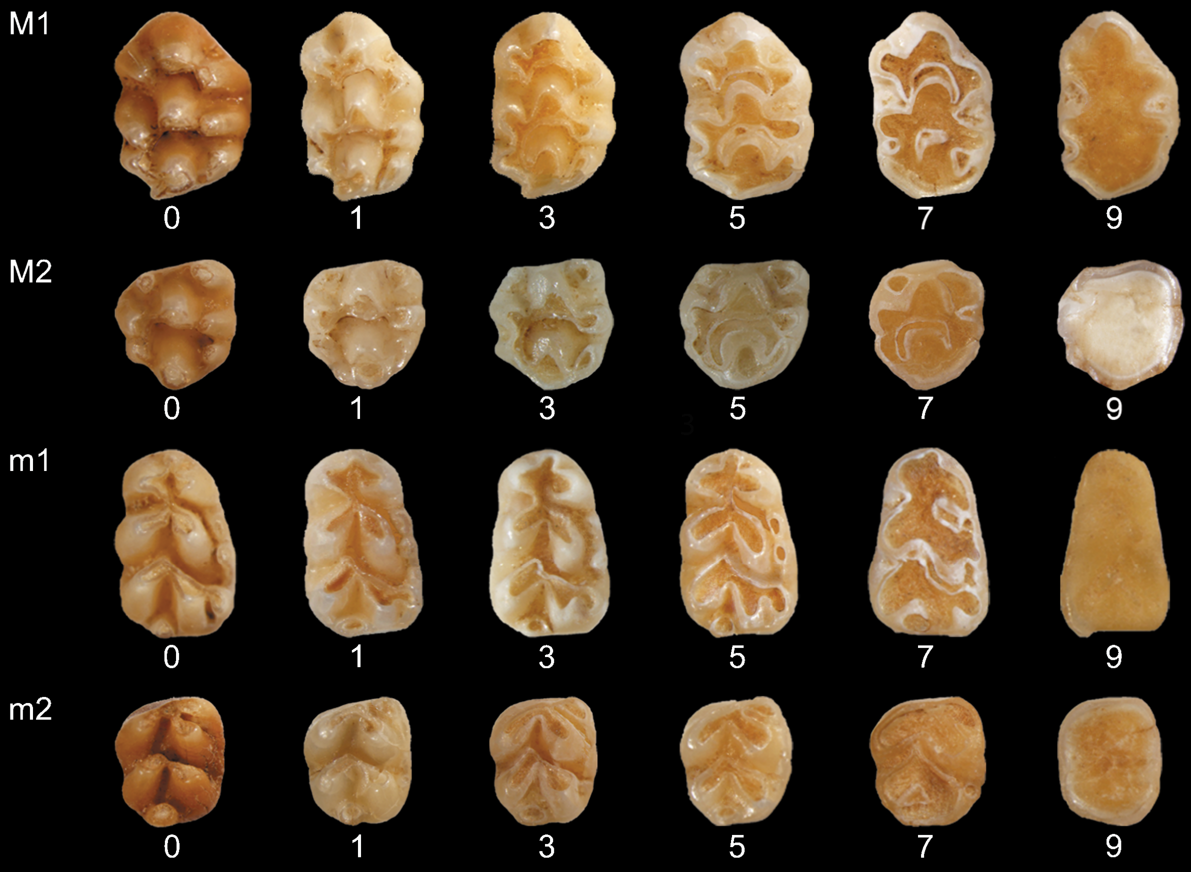

B: the non-metric variables of M1

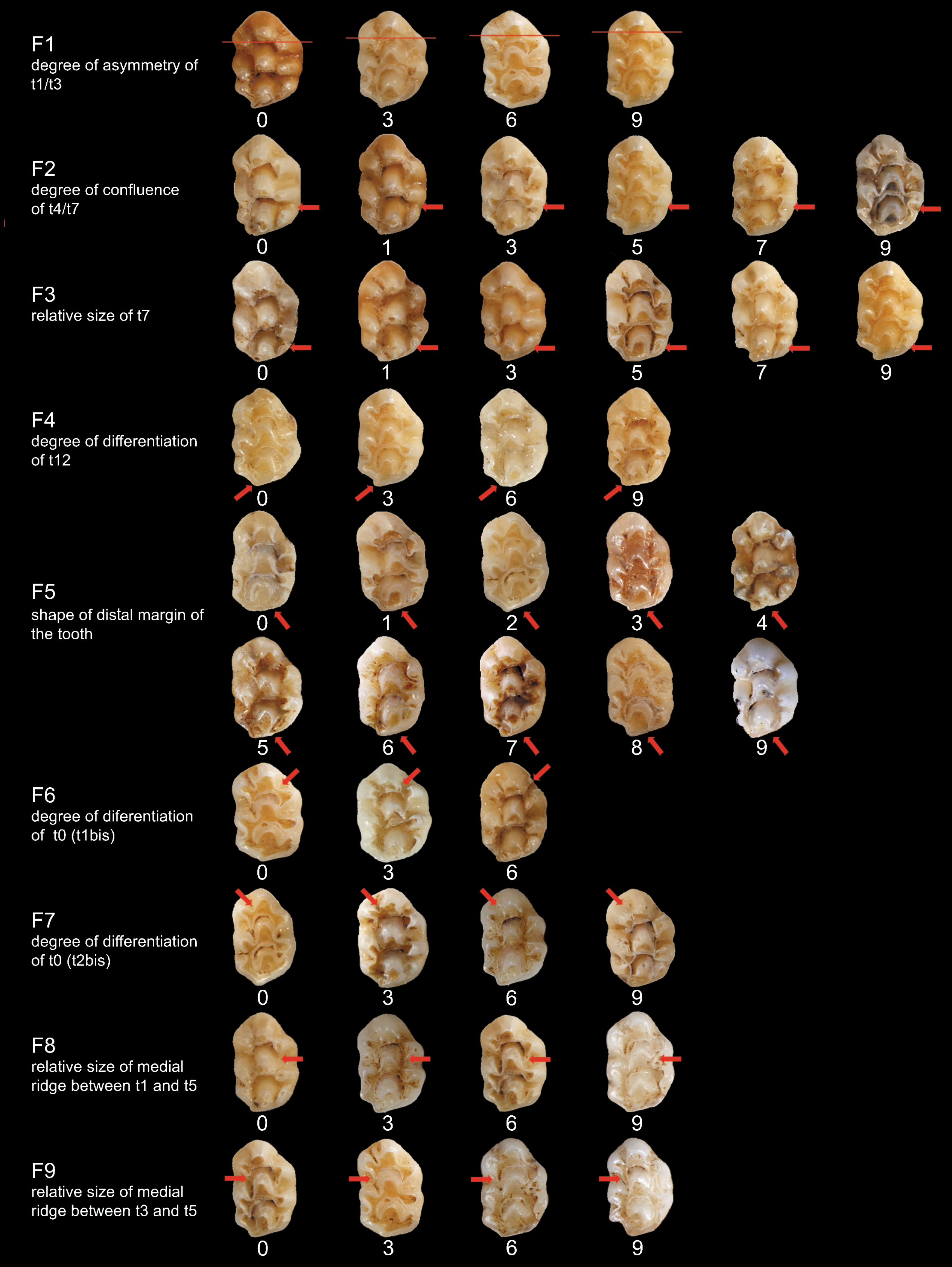

C: the non-metric variables of m1

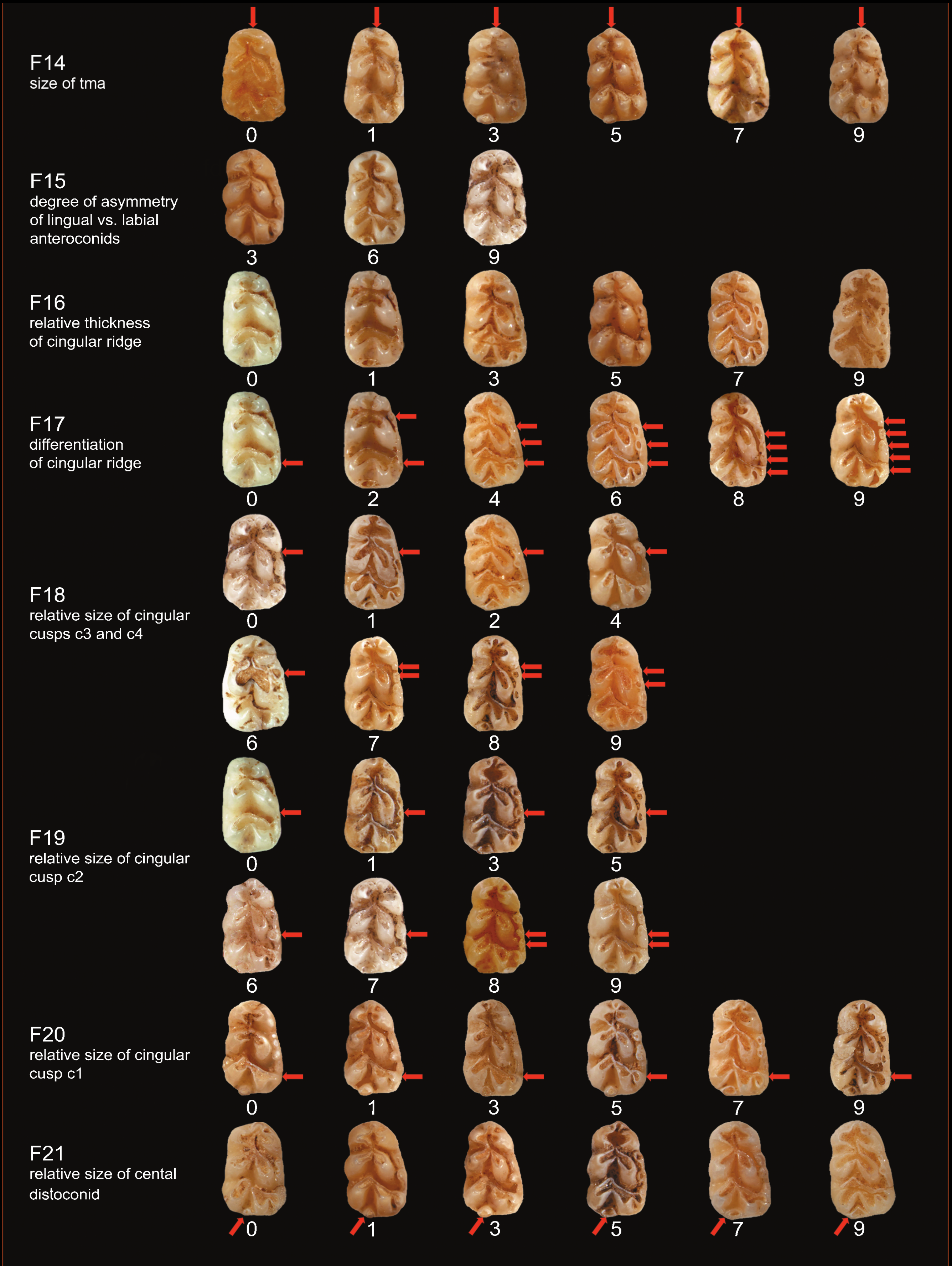

D: the non-metric variables of M2

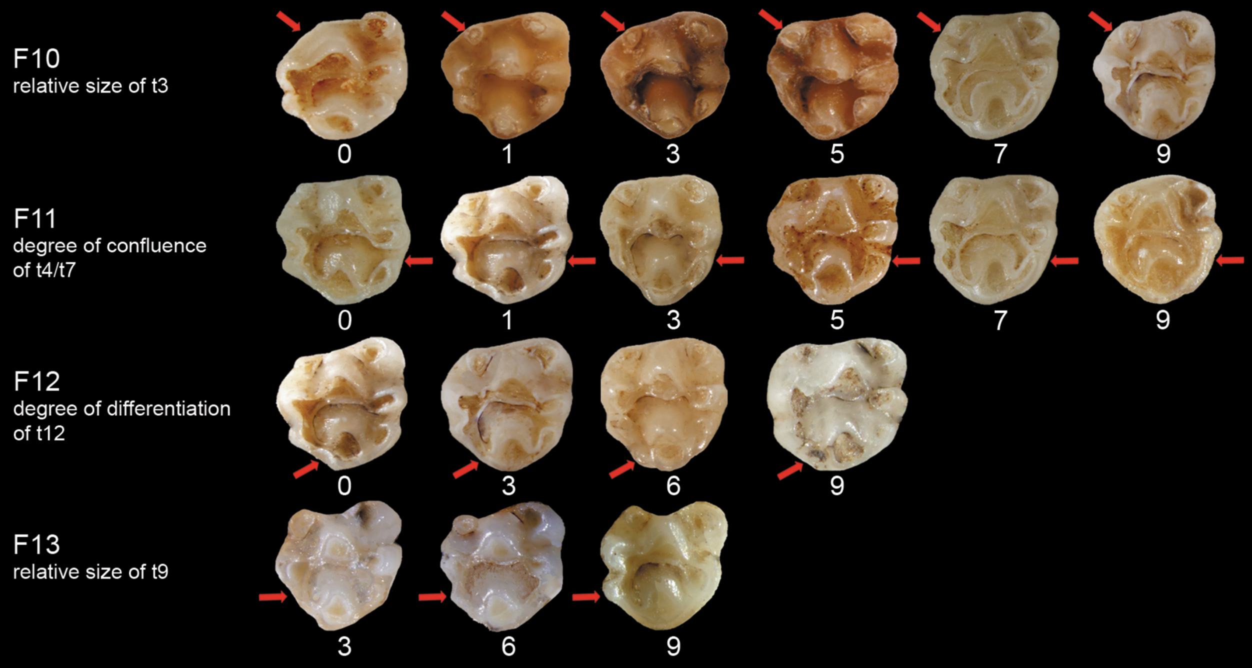

E: the non-metric variables of m2

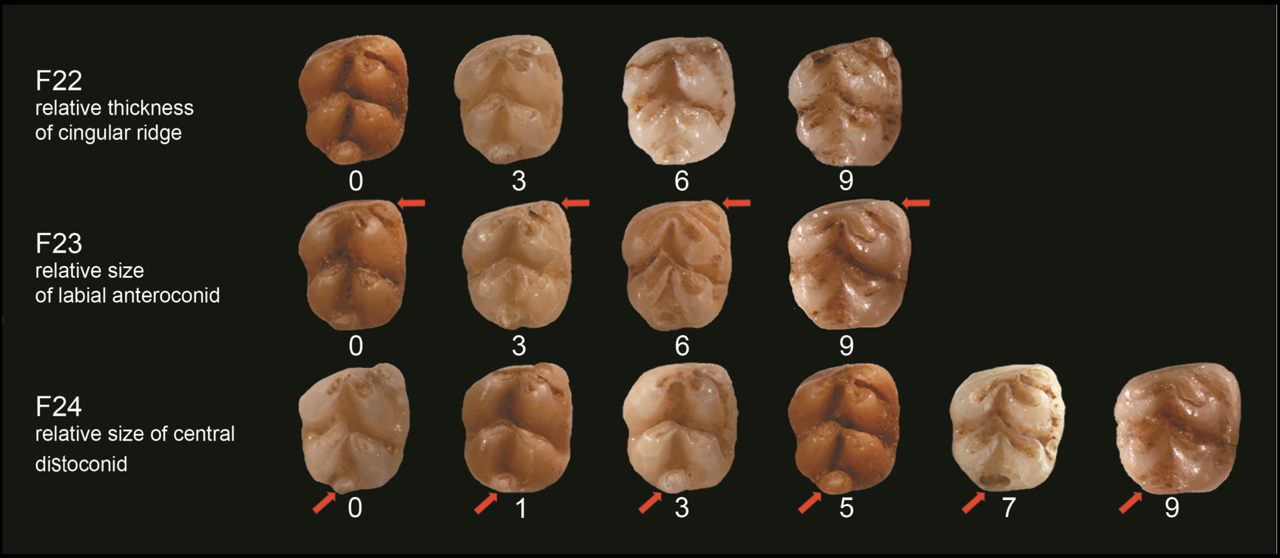

Supplement: S1 Fig — (PDF) [file pone.0173668.s001.pdf]
