# Supplementary material for: Late Pleistocene-Holocene paleobiogeography of the genus Apodemus in Central Europe
Source: PLoS One. 2017 Mar 10;12(3):e0173668. doi: 10.1371/journal.pone.0173668 (PMC5345881; doi:10.1371/journal.pone.0173668)
Supplement: S1 Table — Including 14C data and primary references. (PDF) [file pone.0173668.s002.pdf]

Supplementary file I: Additional information on source deposits of fossil samples (comp. Tab. 1 for geographic setting) – type of deposit; number of site in catalogues of fossil localities by Horáček and Ložek (1988) – H&L 1988 and Horáčková et al. (2014); available 14C data; and reference to primary literary survey (with complete faunal lists and detailed site descriptions).

			H&L 1988	H 2014	14C data (layer: min-max years cal BP)	detailed primary data
	BOHEMIA					
**AKSA**	Aksamitova brána (Tmaň, Bohemian Karst)	cave entrance	152	102	3: 3116-2940, 7 : 10328-10248	Matoušek et al. 1985
**BACI**	Bacín (Vinařice, Bohemian Karst)	cave entrance		61		Horáček 2002
**MART**	Martina (Tetín, Bohemian Karst)	cave entrance	44	46	A5: 5942-5249, A10: 10312-10249	Ložek and Horáček 2006
**SKAC**	Skalka nad Čihovou (Karlštejn, Bohemian Karst)	cave entrance	145	72	3: 2790-2543, 7: 7736-7642	Horáček and Ložek 1988/
**SKAM**	Skalice (Měňany, Bohemian Karst)	cave entrance		45	3: 6320-6066, 9: 13151-12806	Horáček et al. 2002
**ZELE**	Železná (Mramor, Bohemian Karst)	cave entrance		60		unpubl.
**PCER**	Pod Černou louží (Dřevčice, Česká Lípa)	abri		124	6: 8212-8588	Horáček 2007
	MORAVIA					
**BARO**	Barová (Habrůvka, Moravian Karst)	cave entrance	157	171	5: 6778-6544, 9: 7767-7663, 10b: 10260-9972	Horáček et al. 2002
**HOLS**	Holštejnská (Holštejn, Moravian Karst)	cave entrance	70	175	2: 5645-5606, 5: 14367-13909	Horáček and Ložek 1988
**NEMC**	Němcova (Suchdol, Moravian Karst)	cave entrance	156	169		Ložek and Horáček 1984
**SRNC**	Srnčí (Ostrov, Moravian Karst)	cave entrance	74	178	6b: 9937-9608	Horáček et al. 2002
**ZAZD**	Zazděná (Vavřinec, Moravian Karst)	cave entrance	72	176		Horáček and Jahelková 2005
**ZKAZ**	Zkamenělý zámek (Javoříčko, Olomouc distr.)	debris talus			9a: 16663-15815	Horáček at al. 2002
**PRUC**	Průchodnice (Ludmírov, Olomouc distr.)	abri		180		Horáček et al. 2002
**MARK**	Martinka (Horní Věstonice, Břeclav distr.)	debris talus	168	193		Horáček and Ložek 1990
	SLOVAKIA					
**MEDV**	Mara Medvedka cave (Divín, Lučenec)	cave entrance		260	3: 2727-2406, 7:8651-8506	Ložek et al. 1987
**PESK**	Peskö (Bretka, Rimavská Sobota distr.)	cave entrance	181	299	7b: 2198-2049	Ložek et al. 1989
**CESK**	Červená Skala (Silická Jablonica, Slovak Karst)	cave entrance	182	281		unpubl.
**HAMO**	Hámorská (Plešivec, Slovak Karst)	cave entrance		289	6: 4575-4350, 7: 9452-9179, 12: 14997-14192	Horáček and Ložek 1993
**CEMN**	Červeného mnícha (Jovice, Slovak Karst)	cave entrance		280		unpubl.
**MAST**	Maštalná (Brzotín, Slovak Karst)	cave entrance	180	285	4: 4461-4296, 6: 5309-5099, 9(2x): 8595-8406, 10b(2x): 10567-10272, 15: 16517-15226	Ložek and Horáček 1988
**RUZI**	Velká Ružínská jask. (Ružín, NE Slovakia)	cave entrance	125	254	3: 5360-5104, 8: 13541-13330	Ložek and Horáček 2007

Refences

Horáček I (2002) Fosilní obratlovci lokality Bacín [Fossil vertebrates of the Bacín site]. In: Svoboda J (ed) *Prehistorické jeskyně*: *katalogy*, *dokumenty*, *studie* [*Praehistoric Caves*: *Catalogues*, *Documents*, *Studies*], 292-293. Archeologický ústav AV ČR, Brno, Czech Republic (in Czech, with an abstract in English).

Horáček (2007) Holocene history of the mammalian fauna in the Northern Bohemian sandstone region (Czech Republic). In: Cílek V, Härtel H, Herben T, Jackson A, Rendel W (eds) *Sandstone Landscapes*, 265-272. Academia, Prague, Czech Republic.

Horáček I, Jahelková H (2005) History of the *Pipistrellus pipistrellus* group in Central Europe in light of its fossil record. *Acta Chiropterologica* 7: 189–204.

Horáček I, Ložek V (1988) Palaeozoology and the Mid-European Quaternary past: scope of the approach and selected results. *Rozpravy Československé Akademie Věd*, *Řada Matematických a Přírodních Věd* 98: 1–106.

Horáček I, Ložek V (1990) Biostratigrafický výzkum rozsedliny na Martince [Biostretigraphic research of the crevasse filling in the Martinka cliff]. *Československý Kras* 41: 83–99.

Horáček I, Ložek V (1993) Biostratigraphic investigation in the Hamorská cave (Slovak karst). In: Cílek V (ed): *Krasové sedimenty*. *Fosilní záznam klimatických oscilací a změn prostředí*. *Knihovna České speleologické společnosti*, *Svazek 21* [*Karst Sediments*. *The Fossil Record of Climate Oscillations and Environmental Changes*. *Library of the Czech Speleological Society*. *Volume 21*], 49-60. Nakladatelství Zlatý Kůň & Česká speleologická společnost, Praha, Czech Republic.

Horáček I, Ložek V, Svoboda J, Šajnerová A (2002) Přírodní prostředí a osídlení krasu v pozdním paleolitu a mezolitu [Environment and the settlement of karst in the Late Palaeolithic and Mesolithic]. In: Svoboda J (ed) *Prehistorické jeskyně*: *katalogy*, *dokumenty*, *studie* [*Praehistoric Caves*: *Catalogues*, *Documents*, *Studies*], 313-354. Archeologický ústav AV ČR Brno, Czech Republic (in Czech, with an abstract in English).

Horáčková J, Ložek V, Juřičková L (2015) List of malacologically treated Holocene sites with brief review of palaeomalacological research in the Czech and Slovak Republics. *Quaternary International* 357: 207–211.

Ložek V, Horáček I (1984) Nález fauny ve vchodu Němcovy jeskyně 1 v Moravském krasu [Fauna record in entrance of the Němcova jeskyně 1 cave in the Moravian Karst]. *Československý Kras* 35: 95–96 (in Czech).

Ložek V, Horáček I (1988) Vývoj přírody Plešivecké planiny v poledové době [Development of the Plešivecká Plateau in the post-glacial period]. *Ochrana Prírody*, *Výskumné Práce z Ochrany Prírody* 6A: 151–175 (in Czech, with summaries in English, German and Russian).

Ložek V, Horáček I (2006) Martina cave (Bohemian karst) – biostratigraphy of the entrance sediments. *Journal of Geological Sciences*, *Anthropozoic* 26: 61–71.

Ložek V, Horáček I (2007) Molluscan and vertebrate succesions from the Velká Ružínská cave (East Slovakia). In: Archäologische Gesellschaft in Thüringen e.V. (ed) *Terra Praehistorica*. *Festschrift für Klaus-Dieter Jäger zum 70*. *Geburtstag*, 224-232. Archäologische Gesellschaft in Thüringen e.V., Langenweißbach, Germany.

Ložek V, Horáček I, Gaál L (1987) Stratigrafický výskum jaskyne Mara Medvedia pri Divíne [Stratigraphical research of the Mara Medvedia cave at Devín]. In: Galvánek J (ed): *XXII*. *tábor ochrancov prírody*. *Prehľad odborných výsledkov* [*Twenty-second Camp of the Nature Protectors*. *Report of Results*], 17-26. Okresný národný výbor, Odbor kultúry Lučenec & ÚV SZOPK, Bratislava, Slovakia (in Slovak).

Ložek V, Gaál L, Holec P, Horáček I (1989) Stratigrafia a kvartérna fauna jaskyne Peskő v Rimavskej kotline [The stratigraphy and Quaternary fauna of the Peskő cave in the Rimavská Basin]. *Slovenský Kras* 27: 29–56 (in Slovak, with an abstract in German and summary in English).

Matoušek V, Beneš J, Ložek V, Horáček I (1985) Zpráva o 1. sezóně revizního archeologického výzkumu na Axamitově bráně [Report on the first revision archaeological research in the Axamitova Gate]. *Český Kras* 11: 7–35 (in Czech, with an abstract in English).
