# Supplementary material for: Late Pleistocene-Holocene paleobiogeography of the genus Apodemus in Central Europe
Source: PLoS One. 2017 Mar 10;12(3):e0173668. doi: 10.1371/journal.pone.0173668 (PMC5345881; doi:10.1371/journal.pone.0173668)
Supplement: S2 Table — (PDF) [file pone.0173668.s003.pdf]

**Supplementary file IIIa:** List of basic statistics in the Recent samples of *Apodemus* spp. and extents of between-species overlap in state of particular variables (those of suspect discrimation capacity, i.e. smaller than 30%, indicated by stars)

| sp: | *A.agrarius* | | | | | *A.flavicollis* | | | | | *A.sylvaticus* | | | | | *A.uralensis* | | | | | *Sylvaemus* overlap | <0.3 | *A.flav./A.sylv.* overlap | <0.3 | *A.sylv./A.ural.* overlap | <0.3 |
|---|---|---|---|---|---|---|---|---|---|---|---|---|---|---|---|---|---|---|---|---|---|---|---|---|---|---|
| | n | AVG | Min | Max | SD | n | AVG | Min | Max | SD | n | AVG | Min | Max | SD | n | AVG | Min | Max | SD | | | | | | |
| **M1U** | 22 | 1,958 | 1,78 | 2,06 | 0,074 | 75 | 2,053 | 1,90 | 2,22 | 0,075 | 62 | 1,820 | 1,69 | 1,95 | 0,064 | 87 | 1,642 | 1,42 | 1,77 | 0,090 | 0,1625 | ** | 0,0943 | *** | 0,1509 | ** |
| **M2U** | 22 | 0,730 | 0,64 | 0,82 | 0,052 | 74 | 0,693 | 0,53 | 0,80 | 0,050 | 63 | 0,601 | 0,48 | 0,72 | 0,052 | 87 | 0,561 | 0,42 | 0,70 | 0,060 | 1,0000 | | 0,5938 | | 0,7333 | |
| **M3U** | 22 | 0,608 | 0,54 | 0,69 | 0,034 | 74 | 0,623 | 0,55 | 0,68 | 0,031 | 61 | 0,566 | 0,50 | 0,65 | 0,030 | 87 | 0,499 | 0,42 | 0,57 | 0,037 | 0,6538 | | 0,5556 | | 0,3043 | |
| **M4U** | 23 | 1,168 | 1,04 | 1,24 | 0,043 | 75 | 1,350 | 1,21 | 1,49 | 0,052 | 63 | 1,225 | 1,13 | 1,33 | 0,049 | 87 | 1,110 | 1,00 | 1,19 | 0,045 | 0,3673 | | 0,3333 | | 0,1818 | ** |
| **M5U** | 22 | 1,068 | 0,99 | 1,17 | 0,045 | 75 | 1,263 | 1,14 | 1,41 | 0,058 | 63 | 1,129 | 1,02 | 1,25 | 0,053 | 87 | 0,993 | 0,87 | 1,13 | 0,046 | 0,4074 | | 0,2821 | * | 0,2895 | * |
| **M6U** | 22 | 0,789 | 0,56 | 0,97 | 0,095 | 74 | 0,859 | 0,72 | 1,16 | 0,088 | 63 | 0,821 | 0,64 | 1,01 | 0,101 | 87 | 0,660 | 0,51 | 0,82 | 0,068 | 0,7231 | | 0,5577 | | 0,3600 | |
| **M7U** | 22 | 0,843 | 0,68 | 1,01 | 0,106 | 74 | 0,911 | 0,74 | 1,13 | 0,077 | 63 | 0,861 | 0,70 | 1,21 | 0,095 | 87 | 0,753 | 0,61 | 0,89 | 0,070 | 1,0000 | | 1,0000 | | 0,3167 | |
| **M8U** | 22 | 0,470 | 0,42 | 0,52 | 0,032 | 74 | 0,468 | 0,39 | 0,52 | 0,029 | 63 | 0,410 | 0,35 | 0,49 | 0,031 | 87 | 0,385 | 0,32 | 0,46 | 0,030 | 1,0000 | | 0,5882 | | 0,6471 | |
| **M9U** | 20 | 0,400 | 0,36 | 0,49 | 0,036 | 74 | 0,458 | 0,38 | 0,59 | 0,038 | 60 | 0,420 | 0,35 | 0,51 | 0,038 | 87 | 0,386 | 0,30 | 0,48 | 0,034 | 0,8966 | | 0,5417 | | 0,6190 | |
| **M10U** | 23 | 0,295 | 0,24 | 0,34 | 0,030 | 74 | 0,323 | 0,22 | 0,43 | 0,045 | 63 | 0,286 | 0,19 | 0,39 | 0,050 | 87 | 0,251 | 0,16 | 0,37 | 0,038 | 1,0000 | | 0,7083 | | 0,7826 | |
| **M11U** | 22 | 0,297 | 0,23 | 0,38 | 0,038 | 74 | 0,317 | 0,24 | 0,41 | 0,040 | 60 | 0,271 | 0,17 | 0,36 | 0,045 | 87 | 0,231 | 0,13 | 0,35 | 0,043 | 1,0000 | | 0,5000 | | 0,7826 | |
| **M12U** | 22 | 0,738 | 0,63 | 0,84 | 0,054 | 74 | 0,698 | 0,55 | 0,79 | 0,049 | 63 | 0,620 | 0,52 | 0,74 | 0,053 | 87 | 0,571 | 0,43 | 0,70 | 0,059 | 1,0000 | | 0,7037 | | 0,5806 | |
| **M13U** | 22 | 0,730 | 0,64 | 0,82 | 0,059 | 74 | 0,709 | 0,55 | 0,81 | 0,053 | 63 | 0,628 | 0,52 | 0,76 | 0,049 | 87 | 0,562 | 0,43 | 0,68 | 0,057 | 0,9737 | | 0,7241 | | 0,4848 | |
| **M14U** | 22 | 0,643 | 0,55 | 0,80 | 0,048 | 74 | 0,752 | 0,65 | 0,84 | 0,042 | 60 | 0,662 | 0,58 | 0,74 | 0,035 | 87 | 0,575 | 0,51 | 0,66 | 0,035 | 0,5152 | | 0,3462 | | 0,3478 | |
| **M15U** | 22 | 0,663 | 0,55 | 0,81 | 0,046 | 74 | 0,780 | 0,66 | 0,87 | 0,040 | 60 | 0,679 | 0,59 | 0,76 | 0,038 | 87 | 0,597 | 0,51 | 0,69 | 0,035 | 0,5556 | | 0,3571 | | 0,4000 | |
| **M16U** | 22 | 0,674 | 0,58 | 0,74 | 0,040 | 74 | 0,702 | 0,60 | 0,78 | 0,034 | 60 | 0,638 | 0,54 | 0,72 | 0,033 | 87 | 0,573 | 0,49 | 0,67 | 0,038 | 0,8621 | | 0,5000 | | 0,5652 | |
| **M17U** | 22 | 0,692 | 0,60 | 0,74 | 0,038 | 74 | 0,712 | 0,61 | 0,82 | 0,036 | 60 | 0,631 | 0,46 | 0,77 | 0,051 | 87 | 0,575 | 0,48 | 0,68 | 0,038 | 1,0000 | | 0,4444 | | 0,7586 | |
| **M18U** | 22 | 1,121 | 1,00 | 1,25 | 0,067 | 74 | 1,379 | 1,20 | 1,50 | 0,063 | 62 | 1,188 | 1,05 | 1,32 | 0,066 | 87 | 1,121 | 0,92 | 1,17 | 0,053 | 0,4138 | | 0,2667 | * | 0,3000 | |
| **M19U** | 19 | 0,502 | 0,42 | 0,58 | 0,035 | 74 | 0,563 | 0,46 | 0,63 | 0,034 | 55 | 0,498 | 0,43 | 0,58 | 0,031 | 87 | 0,460 | 0,40 | 0,57 | 0,028 | 1,0000 | | 0,6000 | | 0,7778 | |
| **M20U** | 21 | 1,092 | 1,05 | 1,16 | 0,035 | 72 | 1,285 | 1,16 | 1,37 | 0,046 | 56 | 1,163 | 1,08 | 1,28 | 0,044 | 87 | 1,068 | 0,94 | 1,17 | 0,041 | 0,4884 | | 0,4138 | | 0,2647 | * |
| **M21U** | 20 | 0,923 | 0,84 | 0,99 | 0,046 | 71 | 1,136 | 1,02 | 1,25 | 0,052 | 57 | 0,992 | 0,84 | 1,12 | 0,049 | 87 | 0,906 | 0,73 | 1,05 | 0,054 | 0,5962 | | 0,2439 | * | 0,5385 | |
| **M22U** | 19 | 0,852 | 0,71 | 0,99 | 0,072 | 72 | 0,964 | 0,83 | 1,11 | 0,059 | 54 | 0,878 | 0,71 | 0,97 | 0,069 | 87 | 0,791 | 0,64 | 0,98 | 0,067 | 0,8723 | | 0,3500 | | 0,8182 | |
| **M23U** | 19 | 0,482 | 0,40 | 0,55 | 0,036 | 74 | 0,471 | 0,40 | 0,56 | 0,037 | 52 | 0,428 | 0,36 | 0,55 | 0,041 | 86 | 0,391 | 0,31 | 0,47 | 0,034 | 1,0000 | | 0,7500 | | 0,4583 | |
| **M24U** | 19 | 0,389 | 0,34 | 0,46 | 0,030 | 74 | 0,463 | 0,40 | 0,54 | 0,036 | 54 | 0,436 | 0,36 | 0,50 | 0,033 | 86 | 0,397 | 0,30 | 0,49 | 0,037 | 0,9583 | | 0,5556 | | 0,6500 | |
| **M25U** | 19 | 0,382 | 0,29 | 0,47 | 0,053 | 74 | 0,506 | 0,38 | 0,64 | 0,048 | 55 | 0,427 | 0,31 | 0,53 | 0,052 | 87 | 0,405 | 0,32 | 0,49 | 0,039 | 1,0000 | | 0,4545 | | 0,8571 | |
| **M26U** | 19 | 0,282 | 0,21 | 0,41 | 0,046 | 74 | 0,371 | 0,27 | 0,50 | 0,051 | 55 | 0,304 | 0,20 | 0,41 | 0,048 | 87 | 0,252 | 0,15 | 0,37 | 0,038 | 0,8857 | | 0,4667 | | 0,6538 | |
| **M27U** | 19 | 0,514 | 0,43 | 0,61 | 0,043 | 74 | 0,547 | 0,45 | 0,61 | 0,034 | 55 | 0,508 | 0,43 | 0,59 | 0,035 | 87 | 0,462 | 0,40 | 0,62 | 0,034 | 1,0000 | | 0,7778 | | 1,0000 | |
| **M28U** | 19 | 0,521 | 0,45 | 0,58 | 0,040 | 74 | 0,580 | 0,50 | 0,69 | 0,036 | 55 | 0,521 | 0,44 | 0,61 | 0,034 | 87 | 0,482 | 0,40 | 0,64 | 0,034 | 1,0000 | | 0,4400 | | 0,9524 | |
| **M29U** | 19 | 0,546 | 0,48 | 0,61 | 0,035 | 74 | 0,763 | 0,66 | 0,86 | 0,043 | 55 | 0,658 | 0,56 | 0,73 | 0,037 | 87 | 0,585 | 0,46 | 0,66 | 0,038 | 0,4250 | | 0,2333 | * | 0,3704 | |
| **M30U** | 19 | 0,549 | 0,46 | 0,61 | 0,040 | 74 | 0,758 | 0,65 | 0,86 | 0,046 | 55 | 0,641 | 0,55 | 0,71 | 0,037 | 87 | 0,588 | 0,48 | 0,66 | 0,035 | 0,4474 | | 0,1935 | ** | 0,4783 | |
| **m1L** | 23 | 1,653 | 1,53 | 1,77 | 0,064 | 74 | 1,888 | 1,75 | 2,05 | 0,067 | 63 | 1,677 | 1,52 | 1,81 | 0,071 | 83 | 1,514 | 1,39 | 1,64 | 0,061 | 0,2727 | * | 0,1132 | ** | 0,2857 | * |
| **m2L** | 18 | 0,377 | 0,26 | 0,52 | 0,068 | 74 | 0,346 | 0,20 | 0,59 | 0,084 | 60 | 0,379 | 0,20 | 0,55 | 0,087 | 83 | 0,331 | 0,19 | 0,50 | 0,060 | 1,0000 | | 0,8974 | | 0,8333 | |
| **m3L** | 19 | 0,708 | 0,53 | 0,90 | 0,092 | 74 | 0,720 | 0,55 | 0,97 | 0,088 | 62 | 0,720 | 0,51 | 0,94 | 0,092 | 83 | 0,620 | 0,48 | 0,77 | 0,061 | 1,0000 | | 0,8478 | | 0,5652 | |
| **m4L** | 18 | 1,256 | 1,07 | 1,36 | 0,080 | 74 | 1,365 | 1,22 | 1,56 | 0,089 | 59 | 1,263 | 1,06 | 1,45 | 0,094 | 83 | 1,122 | 0,96 | 1,27 | 0,070 | 0,7333 | | 0,4600 | | 0,4286 | |
| **m5L** | 20 | 0,961 | 0,84 | 1,02 | 0,044 | 74 | 1,197 | 1,11 | 1,29 | 0,046 | 59 | 1,078 | 0,94 | 1,23 | 0,057 | 83 | 1,004 | 0,93 | 1,10 | 0,045 | 0,7778 | | 0,3429 | | 0,5333 | |
| **m6L** | 23 | 0,714 | 0,63 | 0,79 | 0,051 | 74 | 0,969 | 0,87 | 1,12 | 0,049 | 63 | 0,880 | 0,76 | 0,98 | 0,050 | 83 | 0,791 | 0,71 | 0,92 | 0,052 | 0,6585 | | 0,3056 | | 0,5926 | |
| **m7L** | 18 | 0,418 | 0,35 | 0,52 | 0,052 | 74 | 0,520 | 0,32 | 0,82 | 0,082 | 61 | 0,496 | 0,36 | 0,67 | 0,065 | 83 | 0,401 | 0,27 | 0,62 | 0,071 | 1,0000 | | 0,7609 | | 0,6500 | |
| **m8L** | 18 | 0,558 | 0,42 | 0,68 | 0,075 | 74 | 0,585 | 0,46 | 0,80 | 0,057 | 62 | 0,546 | 0,38 | 0,85 | 0,079 | 83 | 0,473 | 0,32 | 0,65 | 0,067 | 1,0000 | | 0,9286 | | 0,5094 | |
| **m9L** | 18 | 0,560 | 0,44 | 0,69 | 0,072 | 74 | 0,604 | 0,47 | 0,76 | 0,057 | 59 | 0,558 | 0,45 | 0,67 | 0,062 | 83 | 0,490 | 0,37 | 0,63 | 0,055 | 0,9744 | | 0,6452 | | 0,6000 | |
| **m10L** | 21 | 0,010 | 0,00 | 0,08 | 0,025 | 74 | 0,210 | 0,15 | 0,27 | 0,029 | 59 | 0,194 | 0,07 | 0,29 | 0,044 | 83 | 0,168 | 0,11 | 0,23 | 0,028 | 1,0000 | | 0,7000 | | 0,8889 | |
| **m11L** | 18 | 0,214 | 0,18 | 0,25 | 0,019 | 74 | 0,272 | 0,17 | 0,37 | 0,033 | 57 | 0,254 | 0,15 | 0,31 | 0,034 | 83 | 0,226 | 0,15 | 0,28 | 0,028 | 1,0000 | | 0,6364 | | 0,8125 | |
| **m12L** | 18 | 0,484 | 0,34 | 0,61 | 0,066 | 74 | 0,680 | 0,49 | 0,83 | 0,071 | 60 | 0,565 | 0,39 | 0,75 | 0,090 | 83 | 0,488 | 0,40 | 0,65 | 0,059 | 1,0000 | | 0,5909 | | 0,7429 | |
| **m13L** | 18 | 0,506 | 0,33 | 0,62 | 0,080 | 74 | 0,597 | 0,39 | 0,76 | 0,061 | 60 | 0,515 | 0,36 | 0,69 | 0,068 | 83 | 0,407 | 0,29 | 0,53 | 0,053 | 1,0000 | | 0,7500 | | 0,4250 | |
| **m14L** | 18 | 0,748 | 0,65 | 0,83 | 0,048 | 74 | 0,940 | 0,76 | 1,15 | 0,064 | 59 | 0,795 | 0,59 | 0,93 | 0,073 | 83 | 0,727 | 0,63 | 0,83 | 0,044 | 0,7885 | | 0,3036 | | 0,8000 | |
| **m15L** | 18 | 0,681 | 0,59 | 0,76 | 0,045 | 74 | 0,824 | 0,67 | 0,94 | 0,056 | 59 | 0,688 | 0,52 | 0,85 | 0,066 | 83 | 0,602 | 0,51 | 0,70 | 0,039 | 0,8372 | | 0,4286 | | 0,5294 | |
| **m16L** | 23 | 1,098 | 0,98 | 1,21 | 0,059 | 74 | 1,326 | 1,19 | 1,43 | 0,054 | 63 | 1,155 | 1,02 | 1,28 | 0,059 | 80 | 1,065 | 0,96 | 1,16 | 0,046 | 0,4894 | | 0,2195 | * | 0,4375 | |
| **m17L** | 20 | 0,245 | 0,13 | 0,48 | 0,085 | 74 | 0,177 | 0,11 | 0,39 | 0,055 | 62 | 0,207 | 0,09 | 0,50 | 0,091 | 80 | 0,161 | 0,05 | 0,37 | 0,071 | 1,0000 | | 1,0000 | | 0,6222 | |
| **m18L** | 19 | 0,692 | 0,59 | 0,78 | 0,053 | 74 | 0,719 | 0,59 | 0,95 | 0,074 | 57 | 0,667 | 0,54 | 0,86 | 0,070 | 80 | 0,615 | 0,52 | 0,78 | 0,058 | 1,0000 | | 0,6585 | | 0,7059 | |
| **m19L** | 23 | 0,954 | 0,72 | 1,05 | 0,076 | 74 | 1,192 | 1,06 | 1,28 | 0,044 | 60 | 1,045 | 0,92 | 1,16 | 0,052 | 80 | 0,984 | 0,89 | 1,07 | 0,043 | 0,6410 | | 0,2778 | * | 0,5556 | |
| **m20L** | 23 | 0,879 | 0,51 | 0,98 | 0,119 | 74 | 1,110 | 0,99 | 1,22 | 0,050 | 62 | 0,955 | 0,81 | 1,09 | 0,058 | 80 | 0,893 | 0,81 | 1,00 | 0,048 | 0,7073 | | 0,2439 | * | 0,6786 | |
| **m21L** | 20 | 0,626 | 0,47 | 0,78 | 0,082 | 74 | 0,658 | 0,47 | 0,92 | 0,073 | 60 | 0,609 | 0,46 | 0,75 | 0,075 | 80 | 0,557 | 0,41 | 0,75 | 0,077 | 1,0000 | | 0,6087 | | 0,8529 | |
| **m22L** | 17 | 0,567 | 0,45 | 0,65 | 0,060 | 74 | 0,674 | 0,56 | 0,78 | 0,041 | 57 | 0,595 | 0,46 | 0,71 | 0,056 | 80 | 0,532 | 0,41 | 0,66 | 0,057 | 0,9459 | | 0,4688 | | 0,6667 | |
| **m23L** | 19 | 0,235 | 0,15 | 0,31 | 0,052 | 74 | 0,373 | 0,28 | 0,50 | 0,048 | 60 | 0,318 | 0,20 | 0,39 | 0,041 | 80 | 0,275 | 0,16 | 0,37 | 0,049 | 0,8235 | | 0,3667 | | 0,7391 | |
| **m24L** | 20 | 0,412 | 0,30 | 0,62 | 0,077 | 74 | 0,470 | 0,00 | 0,74 | 0,087 | 61 | 0,448 | 0,32 | 0,69 | 0,077 | 80 | 0,394 | 0,25 | 0,53 | 0,064 | 1,0000 | | 1,0000 | | 0,4773 | |
| **m25L** | 20 | 0,438 | 0,29 | 0,63 | 0,082 | 74 | 0,445 | 0,32 | 0,64 | 0,064 | 61 | 0,436 | 0,23 | 0,62 | 0,077 | 80 | 0,355 | 0,21 | 0,51 | 0,066 | 1,0000 | | 0,7317 | | 0,6829 | |
| **m26L** | 18 | 0,646 | 0,57 | 0,72 | 0,035 | 74 | 0,797 | 0,70 | 0,96 | 0,054 | 56 | 0,687 | 0,59 | 0,86 | 0,048 | 80 | 0,646 | 0,55 | 0,74 | 0,042 | 0,7561 | | 0,4324 | | 0,4839 | |
| **m27L** | 17 | 0,592 | 0,53 | 0,64 | 0,030 | 74 | 0,746 | 0,64 | 0,91 | 0,056 | 56 | 0,642 | 0,46 | 0,80 | 0,066 | 80 | 0,571 | 0,46 | 0,66 | 0,045 | 0,8000 | | 0,3556 | | 0,5882 | |
| **M2U/M1U** | 22 | 0,373 | 0,33 | 0,43 | 0,025 | 74 | 0,337 | 0,28 | 0,38 | 0,019 | 62 | 0,330 | 0,28 | 0,38 | 0,023 | 87 | 0,341 | 0,29 | 0,40 | 0,022 | 1,0000 | | 0,9932 | | 1,0000 | |
| **M14U/M15U** | 22 | 0,970 | 0,91 | 1,01 | 0,026 | 74 | 0,965 | 0,85 | 1,09 | 0,043 | 60 | 0,976 | 0,88 | 1,10 | 0,039 | 87 | 0,964 | 0,88 | 1,05 | 0,036 | 1,0000 | | 1,0000 | | 0,7732 | |
| **m3L/m1L** | 19 | 0,428 | 0,33 | 0,56 | 0,058 | 74 | 0,382 | 0,31 | 0,52 | 0,047 | 62 | 0,430 | 0,33 | 0,60 | 0,061 | 83 | 0,409 | 0,33 | 0,52 | 0,037 | 1,0000 | | 1,0000 | | 0,6986 | |
| **m6L/m5L** | 20 | 0,734 | 0,63 | 0,85 | 0,054 | 74 | 0,810 | 0,74 | 0,88 | 0,027 | 59 | 0,819 | 0,74 | 0,88 | 0,032 | 83 | 0,788 | 0,71 | 0,87 | 0,034 | 1,0000 | | 1,0000 | | 0,7285 | |
| **F1** | 22 | 5,864 | 3 | 9 | 2,167 | 75 | 2,480 | 0 | 9 | 2,580 | 63 | 3,714 | 0 | 9 | 2,624 | 87 | 3,655 | 0 | 9 | 2,723 | 1,0000 | | 1,0000 | | 1,0000 | |
| **F2** | 19 | 6,368 | 3 | 9 | 1,832 | 74 | 5,595 | 3 | 9 | 1,461 | 63 | 6,111 | 2 | 9 | 2,009 | 87 | 5,529 | 2 | 9 | 1,516 | 1,0000 | | 0,8571 | | 1,0000 | |
| **F3** | 18 | 6,722 | 4 | 8 | 1,227 | 74 | 6,351 | 4 | 9 | 1,329 | 54 | 5,519 | 0 | 9 | 2,152 | 87 | 5,310 | 1 | 9 | 1,558 | 1,0000 | | 0,5556 | | 1,0000 | |
| **F4** | 23 | 0,783 | 0 | 3 | 1,347 | 72 | 5,833 | 0 | 9 | 3,099 | 60 | 2,750 | 0 | 9 | 3,187 | 87 | 4,103 | 0 | 9 | 3,547 | 1,0000 | | 1,0000 | | 1,0000 | |
| **F5** | 23 | 2,087 | 0 | 3 | 0,848 | 73 | 4,630 | 0 | 9 | 2,306 | 62 | 1,887 | 0 | 8 | 1,610 | 87 | 2,851 | 0 | 9 | 1,926 | 1,0000 | | 0,8889 | | 1,0000 | |
| **F6** | 23 | 0,000 | 0 | 0 | 0,000 | 75 | 0,200 | 0 | 3 | 0,753 | 63 | 0,429 | 0 | 6 | 1,187 | 87 | 0,345 | 0 | 6 | 1,247 | 1,0000 | | 1,0000 | | 1,0000 | |
| **F7** | 23 | 0,000 | 0 | 0 | 0,000 | 75 | 2,880 | 0 | 9 | 2,676 | 63 | 2,905 | 0 | 9 | 2,900 | 87 | 3,759 | 0 | 9 | 2,791 | 1,0000 | | 1,0000 | | 1,0000 | |
| **F8** | 21 | 1,857 | 0 | 6 | 2,220 | 74 | 3,446 | 0 | 9 | 2,516 | 63 | 3,143 | 0 | 9 | 2,770 | 87 | 0,828 | 0 | 6 | 1,564 | 1,0000 | | 1,0000 | | 0,6667 | |
| **F9** | 21 | 2,143 | 0 | 6 | 1,931 | 74 | 3,000 | 0 | 6 | 1,570 | 63 | 3,286 | 0 | 9 | 2,331 | 87 | 0,586 | 0 | 3 | 1,196 | 1,0000 | | 1,0000 | | 0,3333 | |
| **F10** | 23 | 0,000 | 0 | 0 | 0,000 | 74 | 4,581 | 1 | 8 | 1,471 | 54 | 4,074 | 1 | 9 | 1,810 | 87 | 4,218 | 1 | 9 | 1,991 | 1,0000 | | 1,0000 | | 1,0000 | |
| **F11** | 19 | 5,789 | 3 | 9 | 1,584 | 73 | 5,616 | 3 | 9 | 1,411 | 54 | 5,870 | 0 | 9 | 2,266 | 86 | 5,907 | 3 | 9 | 1,334 | 1,0000 | | 0,6667 | | 1,0000 | |
| **F12** | 23 | 0,261 | 0 | 3 | 0,864 | 73 | 3,082 | 0 | 9 | 2,783 | 56 | 2,036 | 0 | 9 | 2,149 | 87 | 3,345 | 0 | 9 | 2,909 | 1,0000 | | 1,0000 | | 1,0000 | |
| **F13** | 20 | 7,500 | 6 | 9 | 1,539 | 72 | 5,458 | 3 | 9 | 1,906 | 54 | 7,944 | 6 | 9 | 1,446 | 87 | 6,966 | 3 | 9 | 1,742 | 1,0000 | | 1,0000 | | 0,5000 | |
| **F14** | 18 | 6,833 | 4 | 8 | 1,150 | 74 | 5,027 | 0 | 9 | 1,894 | 60 | 5,250 | 1 | 9 | 1,733 | 83 | 4,349 | 0 | 9 | 2,211 | 1,0000 | | 1,0000 | | 0,8889 | |
| **F15** | 19 | 5,684 | 3 | 9 | 1,376 | 74 | 4,257 | 3 | 9 | 1,571 | 62 | 4,742 | 3 | 9 | 1,924 | 83 | 4,590 | 3 | 9 | 1,774 | 1,0000 | | 1,0000 | | 1,0000 | |
| **F16** | 18 | 1,389 | 1 | 3 | 0,608 | 74 | 5,203 | 2 | 8 | 1,182 | 58 | 4,793 | 2 | 8 | 1,295 | 83 | 5,422 | 2 | 8 | 1,335 | 1,0000 | | 1,0000 | | 1,0000 | |
| **F17** | 18 | 2,778 | 1 | 4 | 0,732 | 73 | 5,658 | 0 | 9 | 1,938 | 58 | 4,621 | 2 | 9 | 1,653 | 83 | 5,422 | 2 | 9 | 2,193 | 1,0000 | | 1,0000 | | 1,0000 | |
| **F18** | 18 | 2,722 | 1 | 5 | 1,447 | 73 | 4,438 | 1 | 9 | 2,048 | 59 | 3,780 | 0 | 8 | 2,190 | 83 | 2,976 | 0 | 7 | 2,353 | 1,0000 | | 0,7778 | | 0,8750 | |
| **F19** | 18 | 0,111 | 0 | 1 | 0,323 | 73 | 5,123 | 1 | 9 | 2,303 | 58 | 5,000 | 2 | 9 | 1,686 | 83 | 5,530 | 1 | 9 | 2,652 | 1,0000 | | 1,0000 | | 0,8750 | |
| **F20** | 18 | 4,333 | 2 | 6 | 1,237 | 74 | 5,392 | 3 | 9 | 1,322 | 58 | 4,845 | 0 | 8 | 1,519 | 83 | 5,386 | 2 | 9 | 1,387 | 1,0000 | | 0,5556 | | 1,0000 | |
| **F21** | 17 | 6,235 | 5 | 8 | 1,033 | 74 | 5,432 | 2 | 8 | 1,405 | 57 | 5,421 | 2 | 9 | 1,841 | 83 | 5,265 | 2 | 9 | 1,639 | 1,0000 | | 1,0000 | | 1,0000 | |
| **F22** | 18 | 1,667 | 0 | 6 | 1,414 | 74 | 4,865 | 0 | 9 | 2,418 | 60 | 2,450 | 0 | 6 | 1,702 | 80 | 4,200 | 0 | 9 | 2,415 | 1,0000 | | 0,6667 | | 1,0000 | |
| **F23** | 18 | 0,500 | 0 | 3 | 1,150 | 74 | 4,603 | 0 | 9 | 2,184 | 61 | 3,738 | 0 | 9 | 2,664 | 80 | 3,300 | 0 | 9 | 2,597 | 1,0000 | | 1,0000 | | 1,0000 | |
| **F24** | 18 | 5,833 | 1 | 8 | 1,757 | 74 | 6,351 | 0 | 9 | 1,675 | 56 | 5,679 | 3 | 9 | 1,707 | 80 | 4,863 | 1 | 8 | 1,473 | 1,0000 | | 1,0000 | | 0,6250 | |
| **SURM1** | 22 | 2,286 | 1,85 | 2,44 | 0,146 | 75 | 2,775 | 2,34 | 3,31 | 0,193 | 62 | 2,233 | 1,94 | 2,52 | 0,143 | 87 | 1,825 | 1,43 | 2,11 | 0,160 | 0,1881 | ** | 0,1351 | ** | 0,1541 | ** |
| **SURM2** | 21 | 1,227 | 1,10 | 1,36 | 0,084 | 72 | 1,776 | 1,39 | 2,03 | 0,130 | 56 | 1,392 | 1,17 | 1,65 | 0,108 | 87 | 1,198 | 0,93 | 1,35 | 0,092 | 0,3991 | | 0,3010 | | 0,2482 | * |
| **SURm1** | 20 | 1,586 | 1,31 | 1,81 | 0,117 | 74 | 2,261 | 1,96 | 2,63 | 0,153 | 59 | 1,818 | 1,47 | 2,08 | 0,149 | 83 | 1,522 | 1,31 | 1,74 | 0,119 | 0,2913 | * | 0,0978 | *** | 0,3523 | |
| **SURm2** | 23 | 1,050 | 0,71 | 1,23 | 0,124 | 74 | 1,582 | 1,26 | 1,79 | 0,112 | 60 | 1,213 | 0,94 | 1,39 | 0,105 | 80 | 1,048 | 0,90 | 1,21 | 0,079 | 0,4467 | | 0,1500 | ** | 0,5510 | |
