# Supplementary material for: Late Pleistocene-Holocene paleobiogeography of the genus Apodemus in Central Europe
Source: PLoS One. 2017 Mar 10;12(3):e0173668. doi: 10.1371/journal.pone.0173668 (PMC5345881; doi:10.1371/journal.pone.0173668)
Supplement: S3 Table — (PDF) [file pone.0173668.s004.pdf]

Supplementary file IIIb: Testing effects of tooth wear (ABR) and determination strategy (SPECIES, PARATAXON SPB and SPC) upon state of particular metric and non-metric variables (one-way ANOVA). Note negligible effects of tooth wear upon the variables applied in discrimination procedures) and supreme effects of determination strategy operating with parataxa.

ANOVA, *A.agrarius*, categorial variables ABR

A.agrarius	***SS Effect***	***Df Effect***	***MS Effect***	***SS Error***	***Df Error***	***MS Error***	***F***	**p**
M1U	0.035	5	0.007	0.079	17	0.005	1.5081	0.239329
M2U	0,011	5	0,002	0,046	17	0,003	0,7990	0,565451
M3U	0,008	5	0,002	0,016	17	0,001	1,6843	0,192224
M4U	0,012	5	0,002	0,029	17	0,002	1,4289	0,264122
M5U	0,008	5	0,002	0,034	17	0,002	0,7861	0,573756
**M6U**	**0,089**	**5**	**0,018**	**0,102**	**17**	**0,006**	**2,9663**	**0,041902**
**M7U**	**0,153**	**5**	**0,031**	**0,083**	**17**	**0,005**	**6,2648**	**0,001808**
M8U	0,002	5	0,000	0,019	17	0,001	0,3135	0,897997
M9U	0,006	5	0,001	0,019	17	0,001	1,0021	0,446134
M10U	0,007	5	0,001	0,013	17	0,001	1,8446	0,157644
M11U	0,006	5	0,001	0,025	17	0,001	0,8173	0,553839
M12U	0,017	5	0,003	0,045	17	0,003	1,3033	0,308717
M13U	0,020	5	0,004	0,053	17	0,003	1,3111	0,305719
**M14U**	**0,024**	**5**	**0,005**	**0,025**	**17**	**0,001**	**3,2767**	**0,029744**
**M15U**	**0,022**	**5**	**0,004**	**0,023**	**17**	**0,001**	**3,1792**	**0,033086**
M16U	0,012	5	0,002	0,022	17	0,001	1,8506	0,156485
M17U	0,011	5	0,002	0,019	17	0,001	1,9228	0,143189
M18U	0,045	6	0,007	0,050	16	0,003	2,3723	0,078413
**M19U**	**0,012**	**6**	**0,002**	**0,010**	**16**	**0,001**	**3,2035**	**0,029175**
M20U	0,006	6	0,001	0,019	16	0,001	0,8524	0,549056
M21U	0,007	6	0,001	0,032	16	0,002	0,6194	0,712280
M22U	0,047	6	0,008	0,046	16	0,003	2,7017	0,052429
M23U	0,006	6	0,001	0,016	16	0,001	1,0492	0,431197
M24U	0,007	6	0,001	0,009	16	0,001	1,9597	0,132190
M25U	0,007	6	0,001	0,045	16	0,003	0,3931	0,872711
M26U	0,011	6	0,002	0,027	16	0,002	1,0640	0,423175
**M27U**	**0,018**	**6**	**0,003**	**0,015**	**16**	**0,001**	**3,3304**	**0,025286**
M28U	0,011	6	0,002	0,018	16	0,001	1,6524	0,197011
M29U	0,005	6	0,001	0,017	16	0,001	0,7989	0,584668
M30U	0,009	6	0,001	0,020	16	0,001	1,2048	0,353453
m1L	0,037	7	0,005	0,054	15	0,004	1,4701	0,250600
m2L	0,038	7	0,005	0,041	15	0,003	2,0040	0,122401
**m3L**	**0,100**	**7**	**0,014**	**0,051**	**15**	**0,003**	**4,2338**	**0,009103**
**m4L**	**0,062**	**7**	**0,009**	**0,047**	**15**	**0,003**	**2,8067**	**0,044269**
**m5L**	**0,024**	**7**	**0,003**	**0,012**	**15**	**0,001**	**4,3120**	**0,008412**
m6L	0,022	7	0,003	0,035	15	0,002	1,3424	0,297784
**m7L**	**0,028**	**7**	**0,004**	**0,019**	**15**	**0,001**	**3,2058**	**0,027635**
**m8L**	**0,073**	**7**	**0,010**	**0,023**	**15**	**0,002**	**6,8563**	**0,000915**
**m9L**	**0,067**	**7**	**0,010**	**0,020**	**15**	**0,001**	**7,3972**	**0,000613**
m10L	0,001	7	0,000	0,011	15	0,001	0,2507	0,964029
m11L	0,000	7	0,000	0,006	15	0,000	0,1545	0,990704
**m12L**	**0,048**	**7**	**0,007**	**0,027**	**15**	**0,002**	**3,8661**	**0,013325**
**m13L**	**0,088**	**7**	**0,013**	**0,020**	**15**	**0,001**	**9,5440**	**0,000149**
m14L	0,019	7	0,003	0,020	15	0,001	2,0076	0,121813
m15L	0,019	7	0,003	0,015	15	0,001	2,6397	0,054291
**m16L**	**0,049**	**6**	**0,008**	**0,026**	**16**	**0,002**	**5,0549**	**0,004384**
**m17L**	**0,096**	**6**	**0,016**	**0,040**	**16**	**0,003**	**6,3464**	**0,001438**
**m18L**	**0,027**	**6**	**0,005**	**0,024**	**16**	**0,002**	**2,9526**	**0,038951**
**m19L**	**0,096**	**6**	**0,016**	**0,031**	**16**	**0,002**	**8,2466**	**0,000351**
**m20L**	**0,277**	**6**	**0,046**	**0,034**	**16**	**0,002**	**21,9901**	**0,000001**
**m21L**	**0,102**	**6**	**0,017**	**0,027**	**16**	**0,002**	**9,9102**	**0,000121**
**m22L**	**0,033**	**6**	**0,005**	**0,026**	**16**	**0,002**	**3,3273**	**0,025375**
**m23L**	**0,038**	**6**	**0,006**	**0,011**	**16**	**0,001**	**9,0259**	**0,000210**
**m24L**	**0,076**	**6**	**0,013**	**0,036**	**16**	**0,002**	**5,6499**	**0,002575**
**m25L**	**0,096**	**6**	**0,016**	**0,032**	**16**	**0,002**	**8,1560**	**0,000373**
m26L	0,005	6	0,001	0,016	16	0,001	0,8261	0,566373
m27L	0,002	6	0,000	0,012	16	0,001	0,3898	0,874823
M2U/M1U	0,005	5	0,001	0,008	17	0,000	1,9916	0,131626
M14U/M15U	0,004	5	0,001	0,010	17	0,001	1,5114	0,238331
**m3L/m1L**	**0,045**	**7**	**0,006**	**0,017**	**15**	**0,001**	**5,7332**	**0,002261**
m6L/m5L	0,005	7	0,001	0,051	15	0,003	0,2252	0,972987
F1	18,006	5	3,601	80,584	17	4,740	0,7597	0,590938
F2	18,257	5	3,651	42,164	17	2,480	1,4722	0,250245
F3	4,659	5	0,932	20,952	17	1,232	0,7560	0,593396

F4	11,627	5	2,325	28,286	17	1,664	1,3976	0,274588
F5	1,445	5	0,289	14,381	17	0,846	0,3417	0,880510
F6	0,000	5	0,000	0,000	17	0,000		
F7	0,000	5	0,000	0,000	17	0,000		
**F8**	**53,392**	**5**	**10,678**	**45,179**	**17**	**2,658**	**4,0181**	**0,013697**
F9	25,497	5	5,099	49,074	17	2,887	1,7665	0,173612
F10	0,000	6	0,000	0,000	16	0,000		
**F11**	**26,033**	**6**	**4,339**	**19,125**	**16**	**1,195**	**3,6299**	**0,018191**
F12	2,935	6	0,489	13,500	16	0,844	0,5797	0,741345
F13	10,875	6	1,813	34,125	16	2,133	0,8498	0,550747
F14	3,033	7	0,433	19,467	15	1,298	0,3339	0,926020
F15	14,087	7	2,012	20,018	15	1,335	1,5080	0,238077
F16	1,311	7	0,187	4,967	15	0,331	0,5657	0,772493
F17	4,694	7	0,671	4,417	15	0,294	2,2776	0,085681
F18	7,394	7	1,056	28,217	15	1,881	0,5616	0,775544
F19	0,228	7	0,033	1,550	15	0,103	0,3149	0,935806
F20	7,033	7	1,005	18,967	15	1,264	0,7946	0,603401
F21	8,092	7	1,156	8,967	15	0,598	1,9339	0,134302
F22	11,033	6	1,839	22,967	16	1,435	1,2811	0,320202
F23	1,800	6	0,300	20,700	16	1,294	0,2319	0,959925
**F24**	**30,625**	**6**	**5,104**	**21,875**	**16**	**1,367**	**3,7333**	**0,016277**
SURM1	0,155	5	0,031	0,296	17	0,017	1,7779	0,171182
SURM2	0,052	6	0,009	0,088	16	0,005	1,5883	0,214251
**SURm1**	**0,165**	**7**	**0,024**	**0,095**	**15**	**0,006**	**3,7302**	**0,015407**
**SURm2**	**0,260**	**6**	**0,043**	**0,079**	**16**	**0,005**	**8,7562**	**0,000250**

ANOVA, *A.flavicollis*, categorial variables ABR

A.flavicollis	***SS Effect***	***Df Effect***	***MS Effect***	***SS Error***	***Df Error***	***MS Error***	***F***	**p**
**M1U**	**0,071**	**6**	**0,012**	**0,351**	**68**	**0,005**	**2,2983**	**0,044388**
**M2U**	**0,049**	**6**	**0,008**	**0,135**	**68**	**0,002**	**4,0869**	**0,001471**
M3U	0,004	6	0,001	0,064	68	0,001	0,7871	0,583073
M4U	0,022	6	0,004	0,179	68	0,003	1,4192	0,220051
M5U	0,016	6	0,003	0,232	68	0,003	0,7606	0,603357
**M6U**	**0,387**	**6**	**0,064**	**0,182**	**68**	**0,003**	**24,0843**	**0,000000**
**M7U**	**0,257**	**6**	**0,043**	**0,171**	**68**	**0,003**	**16,9536**	**0,000000**
M8U	0,003	6	0,000	0,060	68	0,001	0,5570	0,762837
**M9U**	**0,020**	**6**	**0,003**	**0,088**	**68**	**0,001**	**2,6275**	**0,023748**
**M10U**	**0,035**	**6**	**0,006**	**0,115**	**68**	**0,002**	**3,4595**	**0,004835**
**M11U**	**0,039**	**6**	**0,007**	**0,078**	**68**	**0,001**	**5,6504**	**0,000083**
**M12U**	**0,060**	**6**	**0,010**	**0,117**	**68**	**0,002**	**5,8039**	**0,000063**
**M13U**	**0,058**	**6**	**0,010**	**0,148**	**68**	**0,002**	**4,4225**	**0,000784**
M14U	0,020	6	0,003	0,111	68	0,002	2,0262	0,073945
M15U	0,020	6	0,003	0,100	68	0,001	2,2270	0,050775
M16U	0,007	6	0,001	0,076	68	0,001	1,0620	0,393977
M17U	0,015	6	0,003	0,082	68	0,001	2,1445	0,059286
**M18U**	**0,048**	**6**	**0,008**	**0,242**	**68**	**0,004**	**2,2358**	**0,049943**
M19U	0,012	6	0,002	0,070	68	0,001	1,9571	0,084046
M20U	0,009	6	0,001	0,144	68	0,002	0,6870	0,660686
M21U	0,014	6	0,002	0,178	68	0,003	0,8926	0,505412
**M22U**	**0,117**	**6**	**0,020**	**0,131**	**68**	**0,002**	**10,1576**	**0,000000**
M23U	0,008	6	0,001	0,093	68	0,001	1,0254	0,416505
**M24U**	**0,028**	**6**	**0,005**	**0,067**	**68**	**0,001**	**4,8401**	**0,000362**
**M25U**	**0,029**	**6**	**0,005**	**0,141**	**68**	**0,002**	**2,3646**	**0,039161**
**M26U**	**0,050**	**6**	**0,008**	**0,138**	**68**	**0,002**	**4,1170**	**0,001390**
M27U	0,006	6	0,001	0,078	68	0,001	0,9117	0,491958
M28U	0,010	6	0,002	0,085	68	0,001	1,4063	0,224990
M29U	0,021	6	0,004	0,114	68	0,002	2,1074	0,063553
M30U	0,016	6	0,003	0,136	68	0,002	1,3652	0,241255
m1L	0,031	5	0,006	0,292	68	0,004	1,4623	0,213657
**m2L**	**0,184**	**5**	**0,037**	**0,326**	**68**	**0,005**	**7,6722**	**0,000009**
**m3L**	**0,301**	**5**	**0,060**	**0,267**	**68**	**0,004**	**15,3413**	**0,000000**
**m4L**	**0,376**	**5**	**0,075**	**0,205**	**68**	**0,003**	**24,8906**	**0,000000**
m5L	0,022	5	0,004	0,132	68	0,002	2,2812	0,056089
m6L	0,016	5	0,003	0,162	68	0,002	1,3583	0,250987
**m7L**	**0,318**	**5**	**0,064**	**0,171**	**68**	**0,003**	**25,2242**	**0,000000**
**m8L**	**0,144**	**5**	**0,029**	**0,089**	**68**	**0,001**	**21,8824**	**0,000000**
**m9L**	**0,096**	**5**	**0,019**	**0,139**	**68**	**0,002**	**9,3197**	**0,000001**
**m10L**	**0,011**	**5**	**0,002**	**0,052**	**68**	**0,001**	**2,8720**	**0,020651**
m11L	0,004	5	0,001	0,077	68	0,001	0,7899	0,560626
m12L	0,041	5	0,008	0,329	68	0,005	1,6964	0,147307

**m13L**	**0,073**	**5**	**0,015**	**0,197**	**68**	**0,003**	**5,0329**	**0,000555**
**m14L**	**0,049**	**5**	**0,010**	**0,251**	**68**	**0,004**	**2,6569**	**0,029748**
m15L	0,025	5	0,005	0,204	68	0,003	1,6820	0,150758
**m16L**	**0,038**	**5**	**0,008**	**0,171**	**68**	**0,003**	**3,0493**	**0,015281**
**m17L**	**0,147**	**5**	**0,029**	**0,077**	**68**	**0,001**	**25,9638**	**0,000000**
**m18L**	**0,210**	**5**	**0,042**	**0,187**	**68**	**0,003**	**15,3058**	**0,000000**
**m19L**	**0,022**	**5**	**0,004**	**0,121**	**68**	**0,002**	**2,5050**	**0,038467**
m20L	0,023	5	0,005	0,147	68	0,002	2,1440	0,070569
**m21L**	**0,207**	**5**	**0,041**	**0,185**	**68**	**0,003**	**15,1725**	**0,000000**
**m22L**	**0,021**	**5**	**0,004**	**0,104**	**68**	**0,002**	**2,7835**	**0,024001**
**m23L**	**0,033**	**5**	**0,007**	**0,135**	**68**	**0,002**	**3,3178**	**0,009689**
**m24L**	**0,237**	**5**	**0,047**	**0,313**	**68**	**0,005**	**10,2799**	**0,000000**
**m25L**	**0,150**	**5**	**0,030**	**0,152**	**68**	**0,002**	**13,4465**	**0,000000**
**m26L**	**0,032**	**5**	**0,006**	**0,178**	**68**	**0,003**	**2,4565**	**0,041753**
**m27L**	**0,034**	**5**	**0,007**	**0,197**	**68**	**0,003**	**2,3618**	**0,048980**
**M2U/M1U**	**0,008**	**6**	**0,001**	**0,018**	**68**	**0,000**	**5,0689**	**0,000238**
**M14U/M15U**	**0,025**	**6**	**0,004**	**0,109**	**68**	**0,002**	**2,6440**	**0,023011**
**m3L/m1L**	**0,103**	**5**	**0,021**	**0,057**	**68**	**0,001**	**24,6722**	**0,000000**
m6L/m5L	0,001	5	0,000	0,054	68	0,001	0,2055	0,959113
**F1**	**137,970**	**6**	**22,995**	**354,750**	**68**	**5,217**	**4,4078**	**0,000806**
**F2**	**66,574**	**6**	**11,096**	**89,264**	**68**	**1,313**	**8,4525**	**0,000001**
F3	20,448	6	3,408	108,417	68	1,594	2,1376	0,060066
**F4**	**175,000**	**6**	**29,167**	**507,000**	**68**	**7,456**	**3,9119**	**0,002046**
**F5**	**66,250**	**6**	**11,042**	**316,764**	**68**	**4,658**	**2,3703**	**0,038736**
F6	1,500	6	0,250	40,500	68	0,596	0,4198	0,863418
F7	73,045	6	12,174	456,875	68	6,719	1,8120	0,109698
**F8**	**77,534**	**6**	**12,922**	**384,750**	**68**	**5,658**	**2,2839**	**0,045617**
F9	6,625	6	1,104	173,375	68	2,550	0,4331	0,854334
**F10**	**36,810**	**6**	**6,135**	**121,204**	**68**	**1,782**	**3,4420**	**0,005000**
**F11**	**69,748**	**6**	**11,625**	**73,512**	**68**	**1,081**	**10,7531**	**0,000000**
F12	55,901	6	9,317	501,606	68	7,377	1,2630	0,286080
F13	2,028	6	0,338	255,847	68	3,762	0,0898	0,997139
F14	23,561	5	4,712	238,385	68	3,506	1,3442	0,256466
F15	19,131	5	3,826	160,991	68	2,368	1,6161	0,167562
**F16**	**18,726**	**5**	**3,745**	**83,233**	**68**	**1,224**	**3,0598**	**0,015012**
F17	26,747	5	5,349	243,691	68	3,584	1,4927	0,203720
F18	13,839	5	2,768	288,134	68	4,237	0,6532	0,660051
F19	11,715	5	2,343	370,175	68	5,444	0,4304	0,825890
F20	14,717	5	2,943	112,918	68	1,661	1,7725	0,130223
F21	9,284	5	1,857	134,878	68	1,984	0,9361	0,463347
F22	32,305	5	6,461	394,344	68	5,799	1,1141	0,361192
**F23**	**67,872**	**5**	**13,574**	**275,608**	**68**	**4,053**	**3,3492**	**0,009187**
**F24**	**56,818**	**5**	**11,364**	**148,046**	**68**	**2,177**	**5,2195**	**0,000410**
SURM1	0,359	6	0,060	2,391	68	0,035	1,7027	0,133695
SURM2	0,103	6	0,017	1,104	68	0,016	1,0573	0,396826
SURm1	0,199	5	0,040	1,501	68	0,022	1,8059	0,123333
**SURm2**	**0,167**	**5**	**0,033**	**0,749**	**68**	**0,011**	**3,0410**	**0,015498**

ANOVA, *A.sylvaticus*, categorial variables ABR

A.sylvaticus	***SS Effect***	***Df Effect***	***MS Effect***	***SS Error***	***Df Error***	***MS Error***	***F***	**p**
M1U	0,016	6	0,003	0,235	56	0,004	0,6379	0,699365
**M2U**	**0,068**	**6**	**0,011**	**0,101**	**56**	**0,002**	**6,2126**	**0,000048**
M3U	0,002	6	0,000	0,052	56	0,001	0,3088	0,929800
M4U	0,007	6	0,001	0,141	56	0,003	0,4917	0,811862
M5U	0,007	6	0,001	0,168	56	0,003	0,3955	0,878833
**M6U**	**0,480**	**6**	**0,080**	**0,147**	**56**	**0,003**	**30,4598**	**0,000000**
**M7U**	**0,266**	**6**	**0,044**	**0,296**	**56**	**0,005**	**8,3701**	**0,000002**
M8U	0,010	6	0,002	0,049	56	0,001	1,8522	0,105468
**M9U**	**0,024**	**6**	**0,004**	**0,062**	**56**	**0,001**	**3,5742**	**0,004552**
**M10U**	**0,069**	**6**	**0,012**	**0,088**	**56**	**0,002**	**7,3350**	**0,000008**
**M11U**	**0,033**	**6**	**0,005**	**0,087**	**56**	**0,002**	**3,5199**	**0,005024**
**M12U**	**0,073**	**6**	**0,012**	**0,098**	**56**	**0,002**	**6,9323**	**0,000015**
**M13U**	**0,040**	**6**	**0,007**	**0,111**	**56**	**0,002**	**3,4164**	**0,006065**
M14U	0,012	6	0,002	0,059	56	0,001	1,9615	0,086752
M15U	0,001	6	0,000	0,082	56	0,001	0,1650	0,984996

M16U	0,006	6	0,001	0,059	56	0,001	0,9970	0,436465
M17U	0,028	6	0,005	0,125	56	0,002	2,0737	0,070858
**M18U**	**0,095**	**7**	**0,014**	**0,171**	**55**	**0,003**	**4,3745**	**0,000646**
M19U	0,010	7	0,001	0,042	55	0,001	1,9616	0,077202
M20U	0,012	7	0,002	0,095	55	0,002	0,9867	0,450493
M21U	0,013	7	0,002	0,119	55	0,002	0,8688	0,536853
**M22U**	**0,122**	**7**	**0,017**	**0,129**	**55**	**0,002**	**7,4395**	**0,000003**
M23U	0,019	7	0,003	0,068	55	0,001	2,1660	0,051548
M24U	0,004	7	0,001	0,052	55	0,001	0,6283	0,730356
**M25U**	**0,044**	**7**	**0,006**	**0,103**	**55**	**0,002**	**3,3465**	**0,004835**
**M26U**	**0,057**	**7**	**0,008**	**0,069**	**55**	**0,001**	**6,5099**	**0,000013**
**M27U**	**0,018**	**7**	**0,003**	**0,047**	**55**	**0,001**	**2,9198**	**0,011361**
**M28U**	**0,015**	**7**	**0,002**	**0,047**	**55**	**0,001**	**2,4983**	**0,026519**
M29U	0,015	7	0,002	0,059	55	0,001	2,0081	0,070456
M30U	0,012	7	0,002	0,060	55	0,001	1,5770	0,161745
m1L	0,045	6	0,008	0,265	56	0,005	1,5868	0,168085
m2L	0,051	6	0,009	0,392	56	0,007	1,2220	0,308902
**m3L**	**0,307**	**6**	**0,051**	**0,213**	**56**	**0,004**	**13,4871**	**0,000000**
**m4L**	**0,226**	**6**	**0,038**	**0,284**	**56**	**0,005**	**7,4355**	**0,000007**
m5L	0,021	6	0,003	0,170	56	0,003	1,1498	0,346226
**m6L**	**0,046**	**6**	**0,008**	**0,109**	**56**	**0,002**	**3,9456**	**0,002329**
**m7L**	**0,140**	**6**	**0,023**	**0,116**	**56**	**0,002**	**11,2680**	**0,000000**
**m8L**	**0,255**	**6**	**0,042**	**0,129**	**56**	**0,002**	**18,3977**	**0,000000**
**m9L**	**0,110**	**6**	**0,018**	**0,110**	**56**	**0,002**	**9,2995**	**0,000000**
m10L	0,012	6	0,002	0,098	56	0,002	1,1409	0,351071
m11L	0,002	6	0,000	0,063	56	0,001	0,3268	0,920142
**m12L**	**0,104**	**6**	**0,017**	**0,375**	**56**	**0,007**	**2,5795**	**0,028135**
**m13L**	**0,128**	**6**	**0,021**	**0,146**	**56**	**0,003**	**8,1663**	**0,000002**
m14L	0,024	6	0,004	0,285	56	0,005	0,7849	0,585438
m15L	0,026	6	0,004	0,225	56	0,004	1,0688	0,392155
m16L	0,019	7	0,003	0,194	55	0,004	0,7539	0,627858
**m17L**	**0,360**	**7**	**0,051**	**0,146**	**55**	**0,003**	**19,4085**	**0,000000**
**m18L**	**0,141**	**7**	**0,020**	**0,134**	**55**	**0,002**	**8,2410**	**0,000001**
m19L	0,021	7	0,003	0,136	55	0,002	1,2125	0,311644
m20L	0,033	7	0,005	0,175	55	0,003	1,4831	0,192569
**m21L**	**0,236**	**7**	**0,034**	**0,092**	**55**	**0,002**	**20,1200**	**0,000000**
**m22L**	**0,077**	**7**	**0,011**	**0,099**	**55**	**0,002**	**6,1021**	**0,000027**
m23L	0,013	7	0,002	0,085	55	0,002	1,1618	0,339630
**m24L**	**0,174**	**7**	**0,025**	**0,184**	**55**	**0,003**	**7,4144**	**0,000003**
**m25L**	**0,259**	**7**	**0,037**	**0,094**	**55**	**0,002**	**21,6301**	**0,000000**
m26L	0,017	7	0,002	0,108	55	0,002	1,2655	0,284435
m27L	0,044	7	0,006	0,197	55	0,004	1,7504	0,116364
**M2U/M1U**	**0,015**	**6**	**0,002**	**0,017**	**56**	**0,000**	**8,1889**	**0,000002**
**M14U/M15U**	**0,019**	**6**	**0,003**	**0,069**	**56**	**0,001**	**2,5504**	**0,029678**
**m3L/m1L**	**0,144**	**6**	**0,024**	**0,082**	**56**	**0,001**	**16,4509**	**0,000000**
**m6L/m5L**	**0,020**	**6**	**0,003**	**0,040**	**56**	**0,001**	**4,6954**	**0,000619**
**F1**	**123,378**	**6**	**20,563**	**303,479**	**56**	**5,419**	**3,7944**	**0,003057**
**F2**	**140,176**	**6**	**23,363**	**110,046**	**56**	**1,965**	**11,8887**	**0,000000**
**F3**	**68,473**	**6**	**11,412**	**177,009**	**56**	**3,161**	**3,6104**	**0,004263**
F4	76,652	6	12,775	522,598	56	9,332	1,3690	0,243130
F5	13,375	6	2,229	144,834	56	2,586	0,8619	0,528534
F6	13,461	6	2,244	73,968	56	1,321	1,6985	0,138381
F7	86,065	6	14,344	435,363	56	7,774	1,8451	0,106821
**F8**	**230,017**	**6**	**38,336**	**245,697**	**56**	**4,387**	**8,7377**	**0,000001**
**F9**	**169,355**	**6**	**28,226**	**167,502**	**56**	**2,991**	**9,4366**	**0,000000**
F10	29,869	7	4,267	143,835	55	2,615	1,6316	0,145939
**F11**	**139,413**	**7**	**19,916**	**132,679**	**55**	**2,412**	**8,2559**	**0,000001**
F12	30,599	7	4,371	223,329	55	4,061	1,0765	0,390834
**F13**	**2100,717**	**7**	**300,102**	**6716,250**	**55**	**122,114**	**2,4576**	**0,028780**
F14	14,485	6	2,414	162,765	56	2,907	0,8306	0,551333
F15	31,047	6	5,174	194,824	56	3,479	1,4874	0,199291
F16	15,022	6	2,504	80,495	56	1,437	1,7418	0,128245
F17	1,960	6	0,327	153,696	56	2,745	0,1190	0,993716
F18	34,126	6	5,688	244,009	56	4,357	1,3053	0,269994

F19	5,373	6	0,895	156,627	56	2,797	0,3202	0,923770
F20	4,261	6	0,710	127,343	56	2,274	0,3123	0,927975
F21	7,921	6	1,320	181,974	56	3,250	0,4063	0,871823
F22	15,112	7	2,159	155,738	55	2,832	0,7624	0,620956
F23	10,604	7	1,515	415,199	55	7,549	0,2007	0,984018
F24	1137,632	7	162,519	7964,506	55	144,809	1,1223	0,362683
SURM1	0,051	6	0,008	1,197	56	0,021	0,3959	0,878582
SURM2	0,119	7	0,017	0,525	55	0,010	1,7830	0,109292
SURm1	0,120	6	0,020	1,171	56	0,021	0,9553	0,463739
SURm2	0,057	7	0,008	0,597	55	0,011	0,7461	0,634142

ANOVA, *A.uralensis*, categorial variables ABR

A.uralensis	***SS Effect***	***Df Effect***	***MS Effect***	***SS Error***	***Df Error***	***MS Error***	***F***	**p**
**M1U**	**0,115**	**6**	**0,019**	**0,584**	**80**	**0,007**	**2,625**	**0,022479**
**M2U**	**0,089**	**6**	**0,015**	**0,217**	**80**	**0,003**	**5,431**	**0,000094**
M3U	0,010	6	0,002	0,108	80	0,001	1,273	0,279226
M4U	0,009	6	0,002	0,163	80	0,002	0,761	0,603047
M5U	0,013	6	0,002	0,166	80	0,002	1,016	0,420990
**M6U**	**0,223**	**6**	**0,037**	**0,179**	**80**	**0,002**	**16,585**	**0,000000**
**M7U**	**0,166**	**6**	**0,028**	**0,250**	**80**	**0,003**	**8,852**	**0,000000**
M8U	0,004	6	0,001	0,074	80	0,001	0,757	0,605590
M9U	0,004	6	0,001	0,097	80	0,001	0,591	0,736652
**M10U**	**0,018**	**6**	**0,003**	**0,103**	**80**	**0,001**	**2,391**	**0,035507**
M11U	0,013	6	0,002	0,143	80	0,002	1,202	0,313908
**M12U**	**0,072**	**6**	**0,012**	**0,225**	**80**	**0,003**	**4,270**	**0,000880**
**M13U**	**0,076**	**6**	**0,013**	**0,203**	**80**	**0,003**	**4,990**	**0,000218**
M14U	0,008	6	0,001	0,096	80	0,001	1,150	0,341449
M15U	0,014	6	0,002	0,092	80	0,001	2,003	0,074919
M16U	0,007	6	0,001	0,120	80	0,001	0,760	0,603392
M17U	0,007	6	0,001	0,116	80	0,001	0,769	0,596526
**M18U**	**0,054**	**5**	**0,011**	**0,1843**	**81**	**0,002**	**4,750**	**0,000741**
M19U	0,007	5	0,001	0,0592	81	0,001	1,847	0,112970
M20U	0,008	5	0,002	0,1366	81	0,002	0,970	0,441324
M21U	0,013	5	0,003	0,2386	81	0,003	0,877	0,500230
**M22U**	**0,135**	**5**	**0,027**	**0,2475**	**81**	**0,003**	**8,866**	**0,000001**
**M23U**	**0,017**	**5**	**0,003**	**0,0806**	**81**	**0,001**	**3,375**	**0,008059**
**M24U**	**0,029**	**5**	**0,006**	**0,0840**	**81**	**0,001**	**5,645**	**0,000163**
M25U	0,007	5	0,001	0,1214	81	0,001	0,882	0,497292
M26U	0,010	5	0,002	0,1159	81	0,001	1,416	0,227445
M27U	0,003	5	0,001	0,0959	81	0,001	0,516	0,763734
M28U	0,006	5	0,001	0,0910	81	0,001	1,128	0,352258
**M29U**	**0,021**	**5**	**0,004**	**0,1009**	**81**	**0,001**	**3,354**	**0,008369**
**M30U**	**0,015**	**5**	**0,003**	**0,0929**	**81**	**0,001**	**2,647**	**0,028746**
m1L	0,017	5	0,003	0,2838	77	0,004	0,948	0,455164
m2L	0,037	5	0,007	0,2602	77	0,003	2,172	0,065815
**m3L**	**0,115**	**5**	**0,023**	**0,1876**	**77**	**0,002**	**9,477**	**0,000000**
m4L	0,094	5	0,019	0,3025	77	0,004	4,809	0,000705
m5L	0,008	5	0,002	0,1572	77	0,002	0,783	0,564918
M6L	0,021	5	0,004	0,2025	77	0,003	1,616	0,165739
**m7L**	**0,249**	**5**	**0,050**	**0,1654**	**77**	**0,002**	**23,225**	**0,000000**
**m8L**	**0,236**	**5**	**0,047**	**0,1355**	**77**	**0,002**	**26,862**	**0,000000**
**m9L**	**0,080**	**5**	**0,016**	**0,1671**	**77**	**0,002**	**7,413**	**0,000010**
**m10L**	**0,014**	**5**	**0,003**	**0,0511**	**77**	**0,001**	**4,225**	**0,001903**
m11L	0,005	5	0,001	0,0613	77	0,001	1,147	0,343106
**m12L**	**0,061**	**5**	**0,012**	**0,2247**	**77**	**0,003**	**4,170**	**0,002095**
**m13L**	**0,077**	**5**	**0,015**	**0,1570**	**77**	**0,002**	**7,593**	**0,000008**
**m14L**	**0,022**	**5**	**0,004**	**0,1374**	**77**	**0,002**	**2,414**	**0,043571**
**m15L**	**0,020**	**5**	**0,004**	**0,1064**	**77**	**0,001**	**2,895**	**0,018981**
**m16L**	**0,035**	**6**	**0,006**	**0,1333**	**73**	**0,002**	**3,209**	**0,007481**
**m17L**	**0,312**	**6**	**0,052**	**0,0847**	**73**	**0,001**	**44,830**	**0,000000**
**m18L**	**0,187**	**6**	**0,031**	**0,0808**	**73**	**0,001**	**28,231**	**0,000000**
m19L	0,015	6	0,003	0,1306	73	0,002	1,437	0,212307
m20L	0,021	6	0,003	0,1598	73	0,002	1,567	0,169160
**m21L**	**0,301**	**6**	**0,050**	**0,1648**	**73**	**0,002**	**22,193**	**0,000000**
**m22L**	**0,086**	**6**	**0,014**	**0,1687**	**73**	**0,002**	**6,181**	**0,000028**
**m23L**	**0,032**	**6**	**0,005**	**0,1550**	**73**	**0,002**	**2,480**	**0,030718**
**m24L**	**0,236**	**6**	**0,039**	**0,0837**	**73**	**0,001**	**34,342**	**0,000000**

**m25L**	**0,224**	**6**	**0,037**	**0,1212**	**73**	**0,002**	**22,534**	**0,000000**
**m26L**	**0,024**	**6**	**0,004**	**0,1127**	**73**	**0,002**	**2,551**	**0,026779**
**m27L**	**0,059**	**6**	**0,010**	**0,1015**	**73**	**0,001**	**7,040**	**0,000006**
**M2U/M1U**	**0,014**	**6**	**0,002**	**0,029**	**80**	**0,000**	**6,599**	**0,000011**
M14U/M15U	0,007	6	0,001	0,104	80	0,001	0,886	0,509493
**m3L/m1L**	**0,062**	**5**	**0,012**	**0,0481**	**77**	**0,001**	**19,809**	**0,000000**
m6L/m5L	0,010	5	0,002	0,0821	77	0,001	1,887	0,106304
**F1**	**103,626**	**6**	**17,271**	**534,029**	**80**	**6,675**	**2,587**	**0,024204**
**F2**	**41,598**	**6**	**6,933**	**156,080**	**80**	**1,951**	**3,554**	**0,003595**
F3	16,984	6	2,831	191,637	80	2,395	1,182	0,324509
F4	63,574	6	10,596	1018,495	80	12,731	0,832	0,548511
F5	24,444	6	4,074	294,614	80	3,683	1,106	0,366147
F6	9,046	6	1,508	124,609	80	1,558	0,968	0,452404
**F7**	**110,569**	**6**	**18,428**	**559,362**	**80**	**6,992**	**2,636**	**0,022017**
F8	14,698	6	2,450	195,716	80	2,446	1,001	0,430432
**F9**	**29,068**	**6**	**4,845**	**94,036**	**80**	**1,175**	**4,121**	**0,001176**
F10	25,855	5	5,171	314,9958	81	3,889	1,330	0,260013
F11	15,160	5	3,032	136,0959	81	1,680	1,805	0,121181
F12	14,026	5	2,805	713,6289	81	8,810	0,318	0,900579
F13	16,702	5	3,340	244,1944	81	3,015	1,108	0,362695
F14	39,957	5	7,991	360,9104	77	4,687	1,705	0,143493
F15	19,821	5	3,964	238,2517	77	3,094	1,281	0,280699
F16	19,124	5	3,825	127,1169	77	1,651	2,317	0,051427
F17	31,391	5	6,278	362,8503	77	4,712	1,332	0,259575
F18	22,706	5	4,541	431,2455	77	5,601	0,811	0,545504
F19	58,812	5	11,762	517,8631	77	6,725	1,749	0,133529
F20	8,432	5	1,686	149,2309	77	1,938	0,870	0,505204
**F21**	**40,599**	**5**	**8,120**	**179,5695**	**77**	**2,332**	**3,482**	**0,006863**
F22	36,433	6	6,072	424,3671	73	5,813	1,045	0,403897
F23	52,211	6	8,702	480,5889	73	6,583	1,322	0,258447
F24	23,200	6	3,867	148,2875	73	2,031	1,904	0,091655
SURM1	0,239	6	0,040	1,956	80	0,024	1,627	0,150541
**SURM2**	**0,095**	**5**	**0,019**	**0,6330**	**81**	**0,008**	**2,443**	**0,040988**
**SURm2**	**0,096**	**6**	**0,016**	**0,4002**	**73**	**0,005**	**2,906**	**0,013470**
SURm1	0,061	5	0,012	1,0966	77	0,014	0,863	0,510024

ANOVA, Recent samples, categorial variables SPECIES

	SS Effect	***Df Effect***	***MS Effect***	***SS Error***	***Df Error***	***MS Error***	***F***	**p**
**M1U**	**6,747**	**3**	**2,249**	**1,902**	**244**	**0,008**	**288,588**	**0,000000**
**M2U**	**0,864**	**3**	**0,288**	**0,839**	**244**	**0,003**	**83,755**	**0,000000**
**M3U**	**0,563**	**3**	**0,188**	**0,373**	**244**	**0,002**	**122,697**	**0,000000**
**M4U**	**2,223**	**3**	**0,741**	**0,724**	**244**	**0,003**	**249,847**	**0,000000**
**M5U**	**2,673**	**3**	**0,891**	**0,958**	**244**	**0,004**	**226,859**	**0,000000**
**M6U**	**1,328**	**3**	**0,443**	**2,273**	**244**	**0,009**	**47,521**	**0,000000**
**M7U**	**0,946**	**3**	**0,315**	**1,754**	**244**	**0,007**	**43,872**	**0,000000**
**M8U**	**0,239**	**3**	**0,080**	**0,316**	**244**	**0,001**	**61,603**	**0,000000**
**M9U**	**0,172**	**3**	**0,057**	**0,367**	**244**	**0,002**	**38,107**	**0,000000**
**M10U**	**0,204**	**3**	**0,068**	**0,452**	**244**	**0,002**	**36,749**	**0,000000**
**M11U**	**0,283**	**3**	**0,094**	**0,456**	**244**	**0,002**	**50,439**	**0,000000**
**M12U**	**0,793**	**3**	**0,264**	**0,820**	**244**	**0,003**	**78,683**	**0,000000**
**M13U**	**0,978**	**3**	**0,326**	**0,808**	**244**	**0,003**	**98,542**	**0,000000**
**M14U**	**1,095**	**3**	**0,365**	**0,534**	**244**	**0,002**	**166,906**	**0,000000**
**M15U**	**1,201**	**3**	**0,400**	**0,520**	**244**	**0,002**	**187,841**	**0,000000**
**M16U**	**0,612**	**3**	**0,204**	**0,411**	**244**	**0,002**	**121,150**	**0,000000**
**M17U**	**0,782**	**3**	**0,261**	**0,445**	**244**	**0,002**	**142,865**	**0,000000**
**M18U**	**3,047**	**3**	**1,016**	**0,888**	**244**	**0,004**	**279,190**	**0,000000**
**M19U**	**0,424**	**3**	**0,141**	**0,223**	**244**	**0,001**	**154,946**	**0,000000**
**M20U**	**2,029**	**3**	**0,676**	**0,429**	**244**	**0,002**	**384,710**	**0,000000**
**M21U**	**2,311**	**3**	**0,770**	**0,616**	**244**	**0,003**	**305,334**	**0,000000**
**M22U**	**1,210**	**3**	**0,403**	**0,975**	**244**	**0,004**	**100,944**	**0,000000**
**M23U**	**0,319**	**3**	**0,106**	**0,308**	**244**	**0,001**	**84,173**	**0,000000**
**M24U**	**0,214**	**3**	**0,071**	**0,281**	**244**	**0,001**	**61,849**	**0,000000**
**M25U**	**0,520**	**3**	**0,173**	**0,496**	**244**	**0,002**	**85,249**	**0,000000**
**M26U**	**0,578**	**3**	**0,193**	**0,477**	**244**	**0,002**	**98,465**	**0,000000**
**M27U**	**0,296**	**3**	**0,099**	**0,281**	**244**	**0,001**	**85,885**	**0,000000**
**M28U**	**0,390**	**3**	**0,130**	**0,284**	**244**	**0,001**	**111,785**	**0,000000**
**M29U**	**1,562**	**3**	**0,521**	**0,354**	**244**	**0,001**	**359,275**	**0,000000**

**M30U**	**1,449**	**3**	**0,483**	**0,362**	**244**	**0,001**	**325,572**	**0,000000**
**m1L**	**5,144**	**3**	**1,715**	**1,378**	**239**	**0,006**	**297,496**	**0,000000**
m2L	0,030	3	0,010	1,399	239	0,006	1,713	0,164867
**m3L**	**0,328**	**3**	**0,109**	**1,744**	**239**	**0,007**	**14,988**	**0,000000**
**m4L**	**1,811**	**3**	**0,604**	**2,136**	**239**	**0,009**	**67,557**	**0,000000**
**m5L**	**1,842**	**3**	**0,614**	**0,521**	**239**	**0,002**	**281,513**	**0,000000**
**M6L**	**1,540**	**3**	**0,513**	**0,866**	**239**	**0,004**	**141,620**	**0,000000**
**m7L**	**0,432**	**3**	**0,144**	**1,447**	**239**	**0,006**	**23,787**	**0,000000**
**m8L**	**0,248**	**3**	**0,083**	**1,359**	**239**	**0,006**	**14,515**	**0,000000**
**m9L**	**0,358**	**3**	**0,119**	**0,944**	**239**	**0,004**	**30,247**	**0,000000**
**m10L**	**0,188**	**3**	**0,063**	**0,807**	**239**	**0,003**	**18,587**	**0,000000**
**m11L**	**0,111**	**3**	**0,037**	**0,219**	**239**	**0,001**	**40,252**	**0,000000**
**m12L**	**1,285**	**3**	**0,428**	**1,549**	**239**	**0,006**	**66,106**	**0,000000**
**m13L**	**0,976**	**3**	**0,325**	**1,339**	**239**	**0,006**	**58,090**	**0,000000**
**m14L**	**1,680**	**3**	**0,560**	**1,049**	**239**	**0,004**	**127,594**	**0,000000**
**m15L**	**1,645**	**3**	**0,548**	**0,951**	**239**	**0,004**	**137,778**	**0,000000**
**m16L**	**3,135**	**3**	**1,045**	**0,337**	**236**	**0,001**	**731,679**	**0,000000**
m17L	0,009	3	0,003	1,417	236	0,006	0,492	0,688296
**m18L**	**0,284**	**3**	**0,095**	**1,139**	**236**	**0,005**	**19,626**	**0,000000**
**m19L**	**1,974**	**3**	**0,658**	**0,635**	**236**	**0,003**	**244,570**	**0,000000**
**m20L**	**2,187**	**3**	**0,729**	**0,811**	**236**	**0,003**	**212,077**	**0,000000**
**m21L**	**0,233**	**3**	**0,078**	**1,477**	**236**	**0,006**	**12,389**	**0,000000**
**m22L**	**0,612**	**3**	**0,204**	**0,794**	**236**	**0,003**	**60,643**	**0,000000**
**m23L**	**0,479**	**3**	**0,160**	**0,543**	**236**	**0,002**	**69,479**	**0,000000**
**m24L**	**0,129**	**3**	**0,043**	**1,456**	**236**	**0,006**	**6,955**	**0,000167**
**m25L**	**0,152**	**3**	**0,051**	**1,366**	**236**	**0,006**	**8,772**	**0,000015**
**m26L**	**0,952**	**3**	**0,317**	**0,543**	**236**	**0,002**	**137,840**	**0,000000**
**m27L**	**1,195**	**3**	**0,398**	**0,706**	**236**	**0,003**	**133,226**	**0,000000**
**M2U/M1U**	**0,007**	**3**	**0,002**	**0,139**	**244**	**0,001**	**3,851**	**0,010161**
**M14U/M15U**	**0,011**	**3**	**0,004**	**0,342**	**244**	**0,001**	**2,671**	**0,048101**
**m3L/m1L**	**0,070**	**3**	**0,023**	**0,579**	**239**	**0,002**	**9,586**	**0,000005**
**m6L/m5L**	**0,031**	**3**	**0,010**	**0,374**	**239**	**0,002**	**6,519**	**0,000296**
**F1**	**95,080**	**3**	**31,693**	**1772,145**	**244**	**7,263**	**4,364**	**0,005142**
F2	10,060	3	3,353	677,042	244	2,775	1,208	0,307259
**F3**	**59,131**	**3**	**19,710**	**617,609**	**244**	**2,531**	**7,787**	**0,000055**
**F4**	**401,330**	**3**	**133,777**	**2591,246**	**244**	**10,620**	**12,597**	**0,000000**
**F5**	**211,213**	**3**	**70,404**	**962,005**	**244**	**3,943**	**17,857**	**0,000000**
F6	0,567	3	0,189	266,529	244	1,092	0,173	0,914472
**F7**	**69,462**	**3**	**23,154**	**1909,232**	**244**	**7,825**	**2,959**	**0,032994**
**F8**	**297,084**	**3**	**99,028**	**1286,273**	**244**	**5,272**	**18,785**	**0,000000**
**F9**	**224,600**	**3**	**74,867**	**838,132**	**244**	**3,435**	**21,795**	**0,000000**
**F10**	**395,335**	**3**	**131,778**	**672,568**	**244**	**2,756**	**47,808**	**0,000000**
F11	3,835	3	1,278	611,767	244	2,507	0,510	0,675887
**F12**	**211,024**	**3**	**70,341**	**1555,525**	**244**	**6,375**	**11,034**	**0,000001**
**F13**	**549,513**	**3**	**183,171**	**9380,739**	**244**	**38,446**	**4,7644**	**0,003018**
**F14**	**57,544**	**3**	**19,181**	**921,244**	**239**	**3,855**	**4,976**	**0,002286**
F15	14,925	3	4,975	719,999	239	3,013	1,651	0,178213
**F16**	**22,790**	**3**	**7,597**	**638,845**	**239**	**2,673**	**2,842**	**0,038518**
**F17**	**44,652**	**3**	**14,884**	**953,839**	**239**	**3,991**	**3,729**	**0,011961**
**F18**	**124,831**	**3**	**41,610**	**1048,949**	**239**	**4,389**	**9,481**	**0,000006**
F19	15,142	3	5,047	1666,947	239	6,975	0,724	0,538725
F20	7,929	3	2,643	465,154	239	1,946	1,358	0,256300
F21	11,604	3	3,868	576,823	239	2,413	1,603	0,189428
**F22**	**171,829**	**3**	**57,276**	**1234,341**	**236**	**5,230**	**10,951**	**0,000001**
**F23**	**175,997**	**3**	**58,666**	**1451,770**	**236**	**6,152**	**9,537**	**0,000006**
F24	113,037	3	37,679	9620,560	236	40,765	0,924	0,429702
**SURM1**	**35,168**	**3**	**11,723**	**7,830**	**244**	**0,032**	**365,328**	**0,000000**
**SURM2**	**14,692**	**3**	**4,897**	**2,720**	**244**	**0,011**	**439,329**	**0,000000**
**SURm1**	**23,119**	**3**	**7,706**	**4,321**	**239**	**0,018**	**426,301**	**0,000000**
**SURm2**	**12,819**	**3**	**4,273**	**1,977**	**236**	**0,008**	**509,960**	**0,000000**

ANOVA, Recent samples, categorial variables PARATAXON SPB

	SS Effect	*Df Effect*	*MS Effect*	*SS Error*	*Df Error*	*MS Error*	*F*	p
**M1U**	**7,478**	**5**	**1,496**	**1,171**	**242**	**0,005**	**309,192**	**0,000000**
**M2U**	**1,019**	**5**	**0,204**	**0,683**	**242**	**0,003**	**72,163**	**0,000000**
**M3U**	**0,618**	**5**	**0,124**	**0,317**	**242**	**0,001**	**94,365**	**0,000000**
**M4U**	**2,561**	**5**	**0,512**	**0,386**	**242**	**0,002**	**320,936**	**0,000000**

M5U	3,020	5	0,604	0,611	242	0,003	239,182	0,000000
M6U	1,570	5	0,314	2,031	242	0,008	37,413	0,000000
M7U	1,112	5	0,222	1,587	242	0,007	33,921	0,000000
M8U	0,263	5	0,053	0,292	242	0,001	43,655	0,000000
M9U	0,208	5	0,042	0,331	242	0,001	30,426	0,000000
M10U	0,227	5	0,045	0,429	242	0,002	25,648	0,000000
M11U	0,319	5	0,064	0,420	242	0,002	36,724	0,000000
M12U	0,942	5	0,188	0,671	242	0,003	67,946	0,000000
M13U	1,155	5	0,231	0,631	242	0,003	88,511	0,000000
M14U	1,271	5	0,254	0,358	242	0,001	171,980	0,000000
M15U	1,352	5	0,270	0,369	242	0,002	177,477	0,000000
M16U	0,683	5	0,137	0,340	242	0,001	97,155	0,000000
M17U	0,837	5	0,167	0,390	242	0,002	103,857	0,000000
M18U	3,534	5	0,707	0,400	242	0,002	427,129	0,000000
M19U	0,445	5	0,089	0,202	242	0,001	106,660	0,000000
M20U	1,979	5	0,396	0,480	242	0,002	199,587	0,000000
M21U	2,277	5	0,455	0,649	242	0,003	169,779	0,000000
M22U	0,983	5	0,197	1,203	242	0,005	39,540	0,000000
M23U	0,290	5	0,058	0,337	242	0,001	41,664	0,000000
M24U	0,187	5	0,037	0,308	242	0,001	29,423	0,000000
M25U	0,582	5	0,116	0,435	242	0,002	64,843	0,000000
M26U	0,622	5	0,124	0,433	242	0,002	69,528	0,000000
M27U	0,320	5	0,064	0,257	242	0,001	60,338	0,000000
M28U	0,423	5	0,085	0,251	242	0,001	81,642	0,000000
M29U	1,611	5	0,322	0,304	242	0,001	256,210	0,000000
M30U	1,525	5	0,305	0,286	242	0,001	258,304	0,000000
m1L	5,728	5	1,146	0,794	237	0,003	341,914	0,000000
m2L	0,102	5	0,020	1,327	237	0,006	3,631	0,003489
m3L	0,438	5	0,088	1,633	237	0,007	12,717	0,000000
m4L	2,136	5	0,427	1,810	237	0,008	55,929	0,000000
m5L	2,029	5	0,406	0,334	237	0,001	288,051	0,000000
M6L	1,773	5	0,355	0,633	237	0,003	132,732	0,000000
m7L	0,576	5	0,115	1,304	237	0,006	20,930	0,000000
m8L	0,331	5	0,066	1,276	237	0,005	12,284	0,000000
m9L	0,424	5	0,085	0,877	237	0,004	22,929	0,000000
m10L	0,252	5	0,050	0,744	237	0,003	16,048	0,000000
m11L	0,127	5	0,025	0,203	237	0,001	29,818	0,000000
m12L	1,507	5	0,301	1,327	237	0,006	53,846	0,000000
m13L	1,122	5	0,224	1,193	237	0,005	44,565	0,000000
m14L	1,901	5	0,380	0,827	237	0,003	108,984	0,000000
m15L	1,803	5	0,361	0,792	237	0,003	107,879	0,000000
m16L	2,262	5	0,452	1,211	234	0,005	87,406	0,000000
m17L	0,006	5	0,001	1,421	234	0,006	0,183	0,968972
m18L	0,201	5	0,040	1,222	234	0,005	7,689	0,000001
m19L	1,325	5	0,265	1,284	234	0,005	48,285	0,000000
m20L	1,566	5	0,313	1,432	234	0,006	51,157	0,000000
m21L	0,192	5	0,038	1,517	234	0,006	5,932	0,000035
m22L	0,435	5	0,087	0,971	234	0,004	20,952	0,000000
m23L	0,329	5	0,066	0,693	234	0,003	22,176	0,000000
m24L	0,082	5	0,016	1,502	234	0,006	2,552	0,028490
m25L	0,137	5	0,027	1,381	234	0,006	4,633	0,000473
m26L	0,618	5	0,124	0,877	234	0,004	32,968	0,000000
m27L	0,802	5	0,160	1,100	234	0,005	34,106	0,000000
M2U/M1U	0,019	5	0,004	0,127	242	0,001	7,356	0,000002
M14U/M15U	0,019	5	0,004	0,334	242	0,001	2,776	0,018517
m3L/m1L	0,085	5	0,017	0,563	237	0,002	7,168	0,000003
m6L/m5L	0,075	5	0,015	0,330	237	0,001	10,699	0,000000
F1	145,721	5	29,144	1721,503	242	7,114	4,097	0,001372
F2	7,824	5	1,565	679,278	242	2,807	0,557	0,732554
F3	98,929	5	19,786	577,811	242	2,388	8,287	0,000000
F4	586,210	5	117,242	2406,366	242	9,944	11,791	0,000000
F5	252,803	5	50,561	920,414	242	3,803	13,294	0,000000
F6	6,419	5	1,284	260,678	242	1,077	1,192	0,313781
F7	105,103	5	21,021	1873,590	242	7,742	2,715	0,020810
F8	324,956	5	64,991	1258,401	242	5,200	12,498	0,000000
F9	248,667	5	49,733	814,065	242	3,364	14,784	0,000000
F10	440,272	5	88,054	627,631	242	2,594	33,952	0,000000
F11	20,275	5	4,055	595,327	242	2,460	1,648	0,147932
F12	233,211	5	46,642	1533,339	242	6,336	7,361	0,000002
F13	312,938	5	62,588	9617,314	242	39,741	1,575	0,167794
F14	95,510	5	19,102	883,278	237	3,727	5,125	0,000175

F15	21,676	5	4,335	713,247	237	3,009	1,441	0,210430
F16	26,031	5	5,206	635,605	237	2,682	1,941	0,088294
**F17**	**79,603**	**5**	**15,921**	**918,888**	**237**	**3,877**	**4,106**	**0,001354**
**F18**	**183,936**	**5**	**36,787**	**989,843**	**237**	**4,177**	**8,808**	**0,000000**
F19	37,919	5	7,584	1644,170	237	6,937	1,093	0,364804
F20	10,511	5	2,102	462,572	237	1,952	1,077	0,373680
F21	25,249	5	5,050	563,178	237	2,376	2,125	0,063218
**F22**	**111,565**	**5**	**22,313**	**1294,605**	**234**	**5,533**	**4,033**	**0,001573**
**F23**	**148,904**	**5**	**29,781**	**1478,862**	**234**	**6,320**	**4,712**	**0,000404**
F24	147,306	5	29,461	9586,292	234	40,967	0,719	0,609643
**SURM1**	**39,353**	**5**	**7,871**	**3,645**	**242**	**0,015**	**522,564**	**0,000000**
**SURM2**	**15,594**	**5**	**3,119**	**1,818**	**242**	**0,008**	**415,267**	**0,000000**
**SURm1**	**25,548**	**5**	**5,110**	**1,891**	**237**	**0,008**	**640,262**	**0,000000**
**SURm2**	**8,958**	**5**	**1,792**	**5,839**	**234**	**0,025**	**71,803**	**0,000000**

ANOVA, Recent samples, categorial variables PARATAXON SPC

	SS Effect	*Df Effect*	*MS Effect*	*SS Error*	*Df Error*	*MS Error*	*F*	p
**M1U**	**7,513**	**5**	**1,503**	**1,136**	**242**	**0,005**	**320,051**	**0,000000**
**M2U**	**0,973**	**5**	**0,195**	**0,729**	**242**	**0,003**	**64,570**	**0,000000**
**M3U**	**0,649**	**5**	**0,130**	**0,286**	**242**	**0,001**	**109,892**	**0,000000**
**M4U**	**2,549**	**5**	**0,510**	**0,398**	**242**	**0,002**	**309,883**	**0,000000**
**M5U**	**3,038**	**5**	**0,608**	**0,593**	**242**	**0,002**	**247,888**	**0,000000**
**M6U**	**1,624**	**5**	**0,325**	**1,977**	**242**	**0,008**	**39,770**	**0,000000**
**M7U**	**1,142**	**5**	**0,228**	**1,558**	**242**	**0,006**	**35,489**	**0,000000**
**M8U**	**0,277**	**5**	**0,055**	**0,279**	**242**	**0,001**	**47,955**	**0,000000**
**M9U**	**0,215**	**5**	**0,043**	**0,324**	**242**	**0,001**	**32,058**	**0,000000**
**M10U**	**0,214**	**5**	**0,043**	**0,442**	**242**	**0,002**	**23,444**	**0,000000**
**M11U**	**0,320**	**5**	**0,064**	**0,419**	**242**	**0,002**	**36,925**	**0,000000**
**M12U**	**0,892**	**5**	**0,178**	**0,720**	**242**	**0,003**	**59,923**	**0,000000**
**M13U**	**1,112**	**5**	**0,222**	**0,674**	**242**	**0,003**	**79,804**	**0,000000**
**M14U**	**1,274**	**5**	**0,255**	**0,355**	**242**	**0,001**	**173,690**	**0,000000**
**M15U**	**1,346**	**5**	**0,269**	**0,375**	**242**	**0,002**	**173,756**	**0,000000**
**M16U**	**0,713**	**5**	**0,143**	**0,309**	**242**	**0,001**	**111,567**	**0,000000**
**M17U**	**0,845**	**5**	**0,169**	**0,382**	**242**	**0,002**	**107,047**	**0,000000**
**M18U**	**3,354**	**5**	**0,671**	**0,581**	**242**	**0,002**	**279,510**	**0,000000**
**M19U**	**0,443**	**5**	**0,089**	**0,204**	**242**	**0,001**	**105,215**	**0,000000**
**M20U**	**2,152**	**5**	**0,430**	**0,306**	**242**	**0,001**	**340,462**	**0,000000**
**M21U**	**2,376**	**5**	**0,475**	**0,550**	**242**	**0,002**	**208,906**	**0,000000**
**M22U**	**1,118**	**5**	**0,224**	**1,068**	**242**	**0,004**	**50,673**	**0,000000**
**M23U**	**0,296**	**5**	**0,059**	**0,330**	**242**	**0,001**	**43,402**	**0,000000**
**M24U**	**0,215**	**5**	**0,043**	**0,279**	**242**	**0,001**	**37,355**	**0,000000**
**M25U**	**0,593**	**5**	**0,119**	**0,423**	**242**	**0,002**	**67,842**	**0,000000**
**M26U**	**0,599**	**5**	**0,120**	**0,456**	**242**	**0,002**	**63,594**	**0,000000**
**M27U**	**0,317**	**5**	**0,063**	**0,260**	**242**	**0,001**	**58,873**	**0,000000**
**M28U**	**0,412**	**5**	**0,082**	**0,261**	**242**	**0,001**	**76,351**	**0,000000**
**M29U**	**1,618**	**5**	**0,324**	**0,298**	**242**	**0,001**	**262,786**	**0,000000**
**M30U**	**1,530**	**5**	**0,306**	**0,281**	**242**	**0,001**	**263,841**	**0,000000**
**m1L**	**5,709**	**5**	**1,142**	**0,813**	**237**	**0,003**	**332,818**	**0,000000**
**m2L**	**0,103**	**5**	**0,021**	**1,326**	**237**	**0,006**	**3,665**	**0,003258**
**m3L**	**0,411**	**5**	**0,082**	**1,660**	**237**	**0,007**	**11,733**	**0,000000**
**m4L**	**2,183**	**5**	**0,437**	**1,764**	**237**	**0,007**	**58,667**	**0,000000**
**m5L**	**2,009**	**5**	**0,402**	**0,354**	**237**	**0,001**	**268,859**	**0,000000**
**m6L**	**1,737**	**5**	**0,347**	**0,669**	**237**	**0,003**	**123,171**	**0,000000**
**m7L**	**0,581**	**5**	**0,116**	**1,298**	**237**	**0,005**	**21,219**	**0,000000**
**m8L**	**0,360**	**5**	**0,072**	**1,247**	**237**	**0,005**	**13,671**	**0,000000**
**m9L**	**0,438**	**5**	**0,088**	**0,863**	**237**	**0,004**	**24,065**	**0,000000**
**m10L**	**0,254**	**5**	**0,051**	**0,741**	**237**	**0,003**	**16,270**	**0,000000**
**m11L**	**0,127**	**5**	**0,025**	**0,203**	**237**	**0,001**	**29,559**	**0,000000**
**m12L**	**1,541**	**5**	**0,308**	**1,293**	**237**	**0,005**	**56,504**	**0,000000**
**m13L**	**1,170**	**5**	**0,234**	**1,145**	**237**	**0,005**	**48,397**	**0,000000**
**m14L**	**1,926**	**5**	**0,385**	**0,802**	**237**	**0,003**	**113,863**	**0,000000**
**m15L**	**1,837**	**5**	**0,367**	**0,759**	**237**	**0,003**	**114,733**	**0,000000**
**m16L**	**2,405**	**5**	**0,481**	**1,068**	**234**	**0,005**	**105,370**	**0,000000**
m17L	0,019	5	0,004	1,407	234	0,006	0,633	0,674369
**m18L**	**0,231**	**5**	**0,046**	**1,192**	**234**	**0,005**	**9,090**	**0,000000**
**m19L**	**1,456**	**5**	**0,291**	**1,152**	**234**	**0,005**	**59,130**	**0,000000**
**m20L**	**1,669**	**5**	**0,334**	**1,329**	**234**	**0,006**	**58,743**	**0,000000**

**m21L**	**0,238**	**5**	**0,048**	**1,471**	**234**	**0,006**	**7,578**	**0,000001**
**m22L**	**0,460**	**5**	**0,092**	**0,945**	**234**	**0,004**	**22,794**	**0,000000**
**m23L**	**0,366**	**5**	**0,073**	**0,656**	**234**	**0,003**	**26,115**	**0,000000**
**m24L**	**0,120**	**5**	**0,024**	**1,464**	**234**	**0,006**	**3,849**	**0,002269**
**m25L**	**0,167**	**5**	**0,033**	**1,351**	**234**	**0,006**	**5,781**	**0,000047**
**m26L**	**0,668**	**5**	**0,134**	**0,827**	**234**	**0,004**	**37,798**	**0,000000**
**m27L**	**0,857**	**5**	**0,171**	**1,044**	**234**	**0,004**	**38,414**	**0,000000**
**M2U/M1U**	**0,014**	**5**	**0,003**	**0,132**	**242**	**0,001**	**4,943**	**0,000250**
**M14U/M15U**	**0,019**	**5**	**0,004**	**0,334**	**242**	**0,001**	**2,806**	**0,017478**
**m3L/m1L**	**0,082**	**5**	**0,016**	**0,566**	**237**	**0,002**	**6,876**	**0,000005**
**m6L/m5L**	**0,074**	**5**	**0,015**	**0,331**	**237**	**0,001**	**10,611**	**0,000000**
**F1**	**126,019**	**5**	**25,204**	**1741,206**	**242**	**7,195**	**3,503**	**0,004474**
F2	16,038	5	3,208	671,064	242	2,773	1,157	0,331228
**F3**	**81,523**	**5**	**16,305**	**595,217**	**242**	**2,460**	**6,629**	**0,000008**
**F4**	**518,974**	**5**	**103,795**	**2473,603**	**242**	**10,221**	**10,155**	**0,000000**
**F5**	**269,723**	**5**	**53,945**	**903,495**	**242**	**3,733**	**14,449**	**0,000000**
F6	2,207	5	0,441	264,890	242	1,095	0,403	0,846296
**F7**	**115,021**	**5**	**23,004**	**1863,672**	**242**	**7,701**	**2,987**	**0,012306**
**F8**	**307,122**	**5**	**61,424**	**1276,235**	**242**	**5,274**	**11,647**	**0,000000**
**F9**	**253,515**	**5**	**50,703**	**809,216**	**242**	**3,344**	**15,163**	**0,000000**
**F10**	**442,349**	**5**	**88,470**	**625,553**	**242**	**2,585**	**34,225**	**0,000000**
F11	17,002	5	3,400	598,599	242	2,474	1,375	0,234411
**F12**	**225,670**	**5**	**45,134**	**1540,880**	**242**	**6,367**	**7,088**	**0,000003**
F13	410,377	5	82,075	9519,875	242	39,338	2,086	0,067773
**F14**	**102,431**	**5**	**20,486**	**876,357**	**237**	**3,698**	**5,540**	**0,000076**
F15	23,044	5	4,609	711,879	237	3,004	1,534	0,179831
**F16**	**34,424**	**5**	**6,885**	**627,211**	**237**	**2,646**	**2,602**	**0,025911**
**F17**	**83,953**	**5**	**16,791**	**914,538**	**237**	**3,859**	**4,351**	**0,000830**
**F18**	**166,086**	**5**	**33,217**	**1007,694**	**237**	**4,252**	**7,812**	**0,000001**
F19	50,870	5	10,174	1631,220	237	6,883	1,478	0,197653
F20	14,648	5	2,930	458,436	237	1,934	1,515	0,185963
**F21**	**40,906**	**5**	**8,181**	**547,520**	**237**	**2,310**	**3,541**	**0,004164**
**F22**	**104,305**	**5**	**20,861**	**1301,865**	**234**	**5,564**	**3,750**	**0,002765**
**F23**	**156,169**	**5**	**31,234**	**1471,597**	**234**	**6,289**	**4,967**	**0,000242**
F24	121,684	5	24,337	9611,913	234	41,077	0,592	0,705756
**SURM1**	**39,558**	**5**	**7,912**	**3,440**	**242**	**0,014**	**556,581**	**0,000000**
**SURM2**	**15,998**	**5**	**3,200**	**1,414**	**242**	**0,006**	**547,745**	**0,000000**
**SURm1**	**25,396**	**5**	**5,079**	**2,044**	**237**	**0,009**	**588,925**	**0,000000**
**SURm2**	**9,628**	**5**	**1,926**	**5,168**	**234**	**0,022**	**87,191**	**0,000000**
