# Supplementary material for: Late Pleistocene-Holocene paleobiogeography of the genus Apodemus in Central Europe
Source: PLoS One. 2017 Mar 10;12(3):e0173668. doi: 10.1371/journal.pone.0173668 (PMC5345881; doi:10.1371/journal.pone.0173668)
Supplement: S4 Table — (PDF) [file pone.0173668.s005.pdf]

Supplementary file IV: Details of discrimination functions (comp. Fig. 3 and 7): standardized coefficients and analytical summary.

IV a: Standardized coefficients of the discrimination function based on M1 variables

	Root 1	Root 2	Root 3
**SURM1**	1.532061	0.97754	9.13053
**M1U**	-0.091437	-1.62663	-3.36599
**M2U**	-0.047379	-0.33933	0.48129
**M3U**	0.278626	0.14350	-0.07502
**M4U**	-0.961322	0.29267	-5.22367
**M5U**	0.447602	0.24803	-0.03434
**M6U**	0.481392	-0.59029	-0.62174
**M7U**	-0.207823	0.21353	0.52032
**M8U**	0.358610	-0.52942	0.20377
**M9U**	-0.313234	0.09718	0.09352
**M10U**	0.180212	-0.01503	0.28010
**M11U**	0.351260	-0.13144	0.26678
**M12U**	-0.244694	0.38453	-0.35782
**M13U**	-0.158456	-0.23824	-1.21724
**M14U**	-0.003394	0.17763	-0.56716
**M15U**	0.187535	0.54947	-0.23004
**M16U**	-0.149527	0.15519	-0.25986
**M17U**	-0.239426	-0.36174	-0.52292
**Eigenval**	9.670592	1.97190	0.85506
**Cum.Prop**	0.773799	0.93158	1.00000

IV b: Analytical summary of the discriminant function based on M1 variables
(Wilks' Lambda: ,09705 approx. F (162,6666)=13,734 p<0,0000)

	Wilks' Lambda	Partial Lambda	F-remove	p-level	Toler.	1-Toler. (R-Sqr.)
**SURM1**	0,136367	0,711696	36,95357	0,000000	0,003874	0,996126
**M1U**	0,113164	0,857627	15,14365	0,000000	0,005437	0,994563
**M2U**	0,099718	0,973266	2,50569	0,007857	0,057631	0,942369
**M3U**	0,100712	0,963664	3,43961	0,000358	0,242809	0,757191
**M4U**	0,130129	0,745816	31,08968	0,000000	0,013047	0,986953
**M5U**	0,106906	0,907825	9,26219	0,000000	0,171068	0,828932
**M6U**	0,102218	0,949467	4,85507	0,000002	0,198152	0,801848
**M7U**	0,100356	0,967081	3,10517	0,001113	0,190152	0,809848
**M8U**	0,103137	0,941003	5,71927	0,000000	0,648421	0,351579
**M9U**	0,100633	0,964416	3,36583	0,000461	0,509013	0,490987
**M10U**	0,098303	0,987273	1,17597	0,307015	0,290440	0,709560

**M11U**	0,099976	0,970755	2,74818	0,003614	0,282995	0,717005
**M12U**	0,098943	0,980886	1,77756	0,068752	0,064883	0,935117
**M13U**	0,100098	0,969576	2,86242	0,002490	0,100340	0,899660
**M14U**	0,099910	0,971396	2,68612	0,004418	0,076802	0,923198
**M15U**	0,098735	0,982953	1,58203	0,116193	0,091985	0,908015
**M16U**	0,098795	0,982364	1,63769	0,100390	0,155527	0,844473
**M17U**	0,100547	0,965245	3,28463	0,000608	0,127906	0,872094

IV d: Analytical summary of the discriminant function based on m1 variables
(Wilks' Lambda: ,09290 approx. F (216,8521)=12,795 p<0,0000)

	**Wilks' Lambda**	**Partial Lambda**	**F-remove**	**p-level**	**Toler.**	**1-Toler. (R-Sqr.)**
**SURm1**	0,101186	0,918084	9,96350	0,000000	0,004285	0,995715
**m1L**	0,106337	0,873609	16,15562	0,000000	0,014921	0,985079
**m2L**	0,093245	0,996271	0,41799	0,926013	0,146053	0,853947
**m3L**	0,094668	0,981292	2,12889	0,024808	0,109253	0,890747
**m4L**	0,095213	0,975671	2,78444	0,003155	0,240415	0,759585
**m5L**	0,099669	0,932057	8,14003	0,000000	0,012856	0,987144
**m6L**	0,098539	0,942742	6,78217	0,000000	0,289851	0,710149
**m7L**	0,095384	0,973922	2,99002	0,001599	0,344611	0,655389
**m8L**	0,097258	0,955157	5,24260	0,000001	0,241259	0,758741
**m9L**	0,094362	0,984473	1,76121	0,071581	0,342852	0,657148
**m10L**	0,105112	0,883792	14,68280	0,000000	0,461408	0,538592
**m11L**	0,096758	0,960099	4,64079	0,000005	0,522552	0,477448
**m12L**	0,094734	0,980606	2,20845	0,019517	0,166052	0,833948
**m13L**	0,094892	0,978977	2,39800	0,010881	0,235760	0,764240
**m14L**	0,097531	0,952482	5,57091	0,000000	0,152786	0,847215
**m15L**	0,096323	0,964428	4,11869	0,000032	0,225108	0,774892
**F14**	0,098720	0,941013	6,99973	0,000000	0,776368	0,223632
**F21**	0,097367	0,954087	5,37371	0,000000	0,784938	0,215062
**F20**	0,101546	0,914821	10,39727	0,000000	0,666807	0,333193
**F19**	0,095710	0,970605	3,38189	0,000424	0,459020	0,540980
**F18**	0,099997	0,928999	8,53439	0,000000	0,465994	0,534006
**F17**	0,100044	0,928564	8,59076	0,000000	0,326170	0,673830
**F16**	0,100609	0,923350	9,26983	0,000000	0,480891	0,519109
**F15**	0,096169	0,965972	3,93362	0,000062	0,899634	0,100366
