# Supplementary material for: Late Pleistocene-Holocene paleobiogeography of the genus Apodemus in Central Europe
Source: PLoS One. 2017 Mar 10;12(3):e0173668. doi: 10.1371/journal.pone.0173668 (PMC5345881; doi:10.1371/journal.pone.0173668)
Supplement: S5 Table — (PDF) [file pone.0173668.s006.pdf]

Supplementary file V: List of material and representation of particular parataxa in individual samples. For list of localities and abbreviations see Tab. 1, definition of parataxa see Tab. 2. STRAT – biostratigraphic setting in terms of Holocene biozones of Horáček and Ložek (1988) – see text for details.

			The total number of molars				*A.uralensis* (Parataxon 1)				*A.ural./sylv.* (Parataxon 2)				*A.sylvaticus* (Parataxon 3)				*A.sylv./flav.* (Parataxon 4)				*A.flavicollis* (Parataxon 5,6)				*A.agrarius* (*Parataxon* 8)				Number of unidentified			
LOC	LAY	STRA	M1	M2	m1	m2	M1	M2	m1	m2	M1	M2	m1	m2	M1	M2	m1	m2	M1	M2	m1	m2	M1	M2	m1	m2	M1	M2	m1	m2	M1	M2	m1	m2
AKSA	50-70	F3	4	5	9	5	-	-	-	-	-	-	-	-	1	1	1	-	-	1	1	1	3	3	6	4	-	-	-	-	-	-	1	-
AKSA	70-110	F2	-	-	2	1	-	-	-	-	-	-	-	-	-	-	-	-	-	-	-	-	-	-	1	1	-	-	-	-	-	-	1	-
AKSA	110-140	E	2	1	-	1	-	-	-	-	-	-	-	-	-	-	-	-	-	-	-	-	2	1	-	1	-	-	-	-	-	-	-	-
AKSA	140-170	D	7	-	4	3	-	-	-	-	-	-	-	-	-	-	-	-	-	-	-	-	7	-	4	3	-	-	-	-	-	-	-	-
AKSA	170-250	B	6	2	12	3	1	-	2	-	-	-	-	-	-	-	-	-	-	-	1	-	5	2	9	3	-	-	-	-	-	-	-	-
BACI	I/A1	F	-	-	5	1	-	-	-	-	-	-	-	-	-	-	-	-	-	-	-	-	-	-	3	1	-	-	-	-	-	-	2	-
BACI	I/A2	F	4	2	9	5	-	-	-	-	-	-	-	-	-	-	1	-	-	-	-	-	4	2	8	5	-	-	-	-	-	-	-	-
BACI	130-140	C2	7	4	10	8	2	2	-	-	-	-	-	-	-	-	-	-	-	-	1	1	5	2	9	7	-	-	-	-	-	-	-	-
BACI	I 230	C1	6	1	7	5	1	-	-	-	-	-	-	-	-	-	-	-	-	-	-	-	5	1	7	5	-	-	-	-	-	-	-	-
BACI	I/3	C	6	7	16	8	-	-	2	-	-	-	-	-	-	-	-	-	-	2	3	1	6	5	11	7	-	-	-	-	-	-	-	-
BACI	II/3	C	1	1	-	-	-	-	-	-	-	-	-	-	-	-	-	-	-	-	-	-	1	1	-	-	-	-	-	-	-	-	-	-
BACI	I/A6	C	11	3	12	4	-	-	-	-	-	-	-	-	-	-	-	-	-	-	-	-	11	3	10	4	-	-	-	-	-	-	2	-
MART	105-140	E	5	3	5	3	-	-	-	-	-	-	-	-	-	-	-	-	-	-	-	-	5	2	4	3	-	-	-	-	-	1	1	-
MART	140-170	D	13	5	12	3	-	-	-	-	-	-	-	-	-	-	1	-	1	-	4	-	11	4	7	3	-	-	-	-	1	1	-	-
MART	190-210	C2	4	3	9	8	-	-	-	-	-	-	-	-	-	-	-	-	-	-	1	-	4	3	6	7	-	-	-	-	-	-	2	1
MART	240-270	C1	2	3	4	6	-	-	-	-	-	-	-	-	-	-	-	-	-	-	1	-	2	2	3	6	-	-	-	-	-	1	-	-
MART	D	B	3	4	10	8	-	-	-	-	-	-	-	-	-	-	-	-	-	-	3	1	3	4	7	7	-	-	-	-	-	-	-	-
MART	E	B	74	30	76	47	1	-	-	-	-	-	-	-	1	-	2	-	11	4	1	3	60	24	69	43	-	-	-	-	1	2	4	1
MART	F	A	9	7	14	7	2	-	1	-	-	-	-	-	-	-	-	-	2	1	1	-	4	5	12	7	-	-	-	-	1	1	-	-
MART	G	A	2	1	5	3	-	-	-	-	-	-	2	2	-	-	-	-	-	-	-	-	2	1	2	1	-	-	-	-	-	-	1	-
SKAC	1	F3	7	4	11	7	-	-	-	-	-	-	1	-	4	1	3	1	-	-	-	-	3	3	7	6	-	-	-	-	-	-	-	-
SKAC	2	F2	7	4	6	4	-	-	-	-	-	-	-	-	2	1	4	-	-	1	-	1	5	2	1	2	-	-	-	-	-	-	1	1
SKAC	3	E	16	5	14	5	-	-	-	-	-	-	1	1	-	-	7	-	5	-	1	1	11	5	4	1	-	-	1	1	-	-	-	1
SKAC	4	E	8	10	13	13	1	1	1	-	-	-	-	-	-	-	2	-	2	2	-	1	5	6	9	9	-	-	-	-	-	1	1	3
SKAC	5	D	13	6	7	8	-	-	-	-	-	-	-	-	1	-	-	-	2	1	-	1	9	5	7	5	-	-	-	-	1	-	-	2
SKAC	6	D	10	3	6	4	-	-	-	-	-	-	-	-	-	-	-	-	-	-	-	-	10	3	6	3	-	-	-	-	-	-	-	1
SKAC	7	C	1	-	1	1	-	-	-	-	-	-	-	-	-	-	-	-	-	-	-	-	1	-	1	1	-	-	-	-	-	-	-	-
SKAM	3b	C2	2	1	3	4	-	-	-	-	-	-	-	-	-	-	-	-	-	-	-	-	2	1	3	4	-	-	-	-	-	-	-	-
SKAM	4	C1	4	1	5	3	1	-	1	-	-	-	1	2	-	-	-	-	-	-	-	-	2	-	3	1	-	-	-	-	1	1	-	-
SKAM	5	B	2	1	1	1	-	-	-	-	-	-	-	-	-	-	-	-	-	-	-	-	1	1	1	1	-	-	-	-	1	-	-	-
ZELE	5	E	1	-	5	1	-	-	-	-	-	-	-	-	-	-	-	-	-	-	-	-	-	-	5	1	-	-	-	-	1	-	-	-
ZELE	6	D	3	2	2	1	-	-	-	-	-	-	-	-	-	-	-	-	1	-	-	-	2	2	2	1	-	-	-	-	-	-	-	-
ZELE	7	C	1	-	2	1	-	-	-	-	-	-	-	-	-	-	-	-	-	-	-	-	1	-	2	1	-	-	-	-	-	-	-	-
PCER	6	C	2	1	13	8	-	-	-	-	-	-	-	-	-	-	-	-	-	-	-	-	2	1	12	8	-	-	-	-	-	-	1	-
BARO	65-80	F	8	3	5	1	-	-	-	-	-	-	-	-	-	-	-	-	-	-	-	-	5	3	5	1	-	-	-	-	3	-	-	-
BARO	A1	F	3	-	3	2	-	-	-	-	-	-	-	-	1	-	-	1	-	-	2	-	2	-	1	1	-	-	-	-	-	-	-	-
BARO	5	E	1	-	2	1	-	-	-	-	-	-	-	-	-	-	-	-	-	-	1	-	-	-	1	1	-	-	-	-	1	-	-	-
BARO	A7	D	-	-	1	-	-	-	-	-	-	-	-	-	-	-	-	-	-	-	-	-	-	-	1	-	-	-	-	-	-	-	-	-
BARO	11	A	-	-	1	-	-	-	-	-	-	-	-	-	-	-	-	-	-	-	-	-	-	-	1	-	-	-	-	-	-	-	-	-
HOLS	1	E	-	1	15	6	-	-	-	-	-	-	-	-	-	-	2	-	-	-	1	3	-	-	10	3	-	1	-	-	-	-	2	-
HOLS	2	D	5	3	3	1	-	-	-	-	-	-	-	-	-	-	-	-	-	-	-	-	5	3	3	1	-	-	-	-	-	-	-	-
NEMC	3	D	4	4	9	2	-	-	-	-	-	-	-	-	-	-	1	1	1	-	-	-	3	4	8	1	-	-	-	-	-	-	-	-
NEMC	4	D	5	3	9	1	-	-	-	-	-	-	-	-	-	-	-	-	-	-	-	-	4	3	9	1	-	-	-	-	1	-	-	-
NEMC	5	C2	1	-	2	2	-	-	-	-	-	-	-	-	-	-	-	-	-	-	-	-	1	-	2	2	-	-	-	-	-	-	-	-
SRNC	2	D	6	5	10	5	-	-	-	-	-	-	-	-	-	-	-	-	-	-	1	-	5	5	6	5	-	-	-	-	1	-	3	-
SRNC	3	C2	1	1	2	2	-	-	-	1	-	-	-	-	-	-	-	-	1	-	-	-	-	1	2	1	-	-	-	-	-	-	-	-
SRNC	4	C2	-	1	-	1	-	-	-	-	-	-	-	-	-	-	-	-	-	-	-	-	-	1	-	1	-	-	-	-	-	-	-	-
SRNC	5	C2	2	-	1	1	-	-	-	-	-	-	-	-	-	-	-	-	-	-	-	-	2	-	1	1	-	-	-	-	-	-	-	-
ZAZD	5	D	-	-	2	-	-	-	-	-	-	-	-	-	-	-	-	-	-	-	-	-	-	-	2	-	-	-	-	-	-	-	-	-
ZAZD	6	D	2	1	-	-	-	-	-	-	-	-	-	-	-	-	-	-	-	-	-	-	2	-	-	-	-	-	-	-	-	1	-	-
ZAZD	7	C2	-	2	1	-	-	-	-	-	-	-	-	-	-	-	-	-	-	-	-	-	-	2	1	-	-	-	-	-	-	-	-	-
ZAZD	8	C2	-	-	1	1	-	-	-	-	-	-	-	-	-	-	-	-	-	-	-	-	-	-	1	1	-	-	-	-	-	-	-	-
ZAZD	9a	C1	-	-	-	1	-	-	-	-	-	-	-	-	-	-	-	-	-	-	-	-	-	-	-	1	-	-	-	-	-	-	-	-
ZKAZ	D 8b	A	-	-	2	1	-	-	-	-	-	-	-	-	-	-	-	-	-	-	-	-	-	-	2	1	-	-	-	-	-	-	-	-
PRUC	3	F	1	-	1	1	-	-	-	-	-	-	-	-	1	-	-	-	-	-	-	-	-	-	1	1	-	-	-	-	-	-	-	-
PRUC	I1	F	1	-	1	2	-	-	-	-	-	-	-	-	-	-	-	-	-	-	-	-	1	-	1	2	-	-	-	-	-	-	-	-
PRUC	I5	D	2	1	7	3	-	-	-	-	-	-	-	-	1	-	1	-	-	1	-	-	1	-	5	3	-	-	-	-	-	-	1	-
MARK	R2	E	1	-	-	-	1	-	-	-	-	-	-	-	-	-	-	-	-	-	-	-	-	-	-	-	-	-	-	-	-	-	-	-
MARK	R3	D	-	-	1	-	-	-	-	-	-	-	-	-	-	-	-	-	-	-	-	-	-	-	1	-	-	-	-	-	-	-	-	-
MARK	R6	C	-	-	1	-	-	-	-	-	-	-	-	-	-	-	-	-	-	-	1	-	-	-	-	-	-	-	-	-	-	-	-	-
MARK	R7	B	1	-	-	-	-	-	-	-	-	-	-	-	-	-	-	-	-	-	-	-	1	-	-	-	-	-	-	-	-	-	-	-
MEDV	1	F3	2	-	1	2	-	-	-	-	-	-	-	-	-	-	-	-	-	-	-	-	1	-	1	2	1	-	-	-	-	-	-	-
MEDV	2	F2	3	2	4	3	-	-	-	-	-	-	-	-	-	1	-	-	1	-	-	-	2	1	3	3	-	-	-	-	-	-	1	-
MEDV	3	F1	7	4	8	3	-	-	-	-	-	-	-	-	-	-	2	-	-	-	-	-	7	4	5	3	-	-	-	-	-	-	1	-
MEDV	4	E	4	4	2	1	-	-	-	-	-	-	-	-	-	-	-	-	-	-	-	-	3	4	2	-	-	-	-	-	1	-	-	1
MEDV	5	E	-	-	2	2	-	-	-	-	-	-	-	-	-	-	-	-	-	-	-	-	-	-	2	2	-	-	-	-	-	-	-	-
MEDV	6	E	2	-	-	2	-	-	-	-	-	-	-	1	-	-	-	-	1	-	-	-	1	-	-	1	-	-	-	-	-	-	-	-
MEDV	7	D	1	1	2	-	-	-	-	-	-	-	-	-	-	-	-	-	-	-	-	-	1	1	2	-	-	-	-	-	-	-	-	-
MEDV	G1	C	1	-	1	1	-	-	-	-	-	-	-	-	-	-	-	-	-	-	-	-	1	-	1	1	-	-	-	-	-	-	-	-
MEDV	G2	C	1	-	-	-	-	-	-	-	-	-	-	-	-	-	-	-	-	-	-	-	1	-	-	-	-	-	-	-	-	-	-	-
PESK	2	F	6	2	9	6	1	1	1	-	-	-	-	-	-	-	-	-	-	-	1	1	5	1	7	5	-	-	-	-	-	-	-	-
PESK	3	E	9	6	7	8	2	1	-	-	-	-	-	-	1	1	-	-	-	1	-	-	5	3	5	8	-	-	-	-	1	-	2	-
PESK	4a	D	2	1	3	2	-	-	1	-	-	-	-	-	-	-	-	-	-	-	-	-	2	1	2	1	-	-	-	-	-	-	-	1
PESK	4b	C2	-	-	4	-	-	-	1	-	-	-	-	-	-	-	-	-	-	-	-	-	-	-	2	-	-	-	-	-	-	-	1	-
PESK	4	C	-	-	4	-	-	-	1	-	-	-	-	-	-	-	-	-	-	-	-	-	-	-	3	-	-	-	-	-	-	-	-	-
PESK	5	C2	-	-	2	-	-	-	-	-	-	-	-	-	-	-	-	-	-	-	1	-	-	-	1	-	-	-	-	-	-	-	-	-
PESK	6	C1	-	-	1	1	-	-	1	-	-	-	-	1	-	-	-	-	-	-	-	-	-	-	-	-	-	-	-	-	-	-	-	-
PESK	7	B	-	-	1	-	-	-	-	-	-	-	-	-	-	-	-	-	-	-	-	-	-	-	1	-	-	-	-	-	-	-	-	-
PESK	8	B	1	-	-	-	-	-	-	-	-	-	-	-	-	-	-	-	-	-	-	-	-	-	-	-	-	-	-	-	1	-	-	-
CESK	II/3	F1	12	8	10	7	-	-	-	-	-	-	-	-	-	-	-	1	1	2	3	-	6	4	4	4	5	2	3	2	-	-	-	-
CESK	II/4	F1	18	12	4	2	-	-	-	-	-	-	-	-	-	-	-	-	4	-	-	-	11	10	3	1	2	1	1	1	1	1	-	-
CESK	II/5	E	-	1	2	2	-	-	-	-	-	-	-	-	-	-	-	-	-	-	-	-	-	1	1	2	-	-	1	-	-	-	-	-
CESK	3	E	2	-	4	3	-	-	-	-	-	-	-	-	-	-	1	-	-	-	-	-	1	-	2	2	1	-	1	1	-	-	-	-
CESK	4	D	3	1	9	7	-	-	-	-	-	-	-	-	-	-	-	-	-	-	3	-	2	1	4	6	-	-	2	1	1	-	-	-
CESK	5	C2	2	1	4	5	-	-	-	-	-	-	-	1	-	-	-	-	-	-	-	-	2	1	3	3	-	-	-	1	-	-	1	-
CESK	6	C2	8	4	13	8	1	-	1	-	-	-	1	-	-	-	-	-	-	1	2	-	3	2	3	4	4	1	6	4	-	-	-	-
HAMO	1	F	-	-	27	26	-	-	-	-	-	-	-	-	-	-	4	1	-	-	2	2	-	-	20	22	-	-	-	-	-	-	1	1
HAMO	2	F	13	3	18	14	-	-	-	-	-	-	-	-	-	-	2	-	2	-	1	1	9	3	11	11	-	-	1	2	2	-	3	-
HAMO	3	F	3	1	9	4	-	-	-	-	-	-	-	-	1	1	-	-	-	-	1	-	1	-	8	4	-	-	-	-	1	-	-	-
HAMO	4	E	11	5	11	5	-	-	-	-	-	-	-	-	-	-	-	-	1	-	2	1	10	5	9	3	-	-	-	-	-	-	-	1
HAMO	5	E	5	2	9	7	-	-	-	-	-	-	-	1	-	-	-	-	1	-	-	-	4	1	9	6	-	-	-	-	-	1	-	-
HAMO	12	A	-	-	-	1	-	-	-	-	-	-	-	-	-	-	-	-	-	-	-	1	-	-	-	-	-	-	-	-	-	-	-	-
CEMN	2	F	-	-	2	3	-	-	-	-	-	-	-	1	-	-	-	-	-	-	-	-	-	-	1	1	-	-	1	-	-	-	-	1
CEMN	3a	F	-	-	1	-	-	-	-	-	-	-	-	-	-	-	-	-	-	-	-	-	-	-	1	-	-	-	-	-	-	-	-	-
CEMN	3b	F	-	1	1	-	-	-	-	-	-	-	-	-	-	-	-	-	-	-	-	-	-	1	1	-	-	-	-	-	-	-	-	-
CEMN	4a	E	8	5	9	4	-	-	-	-	-	-	-	-	-	-	-	-	-	-	-	-	7	5	9	4	-	-	-	-	1	-	-	-
CEMN	4b	D	19	8	16	4	1	-	-	-	-	-	-	-	-	-	-	-	-	1	1	-	18	7	13	4	-	-	-	-	-	-	2	-
CEMN	5	C	22	17	26	18	-	-	2	2	-	-	-	-	1	-	1	-	2	1	1	-	19	16	20	16	-	-	-	-	-	-	2	-
MAST	0	F3	95	121	89	23	-	-	2	-	-	-	-	-	4	2	9	-	8	20	7	1	81	98	69	20	-	-	1	2	2	1	1	-
MAST	1	F	8	3	8	1	-	-	1	-	-	-	-	-	1	-	-	-	-	-	2	1	5	-	4	-	1	1	1	-	1	2	-	-
MAST	2	F	14	2	16	-	-	-	-	-	-	-	-	-	-	-	3	-	-	-	2	-	12	2	5	-	1	-	4	-	1	-	2	-
MAST	3	E	-	-	18	7	-	-	-	-	-	-	-	-	-	-	-	-	-	-	-	2	-	-	12	5	-	-	2	-	-	-	4	-
MAST	4/I	E	-	-	25	9	-	-	-	-	-	-	-	-	-	-	-	-	-	-	5	1	-	-	18	8	-	-	-	-	-	-	2	-
MAST	4/II	E	31	7	34	1	-	-	-	-	-	-	-	-	1	-	-	-	1	-	1	-	27	6	33	1	-	-	-	-	2	1	-	-
MAST	5	E	9	2	18	1	-	-	-	-	-	-	-	-	-	-	-	-	-	-	1	-	9	2	16	1	-	-	-	-	-	-	1	-
MAST	6	D	2	2	4	1	-	-	-	-	-	-	-	-	-	-	1	-	-	-	-	-	2	1	3	1	-	-	-	-	-	1	-	-
MAST	7	D	15	5	14	6	-	-	-	-	-	-	-	-	1	-	-	-	-	-	-	-	14	5	12	6	-	-	-	-	-	-	2	-
MAST	8	D	19	5	27	6	-	-	-	-	-	-	-	-	1	-	2	-	3	-	2	-	13	5	23	6	-	-	-	-	2	-	-	-
MAST	9	C2	40	14	49	32	-	-	-	-	-	-	-	-	1	-	-	-	1	2	2	-	29	12	44	30	-	-	-	-	9	-	3	2
MAST	10	B	14	2	23	6	-	-	-	-	-	-	-	-	-	-	-	-	-	-	-	-	13	2	20	5	-	-	-	-	1	-	3	1
MAST	11	A	1	-	1	-	-	-	-	-	-	-	-	-	-	-	-	-	-	-	-	-	-	-	1	-	-	-	-	-	1	-	-	-
RUZI	2	F	-	1	-	-	-	-	-	-	-	-	-	-	-	-	-	-	-	-	-	-	-	1	-	-	-	-	-	-	-	-	-	-
RUZI	3	E	1	-	2	1	-	-	-	-	-	-	-	-	-	-	-	-	-	-	-	-	1	-	2	1	-	-	-	-	-	-	-	-
RUZI	4	D	1	-	1	-	-	-	-	-	-	-	-	-	-	-	-	-	-	-	-	-	1	-	1	-	-	-	-	-	-	-	-	-
RUZI	7	B	5	1	4	4	-	-	-	-	-	-	-	-	-	-	-	-	-	-	1	-	5	1	3	3	-	-	-	-	-	-	-	1
**TOTAL**			**707**	**399**	**949**	**473**	**15**	**5**	**18**	**3**	**0**	**0**	**6**	**10**	**24**	**8**	**50**	**5**	**52**	**41**	**65**	**25**	**560**	**323**	**730**	**396**	**15**	**6**	**25**	**15**	**41**	**16**	**55**	**19**
